# Designed Metal-ATCUN Derivatives: Redox- and Non-redox-Based Applications Relevant for Chemistry, Biology, and Medicine

**DOI:** 10.1016/j.isci.2020.101792

**Published:** 2020-11-10

**Authors:** Biplab K. Maiti, Nidhi Govil, Taraknath Kundu, José J.G. Moura

**Affiliations:** 1 National Institute of Technology Sikkim, Ravangla Campus, Barfung Block, Ravangla Sub Division, South Sikkim 737139, India; 2 LAQV-REQUIMTE, Departamento de Química, Faculdade de Ciências e Tecnologia, Universidade Nova de Lisboa, *Campus* de Caparica, 2829-516 Caparica, Portugal

**Keywords:** Chemistry, Inorganic Chemistry, Biochemistry, Medical Biochemistry

## Abstract

The designed “ATCUN” motif (amino-terminal copper and nickel binding site) is a replica of naturally occurring ATCUN site found in many proteins/peptides, and an attractive platform for multiple applications, which include nucleases, proteases, spectroscopic probes, imaging, and small molecule activation. ATCUN motifs are engineered at periphery by conjugation to recombinant proteins, peptides, fluorophores, or recognition domains through chemically or genetically, fulfilling the needs of various biological relevance and a wide range of practical usages. This chemistry has witnessed significant growth over the last few decades and several interesting ATCUN derivatives have been described. The redox role of the ATCUN moieties is also an important aspect to be considered. The redox potential of designed M-ATCUN derivatives is modulated by judicious choice of amino acid (including stereochemistry, charge, and position) that ultimately leads to the catalytic efficiency. In this context, a wide range of M-ATCUN derivatives have been designed purposefully for various redox- and non-redox-based applications, including spectroscopic probes, target-based catalytic metallodrugs, inhibition of amyloid-β toxicity, and telomere shortening, enzyme inactivation, biomolecules stitching or modification, next-generation antibiotic, and small molecule activation.

## Graphical Abstract

**Figure:**
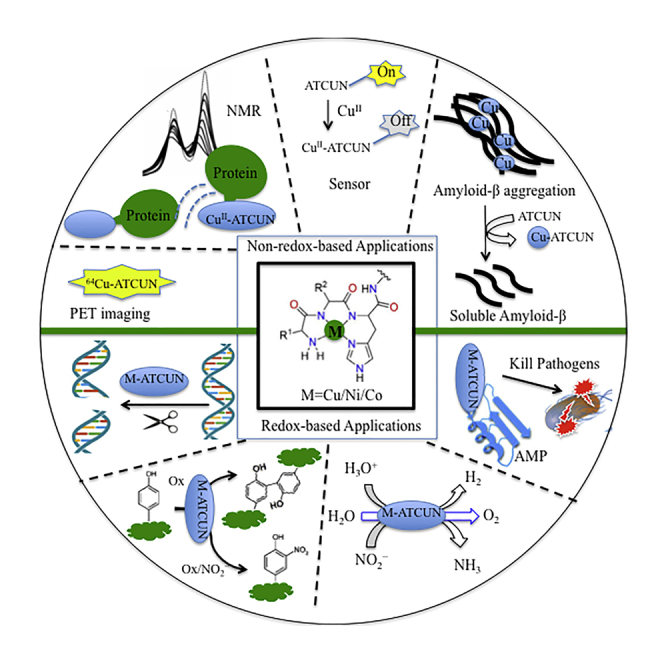

## Introduction

Over the last few decades, the research on designed metal-ATCUN derivatives (ATCUN; amino terminal copper and nickel binding motif possessing H_2_N–X-X-His sequence; X = any amino acid) has greatly progressed due to the wide range of applications envisaged, relevant for biology and chemistry, which include DNA (Harford and Sarkar, 1997; Mack and Dervan, 1992; Jin and Cowan, 2005, 2007; Agbale et al., 2016; Yu and Cowan, 2017a, 2017b; Gonzalez et al., 2018), RNA (Yu and Cowan, 2017a, 2017b; Gonzalez et al., 2018; Ross et al., 2017), proteins (Agbale et al., 2016; Yu and Cowan, 2017a, 2017b; Gonzalez et al., 2018; Pinkham et al., 2018a, 2018b), sugar (Yu and Cowan, 2017a, 2017b), and lipids cleavage (Alexander et al., 2017), antitumor (Kimoto et al., 1983), and antimicrobial activity (Alexander et al., 2018, 2019), inhibition of enzyme activity (Gokhale et al., 2008), telomere shortening (Yu et al., 2015), aggregation of amyloid-β peptides (Zhang et al., 2016), nitration of tyrosine (Maiti et al., 2019), protein-protein cross-linking (Horowitz et al., 2012; Brown et al., 1998), water oxidation (Deng et al., 2018), hydrogen evolution (Kandemir et al., 2016), nitrite to NH_3_ (Guo et al., 2018), useful in spectroscopic probes (Donaldson et al., 2001; Maiti et al., 2017b; Deng et al., 2019a, 2019b; Torrado et al., 1998), and imaging (Miyamoto et al., 2016).

The storyline started in 1960s, when ATCUN motif was first found in human serum albumin (HSA) (Harford and Sarkar, 1997; Laussac and Sarkar, 1984). Since then, it was found in a large number of other naturally occurring proteins/peptides, including BSA (Harford and Sarkar, 1997; Laussac and Sarkar, 1984), hepcidin (Fleming and Sly, 2001), neuromedins C and K (Harford and Sarkar, 1995), human sperm protamine P2a (Mckay et al., 1986), and histatins (Conklin et al., 2017). During this period, the understanding of structure and function of this metal binding ATCUN motif in proteins and peptides was intensively investigated (Harford and Sarkar, 1997; Mack and Dervan, 1992; Jin and Cowan, 2005, 2007; Agbale et al., 2016; Yu and Cowan, 2017a, 2017b; Gonzalez et al., 2018; Alexander et al., 2018, 2019). The structure of ATCUN motif in albumin is well characterized, and its primary function is assigned to the Cu-transport in blood (Harford and Sarkar, 1997; Laussac and Sarkar, 1984). Therefore, the design of ATCUN motif is of interest for mimicking the structure and function of naturally occurring proteins and peptides, as well as for other new performances. Many efforts are being made in designing ATCUN motifs with amino acid variants, as well as its conjugation with protein, peptide, or fluorophore for fulfilling the needs of various practical and biological relevant usages (Gonzalez et al., 2018; Pinkham et al., 2018a, 2018b; Donaldson et al., 2001; Maiti et al., 2017a; Deng et al., 2019a, 2019b; Torrado et al., 1998; Miyamoto et al., 2016). The designed parameters include stereochemistry, position, and charge of amino acid, modulating the redox potential of the metal center in M-ATCUN derivatives (M = Cu^II^, Ni^II^, and Co^II^), leading to catalytic efficiency (Jin and Cowan, 2005, 2007).

In this context, a wide range of applications of M-ATCUN derivatives can be classified into two classes: redox- and non-redox-based applications. However, Cu-ATCUN and Ni-ATCUN derivatives (little information has been so far reported on Co-ATCUN) are mostly introduced for cleavage or modification of biomolecules (such as DNA, RNA, proteins, and amino acids) that are catalyzed by a redox-dependent mechanism (Harford and Sarkar, 1997; Mack and Dervan, 1992; Jin and Cowan, 2005, 2007; Agbale et al., 2016; Yu and Cowan, 2017a, 2017b; Gonzalez et al., 2018). The cleavage of biomolecules by M-ATCUN derivatives is not a random process. This chemistry is designed intentionally, where catalytic center (M-ATCUN) is coupled with the target domain to yield a cocktail complex that selectively binds and oxidatively modifies the therapeutically relevant biomolecules (Yu and Cowan, 2017a, 2017b; Gonzalez et al., 2018; Yu et al., 2015; Joyner and Cowan, 2011). In addition, M-ATCUN derivatives also perform a variety of small molecule activation processes (Deng et al., 2018; Kandemir et al., 2016; Guo et al., 2018).

On the other hand, ATCUN motifs stabilized in a particular oxidation state of metal ion, specially Cu^II^-ATCUN derivatives, are considered as a useful spectroscopic probes such as paramagnetic nuclear magnetic resonance (NMR) (Maiti et al., 2017b), sensor (Torrado et al., 1998), and imaging (Miyamoto et al., 2016). Due to high stability of Cu^II^-ATCUN complex, the ATCUN motif can be used as a Cu-chelation therapy, such as inhibition of aggregation of amyloid-β peptides (Zhang et al., 2016). The combination of ATCUN motif with selected recombinant proteins, peptides, or fluorophores are also used in order to understand the structure and function of biomolecules (Donaldson et al., 2001; Maiti et al., 2017b; Deng et al., 2019a, 2019b; Torrado et al., 1998; Miyamoto et al., 2016).

Over the decades, designed M-ATCUN derivatives rely on various redox- and non-redox-based applications, especially spectroscopic probes, target-based catalytic metallodrugs, inhibition of amyloid-β toxicity and telomerase activity, biomolecules stitching or modification, next-generation antibiotic and small molecules activation. With such a wide range of applications, it is clear that interest in the ATCUN motif is likely to expand in various field in the coming years. Review highlights on the rational design, redox chemistry, and applications of M-ATCUN derivatives in different fields, and viewpoint on this emerging field.

## Biological Role of M-ATCUN Motif in Proteins/Peptides

The metal-binding ATCUN motif is present in many natural proteins and peptides, which plays a fundamental role in metal homeostasis under physiological conditions (Harford and Sarkar, 1997; Laussac and Sarkar, 1984; Fleming and Sly, 2001). HSA is one of the most important copper transport proteins in blood, known as labile Cu pool in extracellular space, and ensures copper homeostasis in human body by utilizing the ATCUN tag (Harford and Sarkar, 1997; Laussac and Sarkar, 1984). Otherwise, dysregulation of Cu homeostasis leads to several number of human diseases, which are WD, cancer, diabetes, and Alzheimer's disease (AD) (Lowe et al., 2017; Maiti and Moura, 2020). Histamin is one class of natural host defense peptide, possessing ATCUN site that involves in antimicrobial activity (Conklin et al., 2017). Hepcidin-25, an iron-regulatory hormone containing ATCUN site, is responsible for iron homeostasis in mammals (Fleming and Sly, 2001). Another class of peptides with ATCUN site, neuromedin C, a neurotransmitter, acts as a growth factor for some tumors and also plays a key role in metal homeostasis in the central nervous system (Harford and Sarkar, 1995). Therefore, these findings provide inspiration to design synthetic ATCUN derivatives with useful metal-binding properties as well as reactivity.

## Design of ATCUN Derivatives

**Figure 1:**
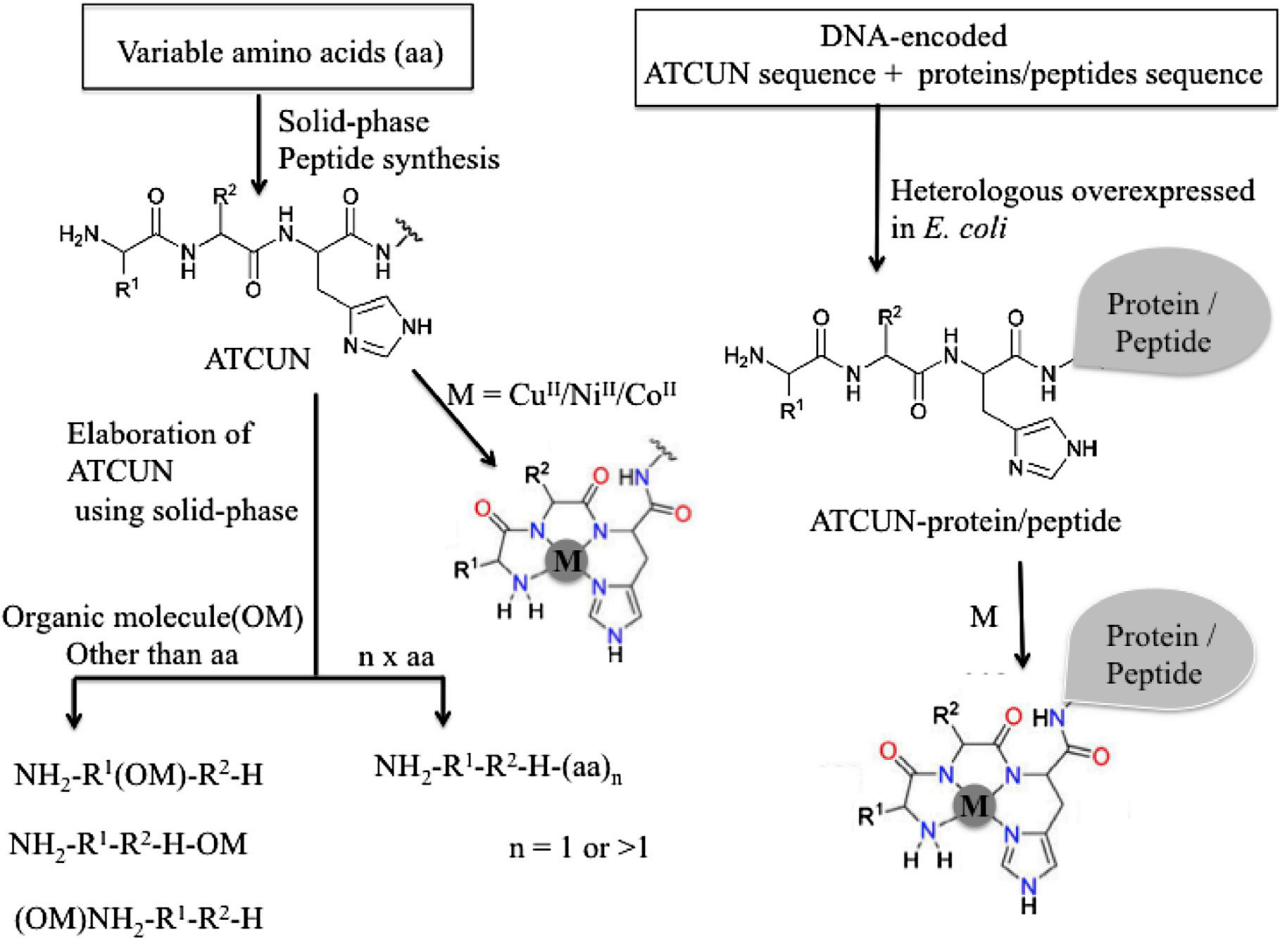
Schematic Representation of Design Strategies of Synthesized ATCUN and Its Metal Derivatives R^1^ and R^2^; variable amino acids at first and second positions in NH_2_-R^1^-R^2^-H sequence, H; histidine.

Artificial ATCUN motifs have been devoted considerable attention ever since its discovery in HSA.^1^ Decades of investigations have been put forward several interesting ATCUN derivatives, which have been designed purposefully in order to understand the stability, redox chemistry, efficacy, and selectivity. The basic design strategies of ATCUN derivatives are introduction of variable amino acid (X) at first and second positions in NH_2_-X-X-His sequence and/or conjugation of ATCUN motif with protein, peptide, or fluorophore (Figure 1). The initial design of ATCUN motif was interested from NH_2_-terminal protein sequences of albumins (Harford and Sarkar, 1997; Sankararamakrishnan et al., 2005; Lau et al., 1974), and antimicrobial peptides (AMPs) (Alexander et al., 2018, 2019). Variable side chains of amino acids surrounding His residue in ATCUN site significantly influence the activity and metal binding affinity, but they do not directly involve in metal coordination. This design is of interest, specifically, for catalytic activities like DNA, RNA, protein cleavage/modification as described below (Harford and Sarkar, 1997; Mack and Dervan, 1992; Jin and Cowan, 2005, 2007; Agbale et al., 2016; Yu and Cowan, 2017a, 2017b; Gonzalez et al., 2018).

A series of ATCUN motifs are synthesized by introduction of various amino acid residues at first and second positions of ATCUN, such as case-I: simplest amino acid (Gly), case-II: hydrophobic and aromatic amino acids (Phe and Tyr); case-III: hydrophilic and neutral amino acids (such as; Asn and Thr), case-IV hydrophilic and negatively charged amino acid (Val, Asp), case-V: hydrophilic and positively charged amino acids (Arg and Lys) and case-VI: L-to D-amino acid (Jin and Cowan, 2005, 2007; Miyamoto et al., 2016; Kozłowski et al., 1999; Sóvágó and Osza, 2006; Sóvágó et al., 2016; Bal et al., 1996). Generally, these simple and small peptides based ATCUN derivatives are synthesized by a conventional solid-phase peptide synthesis method employing the Fmoc strategy (Neupane et al., 2013).

Dervan et al. first designed ATCUN motif possessing GGH sequence that was the simplest replica of naturally occurring ATCUN motif in proteins or peptides (Mack and Dervan, 1990, 1992; Mack et al., 1988). In this regard, varieties of ATCUN derivatives have been designed. For example, the positively charged amino acid, like lysine (K) and arginine (R) at positions 1 and 2 in ATCUN motif enhances the Cu^II^ binding affinity, whereas negatively charged amino acid, like aspartic acid (D), glutamic acid (E) suppress the Cu^II^ binding affinity (Kozłowski et al., 1999). Moreover, the bulkiness and hydrophobic side chain of amino acid residues at first and second positions may increase the stability of Cu-ATCUN derivatives (Kozłowski et al., 1999; Bal et al., 1996; Sokolowska et al., 2002; Pettit et al., 1990). The stability of the various synthetic Cu^II^-ATCUN derivatives is tested under aqueous solution, blood plasma, and living animals (Miyamoto et al., 2016). In aqueous solution, the stability of Cu^II^-ATCUN derivatives is influenced by basicity of side chain at first and second positions, as the result of the competition between the protonation and the Cu^II^ coordination of the 4N ligands. In plasma, the stability of the ^64^Cu^II^–ATCUN derivatives is significantly increased by introduction of bulky and hydrophobic side chain of amino acid at first and second positions (Kozłowski et al., 1999; Camerman et al., 1976). In addition, incorporation of D-amino acid instead of L-amino acid elevates the stability and reactivity of ATCUN motif significantly (Jin and Cowan, 2005, 2007).

The above designed ATCUN derivatives are also conjugated with small organic molecules at positions 1, 2, 3 and NH_2_- (in N-terminus site) with or without a linker moiety. This designed has been, particularly, developed as a sensor (Yu and Cowan, 2017a, 2017b; Gonzalez et al., 2018; Torrado et al., 1998; Wendea and Kulak, 2015; Libardo et al., 2014). Torrado *et al.* have designed three ATCUN-organic derivatives where organic molecule, 5-(dimethylamino)naphthalene-1-sulfonamide (Dns), as a fluorophore has been incorporated into NH_2_-GGH at NH_2_- site with a linker (-CH_2_-, -C_2_H_4_- and -C_3_H_6_-) (Torrado et al., 1998). Several ATCUN-dns derivatives are reported where Dns group is attached to the vicinity of the ATCUN motif at any amino acid residue (at position, 1, 2, or 3) in ATCUN (Zheng et al., 2002; Choi et al., 2015).

Another strategy is the conjugation of ATCUN motif with protein and/or peptide that is generally synthesized genetically. Usually, a DNA fragment-encoded ATCUN coupled with protein or peptide sequence is amplified by polymerization chain reaction. Afterward, it is ligated into the vector for ATCUN-protein or -peptide expression. ATCUN-fusion proteins or peptides are generally obtained from heterologous overexpression in *E. coli* (Donaldson et al., 2001; Choi et al., 2015; Pauleta et al., 2007)*.* Mack et al. successfully introduced artificial ATCUN tag into NH_2_-terminus of the DNA binding Hin recombinase protein genetically (Mack and Dervan, 1990). Based on this concept, several hybrid ATCUN-protein (or peptides) derivatives are reported, which include multi-domain human ubiquitin protein (Donaldson et al., 2001), orange protein (ORP) (Maiti et al., 2017b), green fluorescent protein (GFP) (Choi et al., 2015), and AMPs (Alexander et al., 2018). In addition, the redox catalytic M-ATCUN domain is linked with a gene-specific recognition domain (peptide or substrate) that selectively targets the disease-associated gene, like DNA, RNA, and protein, leading to oxidative damage/modification.

Over the last few decades, the designed strategies have been implicated as a potential catalytic metallodrug toward nucleases and proteins inactivation as described below. The stability of M-ATCUN derivatives, *in vivo*, is also a key factor for clinical diagnosis because it must reach to target site with its active form (Mjos and Orvig, 2014). Overall, design of ATCUN derivative has been developed for wide applicability either as spectroscopic probe or as catalytic machine for nucleases, modification of biomolecules, and small molecule activation.

## Interaction of ATCUN Motif with Metal Ions

The ATCUN motif, a promising metal binding scaffold, binds with metal ions in a specific manner, defining four nitrogens (terminal amine, two intervening peptide bond nitrogen atoms and the histidine N3 nitrogen) coordinated square planar complexes. It is common to know that ATCUN motif interacts mainly Cu^II^ and Ni^II^ ions. In fact, it can interact with many metal ions, and this interaction depends on the metal ions, deprotonation of the amide groups, and coordination of histidine nitrogen (Burger, 1990). Deprotonation of amide nitrogen in aqueous medium requires strongly basic conditions (pKa ≈15) in the absence of metal ion. But the process is significantly enhanced in the presence of metal ions, when the imidazole ring of the histidine residue is available to act as an anchor for the metal ion (Bortolus et al., 2010; Sundberg and Martin, 1974). Only few metal ions are able to induce the deprotonation of CONH groups, forming M-ATCUN species at physiological pH. Among them, Cu^II^ and Ni^II^-ATCUN are formed at physiological pH, but Co^II^-ATCUN is formed at slightly higher pH (∼9) (Neupane et al., 2013; Hawkins and Martin, 1983). ATCUN motifs have been also shown to bind other metal ions, which are Au^III^, Pt^II^, and Pd^II^ at very low pH (∼1–2) to form square planar complexes as Ni-, Cu-ATCUN complexes (Best et al., 1997; Kirvan and Margerum, 1985; Lihi et al., 2017). These findings provide a useful information for the synthesis of new M-ATCUN derivatives.

Since HSA is known as the first source of ATCUN motif and participates in the Cu^II^ transport in blood (Harford and Sarkar, 1997; Laussac and Sarkar, 1984; Carter and Ho, 1994), the understanding of metal-albumin interaction is necessary to be considered in this respect. HSA possesses two specific Cu^II^ binding sites, ATCUN site, and the multimetal binding site (MBS), in which Cu^II^ binds strongly with ATCUN site over MBS (Bal et al., 2013; Al-Harthi et al., 2019). ATCUN motif in albumin not only interacts Cu^II^ or Ni^II^, but also interacts other metal ions, which are Zn^II^, V^IV^O, and Cd^II^ (Bal et al., 2013). Based on competition studies, V^IV^O prefers ATCUN site, while Zn^II^ or Cd^II^ prefers MBS (Al-Harthi et al., 2019).

The stability constant of Cu-ATCUN in HSA and other proteins/peptides is in range, 12 < log ^c^K_7.4_ < 15, depending on surrounding amino acid residues of ATCUN (Gonzalez et al., 2018; Rozga et al., 2007). In addition, ATCUN motif shows higher selectivity for Cu^II^ binding over other physiological cations because Cu^II^ may has strong lewis acid character and preferes square-planar geometry. In general, the binding affinity of the ATCUN motif for Cu^II^ is higher (about six order of magnitude) than for Ni^II^.^65^ The binding affinity of some synthetic as well as naturally occuring ATCUNs are tabulated in Table 1 (Gonzalez et al., 2018; Rozga et al., 2007).

**Table 1:** Stability Constant/Binding Affinity of Cu-ATCUN from Variable Sources

Natural Protein/Peptide and Synthetic ATCUN Motif	Cu^II^ Binding Sequence	Stability Constant Log ^c^K_7.4_	Binding Affinity (K in M^−1^)
HAS (Gonzalez et al., 2018; Rozga et al., 2007)	DAH	12.0	1.0 x 10^12^
BSA (Rozga et al., 2007)	DTH	12.0	1.0 x 10^12^
Hepcidin N-term (Płonka and Bal, 2017)	DTH	14.7	5.0 x 10^14^
des-angiotensinogen N-term (Sokolowska et al., 2002)	VIH	13.0	1.0 x 10^13^
Human copper transporter (hCTR1-14aa) (Bossak et al., 2018)	MDH	11.0	1.0 × 10^11^
Aβ_4-16_ (Mital et al., 2015)	RFH	13.5	3.2 x 10^13^
HP2 N-term (Bal et al., 1997)	RTH	14.5	3.2 x 10^14^
Endostain N-term (Kolozsi et al., 2009)	HSH	14.5	3.2 x 10^14^
GGH-COOH (Hay et al., 1993)	GGH	12.4	2.5 x 10^12^
DAHK-NH_2_ (Rozga et al., 2007)	DAH	13.8	6.3 x 10^13^
YYH-COOH (Miyamoto et al., 2016)	YYH	14.4	2.5 x 10^14^
MDH-NH_2_ (Bossak et al., 2018)	MDH	13.1	1.3 × 10^13^
MNH-NH_2_ (Bossak et al., 2018)	MNH	14.5	3.2 × 10^14^

## Redox Chemistry of M-ATCUN Derivatives

The redox chemistry of M-ATCUN derivatives is a useful index due to its wide variety of catalytic activities. So, intense interests in electrochemical studies on M-ATCUN derivatives are described in literature (Gonzalez et al., 2018; Neupane et al., 2013; Mital et al., 2015; Hureau et al., 2011; Wiloch et al., 2017). The redox potential of M^n+^/M^(n−1)+^ in M-ATCUN derivatives highly depends on the nature of amino acid at periphery of ATCUN motif and geometry of metal-complexes. For instance, copper exhibits mainly two accessible redox couples, Cu^II^/Cu^I^ and Cu^III^/Cu^II^ in various catalytic activities. The Cu^II^/Cu^I^ is more available in biological system over Cu^III^/Cu^II^ redox couple (Solomon et al., 2014). The inherent problem between Cu^I^ and Cu^II^ is in coordination chemistry (Solomon et al., 2014; Maiti et al., 2018). For instance, Cu^II^ (Cu^III^ also) adopts a square planar geometry, but Cu^I^ cannot adopt the same geometry as Cu^II^ and it prefers tetrahedral or tri-, bi-coordinated geometry (Solomon et al., 2014; Maiti et al., 2018). Thus, Cu^II^/Cu^I^ redox couple is highly geometric reorganization, resulting less efficient in redox chemistry, whereas the Cu^III^/Cu^II^ redox couple involves low reorganization energy, resulting more efficient in redox chemistry. In Cu-ATCUN derivatives, the key point is the Cu^II^/Cu^I^ redox couple, where Cu^I^ cannot bind at the same coordination site as Cu^II^.

The Ni-ATCUN analogs, generally, involve Ni^II^/Ni^III^ redox couple in their potential catalytic applications. The transformation from Ni^II^ (Sq. planar) to Ni^III^ (sq. pyramid) involves geometry organization accompanying large gain in ligand field stabilization energy with respect to Cu^II^/Cu^III^ redox couple in Cu-ATCUN system (Bossu et al., 1977). The other analog, Co-ATCUN derivative, generally, shows Co^II^/Co^III^ redox couple in their redox chemistry without geometry organization (both are sq. planar geometry). Such electrochemical studies are performed on variety of designed linear and cyclic ATCUN motifs (Jin and Cowan, 2005, 2007; Neupane et al., 2013, 2014; Mital et al., 2015; Hureau et al., 2011). The redox chemistry of M-ATCUN derivative is influenced by several factors, such as geometry, stability of M^III^ center, variable amino acid, positioning of amino acid in ATCUN motif, and stereochemical orientation of amino acid residues relative to the M-ATCUN equatorial plane.

In this regard, Cowan et al. extensively studied on electrochemistry of a series of designed linear M-ATCUN derivatives (Table 2) (Jin and Cowan, 2007). Both Cu- and Ni-ATCUN derivatives show decrease in their redox potential with an increasing number of Lys residues in motif that influence the enhancement of σ-donor character relative to that of Gly on the stabilization of the M^III^ center (Jin and Cowan, 2007). The redox potential is also influenced by the position of Lys in ATCUN derivatives. At first position, Lys residue has been greatly extended to stabilize the Cu^III^ oxidation state than second position. The Lys residue at fourth position in Cu-GGHK, does not influence the Cu^III/II^ redox potential as seen in Cu-KGH derivative because backbone amide of fourth position Lys does not directly bind to Cu center. In Ni-KGHK derivative, the side chain amine groups of Lys residues (at second and fourth positions) interact at the axial site of Ni^III^ center, leading to more stabilization of Ni^III^. Similarly, incorporation of two Lys at first and second positions in Ni-KKH sequence, the Ni^III^ is effectively stabilized relative to the first or second position alone (Bossu et al., 1977; Murray and Margerum, 1982). So, stabilization of Cu^III^ and Ni^III^ in ATCUN derivatives depends not only on electrostatic interaction but also on the spatial orientation of the amino acid residues. In addition, the redox potential of Cu^III/II^-GGH-COOH is increased to ∼34 mV upon amidation at remote C-terminal COOH. Similar trend is observed in redox potential of Ni^III/II^-GGH-CONH_2_ (∼80 mV with respect to free carboxylate in GGH-COOH) (Jin and Cowan, 2007). Further study with other derivative, KGHK-COOH, upon amidation of the more remote C-terminus, the redox potential is noticeable increased to ∼0.9 mV for Cu^III/II^-KGHK and ∼20 mV for Ni^III^-KGHK. The switching from L- to D-Lys, the Cu^III^ is more stabilized, due to specific spatial orientation of D-Lys that leads to more negative redox potential corresponding to L-Lys isomers of Cu-ATCUN derivatives (Table 2). This redox effect is also observed in D-isomer, Ni^III^-kkH, but it is significantly lower extent to redox potential (Jin and Cowan, 2007).

**Table 2:** Redox Potential of Cu^II^-ATCUN and Ni^II^-ATCUN Derivatives

Cu-ATCUN^a^	E = Cu^III^/Cu^II^ (ΔE = Relative to Cu^II^-GGH-CONH_2_) (mV vs. NHE)	Ni-ATCUN^a^	E = Ni^III^/Ni^II^ (ΔE = Relative to Ni^II^-GGH-CONH_2_) (mV vs. NHE)
Cu^II^-GGH-CONH_2_	1068 (00)	NiII-GGH-CONH2	1087 (00)
Cu^II^-GKH-CONH_2_	1057 (−11)	NiII-GKH-CONH2	1097 (10)
Cu^II^-KGH-CONH_2_	1051 (−17)	NiII-KGH-CONH2	1097 (10)
Cu^II^-KKH-CONH_2_	1017 (−51)	NiII-KKH-CONH2	1077 (−10)
Cu^II^-KGHK-CONH_2_	1049 (−19)	NiII-KGHK-CONH2	1067 (20)
Cu^II^-GkH-CONH_2_	1032 (−36)	NiII-GkH-CONH2	1157 (18)
Cu^II^-kGH-CONH_2_	1033 (−35)	NiII-kGH-CONH2	1067 (−20)
Cu^II^-kkH-CONH_2_	993 (−69)	NiII-kkH-CONH2	1047 (−40)
Cu^II^-GGH-COOH	1034 (−34)	NiII-GGH-COOH	1007 (−80)

a CV is measured in 25 mM phosphate buffer, pH 7.4 and 0.1 M KCl. (Lower case letters represent D-amino acid).

Dervan and coworkers first showed that designed GGHG-Hin-recombinase derivative performed DNA cleavage oxidatively (Mack et al., 1988). Therefore, Cu-GGHG derivative is of considerable interest for the study of redox chemistry (McDonald et al., 1997; Green et al., 2004). The Cu^II^-GGHG-COOH shows the redox potential at 978 mV vs. NHE, which is very close to Cu^II^-GGH-COOH system (∼1003 mV vs. NHE) (McDonald et al., 1997). Another important naturally occurring ATCUN sequence, DAHK is found in the blood plasma, HSA (Hureau et al., 2011; Sendzik et al., 2017). The CV trace of designed Cu^II^-DAHK derivative shows a reversible peak at E_1/2_ = 0.77 V (vs. AgCl/Ag) corresponding to the Cu^III^/Cu^II^ redox couple, but no reduction redox couple, Cu^II^/Cu^I^ is observed (Hureau et al., 2011; Guilloreau et al., 2007). Therefore, the reduction of Cu^II^ to Cu^I^ in model peptide, Cu^II^-DAHK is difficult by ascorbate, but it is relatively easier in HSA (Hureau et al., 2011). This unusual redox behavior of Cu-DAHK in serum albumin is assigned to the presence of two close proximity His residues near-by ATCUN motif that facilitates the reduction of Cu^II^ to Cu^I^ by ascorbate and stabilize it after reduction via His-Cu^I^-His coordination (Haas et al., 2011; Wezynfeld et al., 2016; Hureau, 2012). The bis-His motif has higher magnitude for Cu^I^ binding over ATCUN motif (Zheng et al., 2002; Płonka and Bal, 2017). Therefore, ATCUN motif and near by bis-His motif both significantly play the redox interconversion between Cu^II^ and Cu^I^ (Haas et al., 2011; Wezynfeld et al., 2016; Hureau, 2012).

The redox chemistry is also examined in a series of designed cyclic and linear M-ATCUN derivatives (Neupane et al., 2013). Joshua Kritzer et al. reported a nice work on redox chemistry of cyclic and linear ATCUN derivatives, including effects of macrocyclization (Neupane et al., 2013). The copper bound cyclic- and linear-ATCUN derivatives show quasi-reversible peak with almost the same reduction potential value of Cu^III^/Cu^II^ redox couple (740–810 mV vs. SCE) and no reduction couple, Cu^II^/Cu^I^ is observed. In addition, the cyclic Cu-ATCUN derivatives enhance the Cu^III^/Cu^II^ redox cycling (facilitates electron transfer) than linear Cu-ATCUN derivatives (Neupane et al., 2013).

Like Cu- and Ni-ATCUN, Co-ATCUN analogs, including Co-GGH and Co-KGHK derivatives show the redox interplay mainly between Co^III^ and Co^II^, with relatively low redox potentials values at −119 and −228 mV vs. NHE, respectively (Joyner and Cowan, 2011). This result also supports that upon inclusion of positive charge amino acid (Lys) in ATCUN site, the redox potential is decreased.

## Production of Hydroxyl Radical by M-ATCUN Derivatives

The understanding of mechanism and efficiency of hydroxyl radical formation by M-ATCUN are an important task because the oxidative cleavage/modification of biomolecules are induced by the hydroxyl radical (⋅OH) (see below). The formation of ⋅OH is directly associated with redox potential of M-ATCUN derivatives (Jin and Cowan, 2007; Gokhale et al., 2008; Joyner et al., 2011; Fang et al., 2004). Therefore, the ⋅OH production ability of varieties Cu-ATCUN derivatives is examined in presence of H_2_O_2_ (oxidant) and ascorbate (reductant) (Jin and Cowan, 2007; Neupane et al., 2013; Joyner and Cowan, 2011). Several experimental observations conclude that the production of ⋅OH by Cu-ATCUN is more efficient in presence of H_2_O_2_/ascorbate system with respect to H_2_O_2_ or ascorbate alone (Jin and Cowan, 2007; Neupane et al., 2013; Libardo et al., 2014; Joyner et al., 2011; Santoro et al., 2018). For instance, a series of tri-peptide Cu-ATCUN derivatives (DAH, DMH, DSH, DTH, EAH, GGH, GKH, GNH, LKH, NGH, RTH, SMH, and VIH) are designed for testing the ⋅OH production ability in presence of H_2_O_2_ and ascorbate. Among them, Cu–GGH shows the highest rate (52.54 ± 0.76 μM min^−1^), whereas EAH shows the lowest rates (1.14 ± 0.04 μM min^−1^) of ⋅OH production in presence of H_2_O_2_/ascorbate (Asc^−^) system (Libardo et al., 2014). The tri-peptide system is expanded to tetra-peptide, such as Cu^II^-KGHK that has been also examined for the production of ⋅OH in presence of Asc^−^/H_2_O_2_ and the production rate of it is compared with Cu-GGH. Both Cu-peptides derivatives show the higher rate of ascorbate consumption in presence of H_2_O_2_ (turn over number >100 for GGH and 44 ± 7 for KGHK) than absence of H_2_O_2_ (turn over number 17 ± 2 for GGH and 11 ± 2 for KGHK) (Jin and Cowan, 2007; Joyner and Cowan, 2011).

Above results conclude that redox dependent mechanism of HO⋅ formation by Cu-ATCUN derivatives is involved in the variable oxidation states (I, II, and III) of copper. At neutral pH, the reduction potential of H_2_O_2_/HO⋅, and ascorbyl radical/ascorbate are 380 mV (vs. NHE), and −66 mV (vs. NHE), respectively (Wood 1988; Borsook and Keighley, 1933). Therefore, H_2_O_2_/ascorbate system plays the redox chemistry with M-chelates complexes within +380 to −66 mV reduction potential window. In presence of ascorbate, the generation of ⋅OH by Cu^II^-ATCUN derivatives is proposed through a Cu^II^/Cu^I^ reduction couple, but the high reduction potential value of Cu-GGH (1038 mV vs. NHE) or Cu-KGHK (1058 mV vs. NHE) is incompatible with reduction of Cu^II^-ATCUN derivatives by ascorbate (Jin and Cowan, 2007; Santoro et al., 2018). In this case, the key point is geometry reorganization in Cu^II^/Cu^I^ redox couple, where Cu^I^ could not bind at the same coordination site as Cu^II^. So, Cu^I^ may be a free state or weakly coordinated ATCUN motif, and thereby Cu^I^ likely binds with biomolecules *in vivo*. This hypothesis is tested on Cu^II^-KGHK, Cu^II^-DAHK, or Cu^II^-FRHD derivatives in presence of Cu^I^ chelating agent, bathocuproinedisulfonate (BCS). Upon addition of ascorbate or H_2_O_2_, the formation of Cu^I^-BCS_2_ complex is increased and consequently the production of ⋅OH is decreased or completely seized (Santoro et al., 2018). This observation indicates that the formation of Cu^I^ is directly connected with the formation of ⋅OH. Still, it has a space to improve the redox mechanism.

Similarly, Cu-analogs, including Ni-ATCUN and Co-ATCUN derivatives, are often studied for the production of ⋅OH. Ni-ATCUN shows mainly one accessible redox couple, Ni^III^/Ni^II^, which significantly generates ⋅OH in presence of H_2_O_2_/ascorbate system. Both Ni-GGH and Ni-KGHK derivatives show the higher rate of ascorbate consumption in presence of H_2_O_2_ (turn over number 40 ± 10 for GGH and 30 ± 8 for KGHK) than absence of H_2_O_2_ (turn over number 9 ± 2 for GGH and 9 ± 2 for KGHK) (Jin and Cowan, 2007; Joyner and Cowan, 2011). Unlike Ni- and Cu-ATCUN derivatives, both Co-GGH and Co-KGHK derivatives show the same turn over number (>100) of ascorbate consumption, even in absence of H_2_O_2_, due to lower redox potential of Co^III^/Co^II^ couple that facilitates ⋅OH production (Joyner and Cowan, 2011).

## Applications of M-ATCUN Derivatives

**Figure 2:**
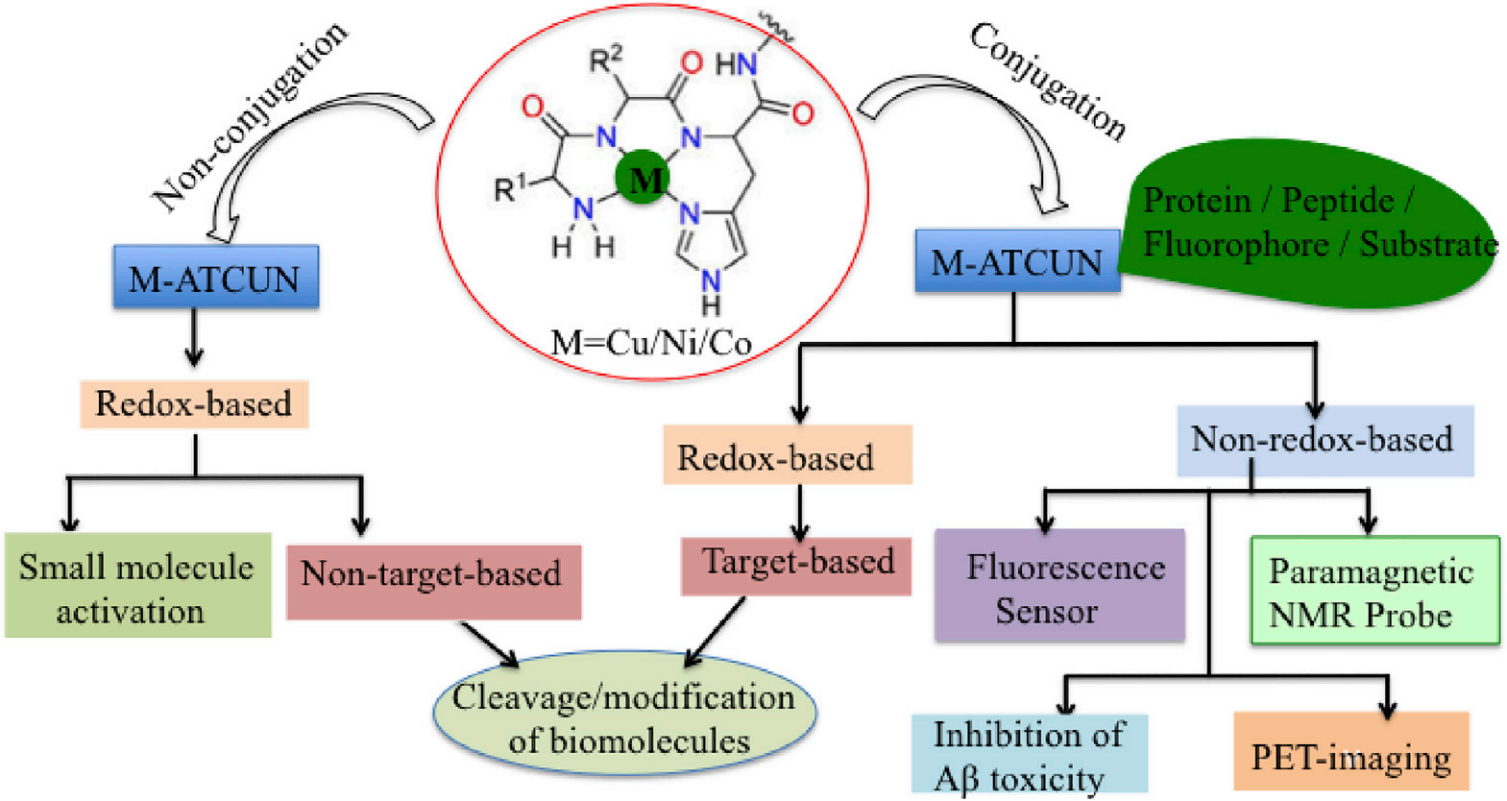
Schematic Representations of Designed M-ATCUN Derivatives in Varous Applications

In this context, a wide range of applications of M-ATCUN derivatives can be classified into two classes, – (1) redox-based and (2) non-redox-based applications (Figure 2). M-ATCUN, especially, Cu-ATCUN shows a redox silent component in presence of biological reductant, ascorbate due to its high redox potential (ranging from 0.87 to 1.07 V vs. NHE)^7^ and inherent geometry problem between Cu^II^ and Cu^I^, it make a redox silent component in presence of biological reductant, ascorbate. Interestingly, Cu-ATCUN derivatives have shown to ability the production of HO⋅ under H_2_O_2_/AcsH^−^. Therefore, Cu-ATCUN derivatives can be utilized for various applications into two ways: presence of H_2_O_2_/AcsH^−^ (redox active) and absence of H_2_O_2_/AcsH^−^ (redox inactive). Based on literature survey, Cu/Ni-ATCUN derivatives are largely exploited toward redox-based applications, whereas Co-ATCUN derivatives are limited. Same redox couple of copper is more intensely used as a sensor probe in fluorescence turn-on/off switch. As a result, copper has received a considerable amount of attention in this field. In this review, we focus on Cu-, Ni-, and Co-based designed metal-ATCUN derivatives and their applications toward non-redox- and redox-based.

### Non-Redox-Based Applications

The transition metal ions are extensively used for redox chemistry in a variety of chemical and biological transformations, but their specific stable oxidation state has been also used as a potential spectroscopic probe due to its inherent spectroscopic properties. Metal ions possessing observable spectroscopic features are used as probes in specific techniques including UV-vis, NMR, electron paramagnetic resonance (EPR), Mössbauer, fluorescence quencher and extended X-ray absorption fine structure in many metalloproteins (Maiti et al., 2017a; Hagen 2006; Holm et al., 1996). For example, Cu^II^ is a paramagnetic species whereas Cu^I^ is a diamagnetic species. Based on this spectroscopic advantage, Cu^II^-ATCUN state has been studied extensively as a paramagnetic probes and sensors (Donaldson et al., 2001; Maiti et al., 2017b; Deng et al., 2019a, 2019b; Torrado et al., 1998). Significantly, this review is mainly focused on stable Cu^II^-ATCUN derivatives as spectroscopic probe and positron emission tomography (PET) imaging (Miyamoto et al., 2016). In addition, the ATCUN motif acts as a Cu-chelator for amyloid-β (non-redox-based application) (Zhang et al., 2016).

#### Spectroscopic Probes

Spectroscopic probes have been extensively investigated and widely used in many fields due to their powerful capability of enlightening and elucidating structural and/or functional features of small/macro-molecules. Several spectroscopic tools are available in literature. However, only a few specific, efficient, and less toxic designed small metallo-peptide-based probes are available in literature. Nature provides a small ATCUN motif that represents a promising spectroscopic toolbox due to its small size, causing minimal perturbation in protein structure and function. The utility of ATCUN as a spectroscopic probe is studied by artificially introducing it into protein or peptide. This motif binds, specifically, paramagnetic transition metal ions like Cu^II^ ion to originate a Cu^II^-ATCUN derivative that is useful for paramagnetic NMR study (Donaldson et al., 2001; Mal et al., 2002; Gaponenko et al., 2000; Brath et al., 2015). This NMR study can provide valuable information on long-distance interactions between metal ions and surrounding groups. This probe is also widely used as a universal fluorescence quencher that is highly suitable for various pharmaceutical and biomedical applications as discussed below.

##### Paramagnetic NMR Reporter

**Figure 3:**
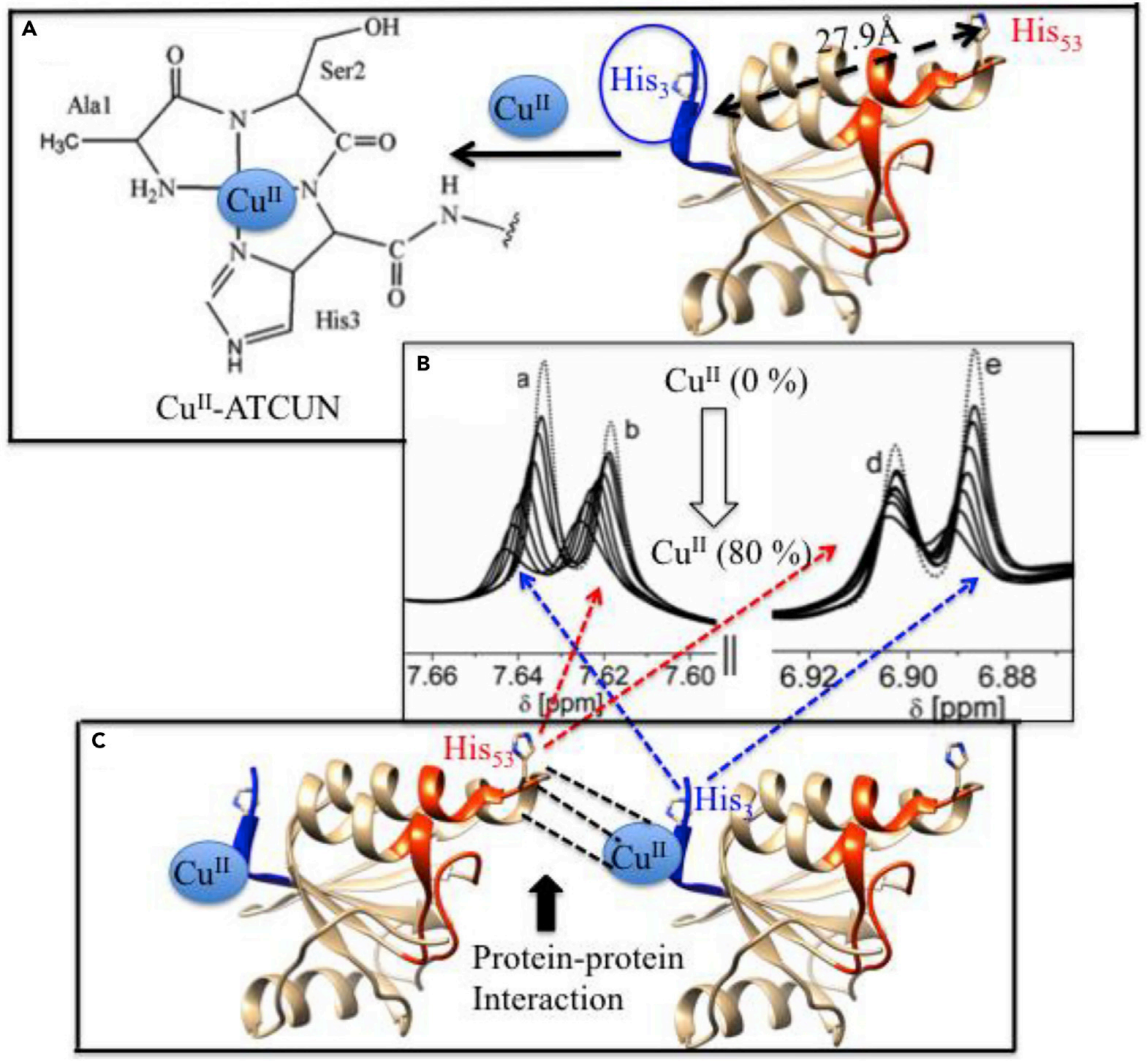
Application of Cu-ATCUNs as Paramagnetic NMR Reporters (A–C) (A) The paramagnetic NMR probe, Cu^II^-ATCUN is attached at N-terminus of ORP. The crystal structure of apo-ATCUN-ORP is derived from PDB 2WFB (Najmudin et al., 2009), (B) Paramagnetic ^1^H-NMR spectrum (600 MHz) of apo-ATCUN-ORP is titrated with Cu^II^ salt (0–80%) in mixture of 50 mM Tris-HCl at pH 7.6/D_2_O (80/20%) and only His_53_ (b and d peaks) and His_3_ (a and e peaks) amino acid redidues are represented those are highly affected, and (C) Schematically shows the protein-protein interaction in head-tail fashion (dotted black lines). Modified from , Maiti et al., 2017b.

Paramagnetic-NMR has been acknowledged as a powerful tool for the study of biomolecules when the first time this tool successfully solved the solution structure of metalloproteins (Banci et al., 1994). Paramagnetic-NMR is well established (Clore and Iwahara, 2009), and its probe is widely used to obtain valuable information about the structure and function of biomolecules (Otting 2010). Those proteins in which paramagnetic transition metal ions are already present or the one in which diamagnetic transition metal ions are replaced, can be used as a paramagnetic probe for protein structural studies, including protein-protein interaction or protein-nucleic acid interfaces in folding and unfolding proteins (Cerofolini et al., 2018; Piccioli and Turano, 2015; Balayssac et al., 2008). For other proteins, paramagnetic metal binding scaffold, which is absent, can be introduced either by genetic engineering (Barthelmes et al., 2011) or chemically covalent attachment with wanted proteins or peptides. The short, three-residues Cu^II^-binding ATCUN motif acts as a paramagnetic NMR probe where Cu^II^ ion shows slow electron spin relaxation that causes broadening of proton NMR resonances (Clore and Iwahara, 2009; Viles et al., 1999; Kalverda et al., 1996). Donaldson et al. first successfully genetically introduced artificial ATCUN tag into multi-domain human ubiquitin protein (Phillips et al., 2001; Huang et al., 1999). Cu^II^-ATCUN-ubiquitin, a paramagnetic probe, is a suitable candidate for extracting long-range distance restraints from NMR study, which is useful for structure refinement (Donaldson et al., 2001). In this regard, Mal et al. had also designed a Cu^II^-ATCUN-protein model system (Maiti et al., 2017b), where Cu^II^-ATCUN domain acts as an NMR-probe for understanding the interaction mechanism between protein calmodulin (CaM) and its target serine/threonine protein kinases (CaM kinases). It is a very useful NMR technique. Therefore, it is rapidly growing in a wide array of intermolecular interactions of biomolecules. Yu et al. employed Cu-GCH as a paramagnetic NMR probe that characterized protein-protein interactions (Yu et al., 2009). In 2017, Maiti et al. also reported a paramagnetic NMR probe, Cu^II^-ATCUN motif that was inserted into N-terminus of ORP for understanding the molybdenum/copper heterometallic cluster assembly in protein pocket (Maiti et al., 2017b). The ^1^H-NMR study clearly indicates that His_53_ is affected by Cu^II^-ATCUN probe, even though, it is out of paramagnetic region (the distance between His_53_ and His_3_ is ∼27.9 Å). It is only possible when two molecules interact with each other in head-to-tail fashion (Figure 3). This Cu^II^-ATCUN-ORP derivative provides a model of intermolecular protein-protein interaction (Maiti et al., 2017a, 2017b).

These paramagnetic studies are expected to get more benefit from the diversity of ATCUN-conjugated protein model system, and undoubtedly it is a valuable addition into the NMR toolbox for the characterizing of macromolecular structure-function relation.

##### Fluorescence Sensors

Fluorescence assay is one of the most important optical analytical tools and has a wide range of applications in the fields of chemistry, biology, clinical diagnosis, food industry, pharmaceutical chemistry, and environmental science because of its high sensitivity, rapid response, and quantitative yield (Fabbrizzi et al., 1996; Krämer 1998). Fluorescence probes are constructed by combination of two essential components, fluorophore (chromophore), and the quencher (sensor). Fortunately, naturally occurring Cu^II^ binding ATCUN motif acts as a fluorescence quencher due to its paramagnetic character. For example, ATCUN motif in BSA selectively binds Cu^II^ that significantly quenches the fluorescence emission (Tian et al., 2004; Kruck et al., 1976) by protein containing tryptophan residues (typical fluorescence signature of a protein) (Beechem and Brand, 1985). Therefore, this advantage of naturally occurring Cu-ATCUN motif has been employed as a chemo-sensors for detection and screening of certain cellular metal ions, catalytic redox reaction of DNA/RNA cleavage, protease, and bio-imaging.

Several fluorescence probes are designed where fluorophore, 5-(dimethylamino)naphthalene-1-sulfonamide (Dns) is attached to the vicinity of the receptor (Cu^II^-ATCUN motif) for detection of Cu ions as well as catalytic activity of Cu-ATCUN motif (Zheng et al., 2002). By applying this strategy, Torrado et al. first designed a series of ATCUN motifs (NH_2_-G(X_n_-Dns)GHG), where amino acid at first position of ATCUN motif was conjugated by dns with spacer (X = -CH_2_, and n = 1, 2 and 3). The spacer makes a distance between the fluorophore (Dns) and receptor (Cu^II^), where shorter spacer quenches fluorescence signal more efficiently (Torrado et al., 1998). Torrado et al. first successfully applied this method in order to understand the fate of copper in catalytic DNA/RNA cleavage (Torrado et al., 1998). To expand the utility of Dns fluorophore, Zhen et al. developed a fluorescent chemosensor (Dns-NH_2_-GGHG), where dansyl fluorophore was attached into N-terminal amino group, resulting Dns directly participating in the binding with Cu^II^. This model significantly quenches the emission of fluorophore compared to side branch labeling method (Zheng et al., 2002). Young et al. also compared Cu^II^ binding affinity in H(K^Dns^)HH with other three short peptides (HP(K^Dns^)DHDH, Ac-DH(K^Dns^)HD, and HP(K^Dns^)DHDH) by fluorescence quenching study (Young et al., 2015). The fluorescent-labeled (Dns) ATCUN probe is also used on CuO-NPs-based colorimetric immunoassay in clinical diagnosis for the detection of prostate-specific antigen (Deng et al., 2019a, 2019b).

This chemo-sensor has been also utilized toward catalytic activity of Cu-ATCUN motif. In this regard, Choi et al. designed a new fluorescence probe, where the fluorophore, GFP was added next to GGH sequence by genetically (Choi et al., 2015). Upon addition of Cu^II^, the fluorescence of GFP is quenched by about 85%. In 2015, Wende et al. redesigned a series of ATCUN conjugated fluorophore (R-NH_2_-Dap-β-Ala-His-Ser-Ser-CONH_2_; R = fluorophore = Rhodamine B, dansyl chloride and fluorescein isothiocyanate) derivatives that significantly cleaved DNA through oxidative pathway. The fate of metal ion (Cu^II^), during the redox process, was monitored by fluorescence studies (Wendea and Kulak, 2015).

**Figure 4:**
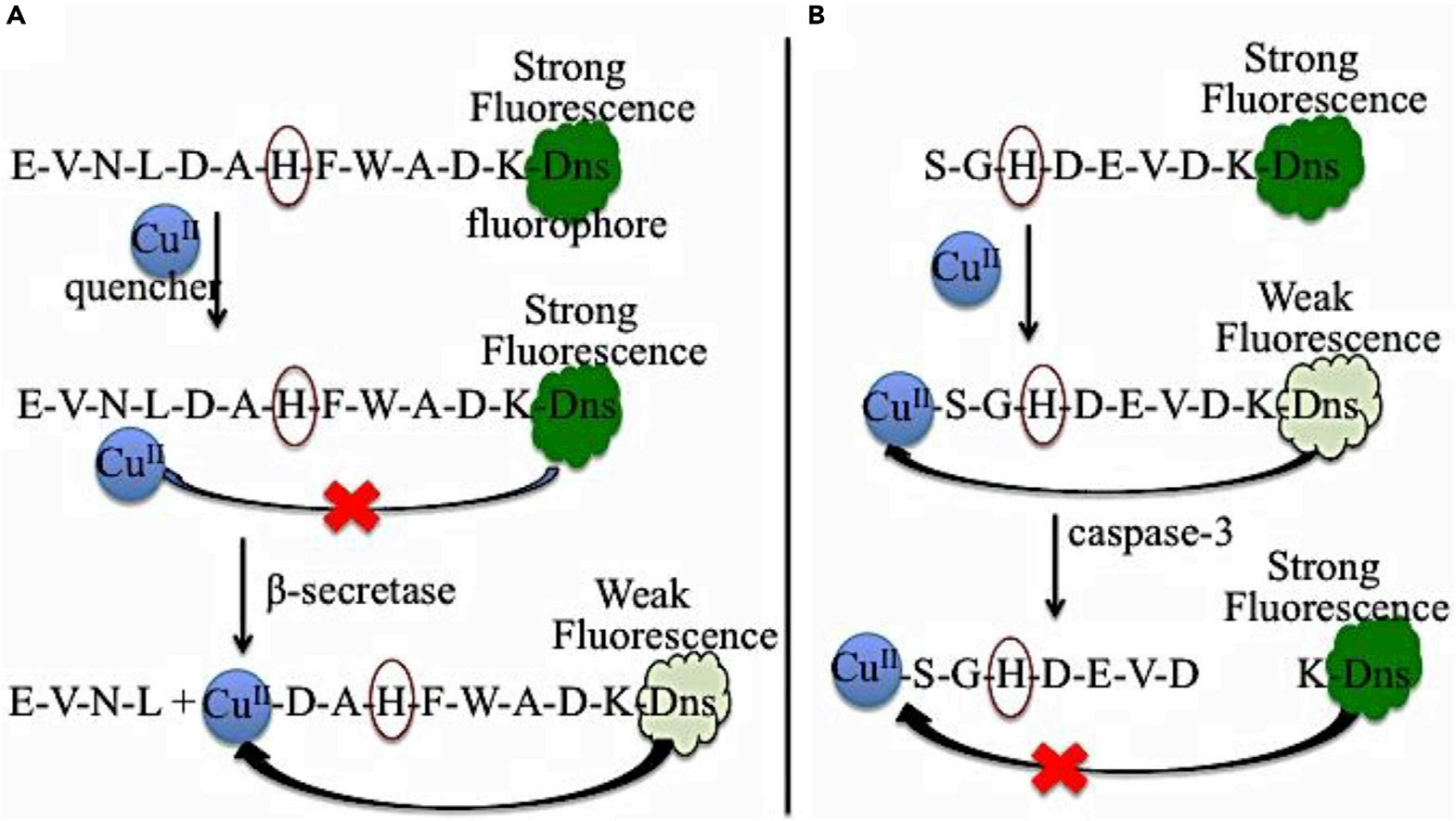
Application of ATCUNs as Fluorescent Reporters Fluorescence emission of proteases models, β-secretase (A) and caspase-3 (B) is quenched by Cu^II^-ATCUN. Dns; flurophore. Modified from Deng et al., 2019b.

Protease activity is also detected by fluorescence assay. In this assay, generally, the substrate peptide for protease is attached with a pair of fluorophore-quencher system. Deng et al. (Deng et al., 2018) reported three fluorophore labeled peptides including EVNLDAHFWADK-Dns, and DAHFWADK-Dns (or SGHDEVDK-Dns) as proteases model for β-secretase (as known as precursor of amyloid) (Haass and Selko, 2007) and caspase-3 (central mediator of cell apoptosis) (Thornberry et al., 1997; Boeneman et al., 2009), respectively. In this proteases assay, initially, almost no change in the fluorescence signal intensity is observed even upon addition of Cu^II^ in EVNLDAHFWADK-Dns but after cleavaging in-between L and D amino acid residues in EVNLDAHFWADK-Dns by β-secretase (Folk and Franz, 2010), Cu^II^ binds ATCUN motif (DAH) in fluorophore-labeled fragment (DAHFAWADK-Dns), that significantly reduces the fluorescence intensity (Figure 4). This result shows the inhibition of β-secretase activity. As a result, β-secretase can be used as a therapeutic target for AD (see next section). The other proteases model (caspase-3), SGHDEVDK-Dns with Cu^II^ exhibits poor fluorescence signal due to the presence of ATCUN motif (SGH) that strongly binds Cu^II^ as a quencher. After proteases by caspase-3, the fluorophore-labeled fragment (K-Dns) is separated from Cu^II^-ATCUN and shows good fluorescence signal. This result may be potentially useful as fluorescence imaging for detection of cell apoptosis.

In the living system, copper-homeostasis is a complex process. Prior to entry into cell, Cu is present mainly as Cu^II^ state and ensures copper homeostasis under physiological conditions by one of the most important proteins, albumin, which represents as labile Cu pool in human blood (Harford and Sarkar, 1997; Laussac and Sarkar, 1984). This labile copper pool has clinically significance as potential markers of copper-related pathologies (Lowe et al., 2017; Maiti and Moura, 2020). Therefore, measurement of this labile copper pool in biological samples is of considerable interest by fluorescence study. Quite recently, Falcone et al. (Falcone et al., 2020) have reported a turn-off luminescent sensor for Cu^II^, which combines ATCUN motif with long-lifetime luminophore, lanthanide (i.e. Tb^3+^), enabling Cu^II^ detection in biological-like media.

#### Inhibition of Amyloid-β Toxicity

Above section, Figure 4 shows that the model peptide is cleaved to generate ATCUN fragments by β-secretase. It is well known that the formation of amyloid β-peptide (Aβ) by β-secretase is considered as a key step for the AD (Deshpande et al., 2006). The extracellularly released Aβ has different isoforms, which include Aβ_1−40_, Aβ_1−42_, and N-truncated Aβ_4−42_ members. Among them, Aβ_4−42_ isoforms of Aβ family is high abundance in brains of healthy and AD human, representing a dominant species in AD (Portelius et al., 2010). Other than, Aβ_4−42_ has strong ability to bind Cu^II^, that not only induces Aβ aggregation into cytotoxicity elements but also generates reactive oxygen species (ROS), causing AD (Faller 2009; Jha et al., 2017; Young et al., 2014; Cheignon et al., 2017).

**Figure 5:**
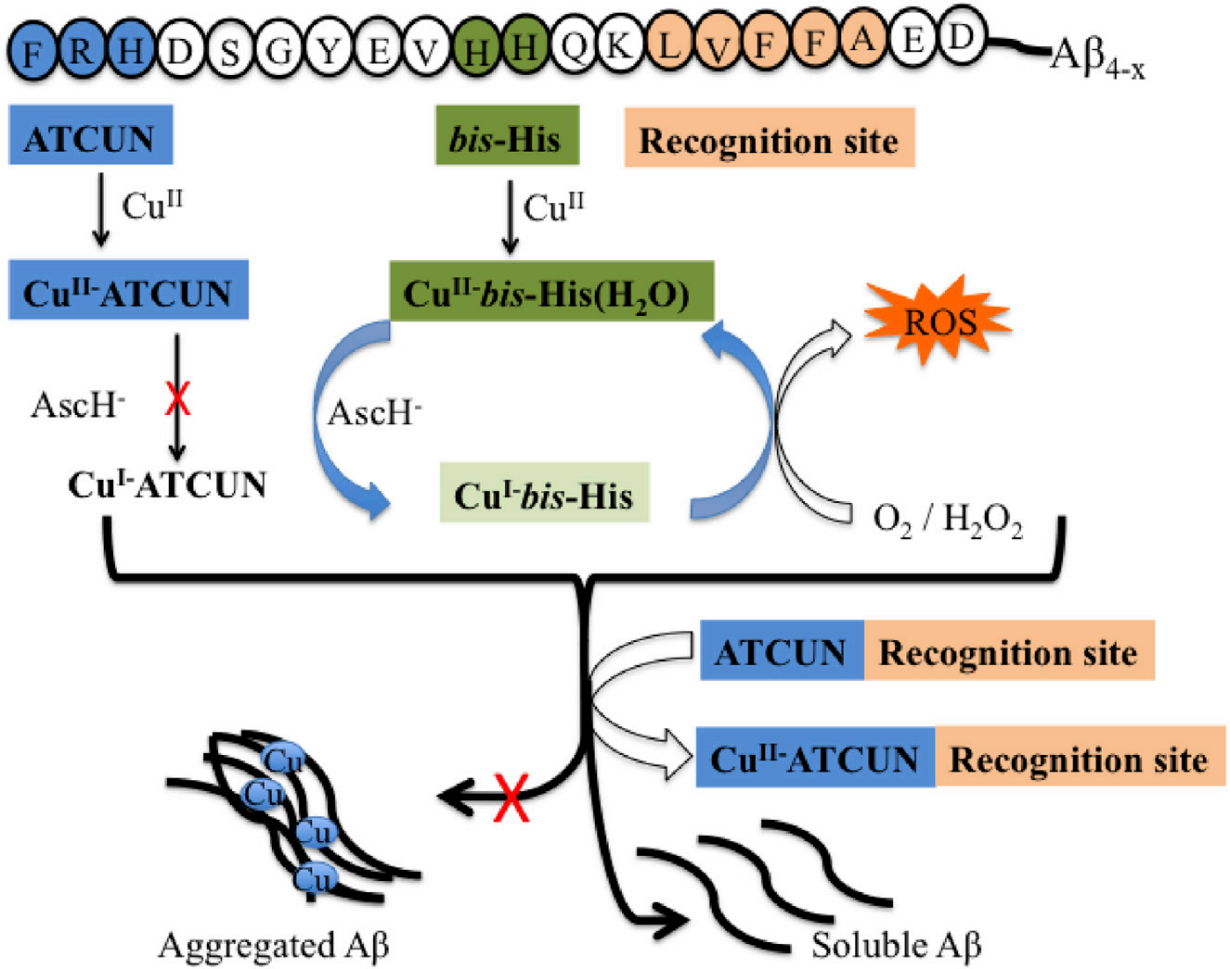
Aβ_4-x_ Represents the ATCUN (Blue), Bis-His (Green) and Recognition Sites (Orange) In presence of copper, Aβ can produce aggregated as well produce HO⋅ but upon addition of apo-ATCUN, Cu is trapped by ATCUN and inhibit the aggregation of Aβ.

Mital et al. reported that a model peptide, Aβ_4−16_ binds Cu^II^ with binding affinity, ^c^K_7.4_ of 3.4 x 10^13^ M^−1^, which is stronger than Aβ_1−x_ (∼10^10^ M^−1^) (Mital et al., 2015) but almost same in HSA (∼10^13^ M^−1^) (Bossak-Ahmad et al., 2020; Pushie et al., 2019). The electrochemical response of Cu^II^-Aβ_4−16_ shows a main irreversible peak at 1.04 V (vs. NHE) that is assigned to Cu^III^/Cu^II^ redox couple. An additional peak at lower redox potential (∼0.04 V vs. NHE) is assigned to the Cu^II^/Cu^I^ redox couple that can be mediated by reducing agent like ascorbate (Mital et al., 2015). Note that Aβ_4−42_ peptide has two copper binding sites, (1) N-terminal ATCUN site binds Cu^II^ strongly, suggesting redox silent site, and (2) bis-His motif (at 13^th^ and 14^th^ positions), binds Cu^II^ relatively low affinity, suggesting redox active site, that facilitates the reduction of Cu^II^ to Cu^I^. Upon addition of biological reductant i.e. ascorbate to Cu^II^Aβ_4-6_, Cu^II^ in bis-His is reduced to yield His-Cu^I^-His complex, while the remaining Cu^II^ in ATCUN site is unaffected (Figure 5) (Pushie et al., 2019). Nature designs such type of ATCUN site in Aβ_4−x_ peptide because it may trap Cu^II^ under oxidative stress conditions, but in excess, it crosses the ability to bind Cu^II^, and thereby developes Aβ toxicity. Therefore, designed ATCUN motifs could be used as Cu-chelators for treatment of AD (Folk and Franz, 2010; Robert et al., 2015). For instance, a designed ATCUN motif, such as DAHK (replica of HSA) has stronger binding affinity of ^c^K7.4 ∼ 10^14^ for Cu^II^ and shows redox silence against ascorbate (Gonzalez et al., 2018; Rozga et al., 2007). Upon chelation of Cu^II^ by DAHK from Aβ, the reduction of Cu^II^ is inhibited by ascorbate, and thereby retards ROS production.

In addition, the hydrophobic part of Aβ sequence, Aβ_16−20_ (KLVFF) has strong tendency for Aβ aggregation (Goyal et al., 2017). Therefore, Aβ_16−20_ represents a promising drug target for the development of the peptide based inhibitor. For instance, designed KLVFF peptide acts as an Aβ inhibitor and has good outcome (Goyal et al., 2017). Several experimental observations conclude that a hybrid complex, Cu-ATCUN-inhibitor strongly suppresses the aggregation of Aβ, as well as ROS formation compared to Cu-ATCUN or inhibitor alone (Zhang et al., 2016; Jensen et al., 2012). In this regard, ATCUN motif is conjugated with peptide inhibitor to generate a bifunctional peptide that has ability to disaggregate Aβ and also to chelate Cu^II^ ions. Faller et al. designed a bifunctional peptide by combination of DAHK with inhibitor (Aβ_12−20_ or Aβ_13−20_), that reduces the HO⋅ formation in cell culture (Jensen et al., 2012). Similar way, Yuan et al. designed a bifunctional peptide GGHRYYAAFFARR, which significantly suppressed the cytotoxicity of the Cu-Aβ complex (Zhang et al., 2016) (Figure 5). Based on this concept, Meng et al. have also reported, recently, a cocktail peptide, RTHLVFFARK, that retards the aggregation of Aβ, and thereby suppresses the cytotoxicity of Cu-Aβ_40_ in cell culture (Meng et al., 2018). These findings may guide in the development of peptide-based inhibitors for treatment of AD.

#### PET Imaging

**Figure 6:**
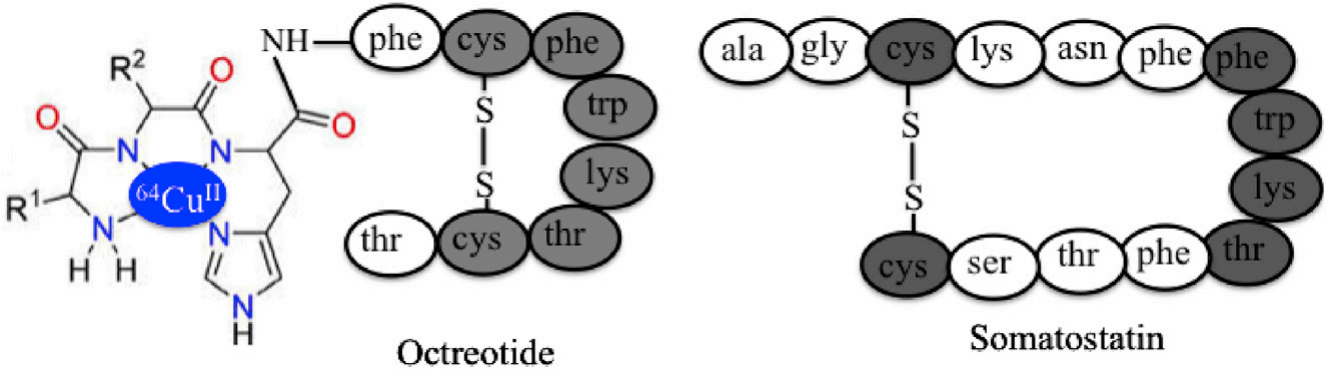
^64^Cu-ATCUN-Octreotide Model of Somatostatin Receptor (Targeting Tumor Cell) Providing PET Imaging R^1^ and R^2^; Y, V, N, T, G, and D amino acids.

The radioactive ^64^Cu^II^-ATCUN derivatives are thought to be a potential PET candidate for hypoxia (Wadas et al., 2010; Xie et al., 2016; Walke and Ruthstein, 2019). Hypoxia (or oxygen deficiency) is a hallmark of tumor-specific microenvironment (Brown and Wilson 2004). Therefore, PET-imaging of hypoxia is a very important technique to visualize the target molecule *in vivo*. For clinical diagnosis, the stability of metallo-peptide in blood plasma and transportation of metallo-peptide to the target site are key factors. Miyamoto et al., designed a series of ^64^Cu–ATCUN–octreotide derivatives (ATCUN = YYH, VVH, NNH, TTH, GGH, and DDH), where ATCUN motif is conjugated with tumor-targeting peptide, octreotide (Oct), and tested the stability of these derivatives in blood plasma for medical applications (Figure 6) (Miyamoto et al., 2016). Among them, ^64^Cu-YYH–Oct and ^64^Cu-VVH-Oct are highly stable in blood plasma (85.8% and 79.1%, respectively, after incubation of 2 hr). The octreotide, analog of somatostatin, has high affinity for somatostatin receptor, which is highly expressed in various tumor cells, mostly neuroendocrine tumor (Martino et al., 2010). Therefore, octreotide is used as a specific molecular target for imaging therapy (Sun and Coy, 2011). Among them, the ^64^Cu–YYH–Oct derivative shows the highest stability in blood plasma and reaches to target tumor cell in tumor-bearing mouse model (Miyamoto et al., 2016). Therefore, this cocktail complex, ^64^Cu-ATCUN-octreotide derivative is a potential candidate for PET-imaging. Therefore, more research is needed to design a more stable ^64^Cu-ATCUN derivative that will be exploited more in clinical practice for hypoxia imaging in future.

### Redox-Based Applications

Oxidation-reduction processes are considered as a heart of chemical and biological reactions. Nature uses proteins as scaffolds for building in metal cofactors, in order to control life at a minimal energetic cost. Chemists have learnt how to adopt naturally occurring scaffolds as catalysts and also explore for new performances. Beside the spectroscopic probe (above discussion), the designed metal-ATCUN derivatives, especially Cu-, Ni- and Co-ATCUN serve as a suitable catalytic metal-center for various catalytic activities, including DNA, RNA cleavage, protein inactivation (Harford and Sarkar, 1997; Mack and Dervan, 1992; Jin and Cowan, 2005, 2007; Agbale et al., 2016; Yu and Cowan, 2017a, 2017b; Gonzalez et al., 2018), small molecule activation (multi-electrons, multi-protons reactions) (Deng et al., 2018; Kandemir et al., 2016; Guo et al., 2018), as well as modification of biomolecules (protein-protein interaction, nitration of tyrosine) (Maiti et al., 2019; Horowitz et al., 2012; Brown et al., 1998). The catalytic metal center in M-ATCUN derivative works in two ways. One, the catalytic metal center generates ROS, which are mainly focused toward the cleavage or modification of biomolecules. Second, the same catalytic metal center activates the small molecules through the same redox cycle.

#### DNA Cleavage

Metallodrug is of significant interest in the development of nuclease (DNA and RNA cleavage) related human diseases at the genetic level, especially, cancer, human immunodeficiency virus (HIV), and hepatitis C virus (HCV) (Harford and Sarkar, 1997; Mack and Dervan, 1992; Jin and Cowan, 2005, 2007; Agbale et al., 2016; Yu and Cowan, 2017a, 2017b; Gonzalez et al., 2018; Mjos and Orvig, 2014). In 1983, Pauling et al. remarkably investigated (Kimoto et al., 1983) the antitumor activity of the Cu^II^-GGH (Cu-ATCUN) complex against Ehrlich ascites tumor cells. That investigation raised the interest in metal-ATCUN motifs toward DNA cleavage (Harford and Sarkar, 1997; Mack and Dervan, 1992; Jin and Cowan, 2005, 2007; Agbale et al., 2016; Yu and Cowan, 2017a, 2017b; Gonzalez et al., 2018; Cowan 2001; Detmer et al., 1996; Patwardhana and Cowan, 2001; Pamatong et al., 1996).

**Figure 7:**
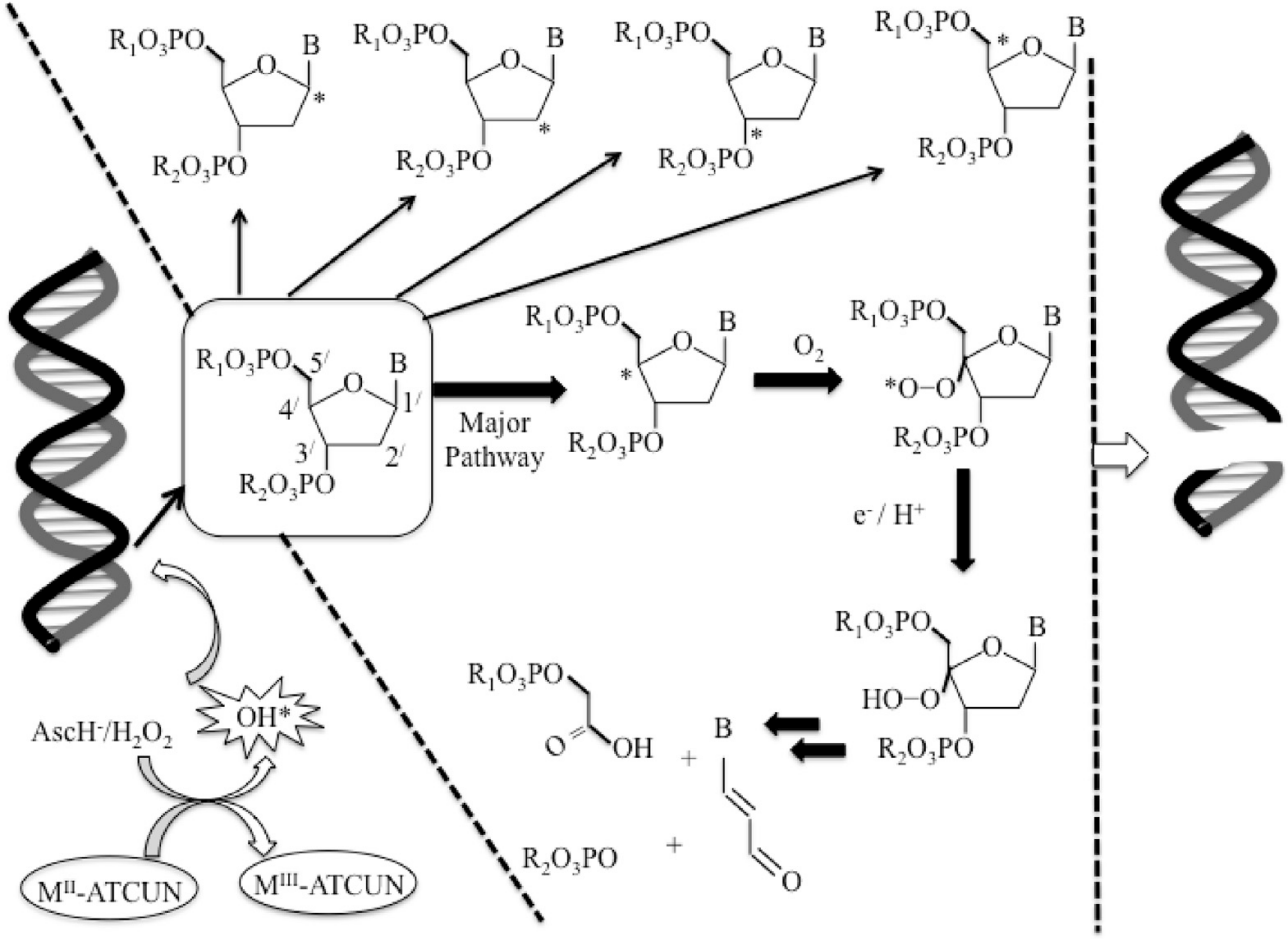
Schematic Representation of M-ATCUN Induces Oxidatively DNA Strand Scission by Sugar-Hydrogen Abstraction to Yield C_1_^/^-, or C_2_^/^-, or C_3_^/^-, or C_4_^/^-, or C_5_^/^-, Deoxyribosyl Radical. (modified from Pogozelski and Tullius, 1998). B = Nucleobase.

Generally, in presence of AscH^−^/H_2_O_2_, metal-ATCUN derivatives actively generate ROS such as hydroxyl radical (⋅OH) that facilitates DNA cleavage. Under physiological conditions, ⋅OH abstracts hydrogen atom from any carbon atoms (C_1_^/^, C_2_^/^, C_3_^/^, C_4_^/^, or C_5_^/^) of deoxyribose rings to initialize strand-breaks of DNA (Pogozelski and Tullius, 1998) leading to yield variety of cleavage products (Burrows and Muller, 1998) (Figure 7).

**Figure 8:**
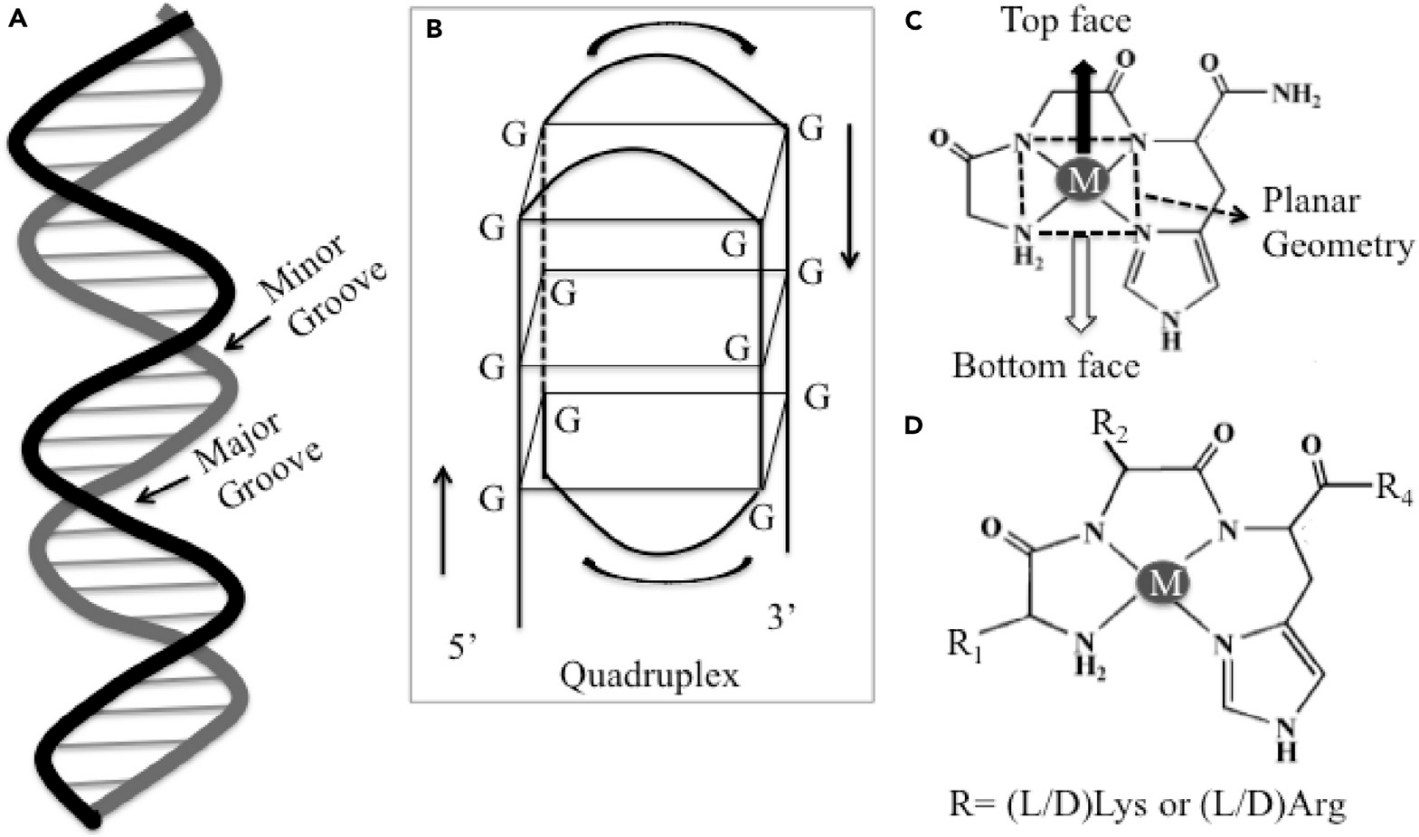
Selectivity and Efficacy of DNA Strand Scission Are Modulated by Proper Design of ATCUN Motif (A–D) (A) Two-dimentional representation of DNA showing targeting sites, minor and major groove, (B) quadruplex structure of telomeric DNA, (C) top and bottom faces of square planar geometry of M-ATCUN (M = Cu^II^, Ni^II^ and Co^II^), and (D) introduction of positive charge amino acid (positive charge and D/L) at first, second, and or fourth positions.

In practical applications, the selectivity and efficacy of DNA strand scission are modulated by proper design of ATCUN motif. The nuclease activity of M-ATCUN derivatives could be influenced by some key factors (Figure 8), including overall higher positive charge on M-ATCUN, inclusion of positively charged amino acids (such as Arg or Lys in ATCUN motif), planarity of catalytic site, stereochemical/geometrical orientation (D/L configuration), position and size of amino acid (Jiang et al., 2007). To date, the most common target sites of DNA are the minor and major groove and G-quadruplex (guanine-rich nucleic acid sequences of telomere DNA) (Figure 8) (Jiang et al., 2007).

To meet this DNA cleavage efficiency, varieties of M-ATCUN-derivatives have been developed. In this simple modular design of M-ATCUN, M^II^ ion is in a square planar configuration having two accessible binding faces that promote effective DNA cleavage (Mack and Dervan, 1990, 1992; Jin et al., 2007; Mack et al., 1988; Mahmoudi and Sarkar, 1999; Harford et al., 1996). Binding affinity of M-ATCUN toward DNA highly depends on side chain of amino acid at the periphery of ATCUN motif. When periphery functional groups of entirely designed M-ATCUN derivatives are the same molecular recognition of DNA (e.g. guanidinium, amine, and amide moieties), those M-ATCUN candidates selectively bind and effectively cleave the DNA strands (Harford and Sarkar, 1997; Mack and Dervan, 1992; Jin and Cowan, 2005, 2007; Agbale et al., 2016; Yu and Cowan, 2017a, 2017b; Gonzalez et al., 2018).

Historically, the Ni^II^-GGH derivative was first studied as a potential nuclease candidate (Mack and Dervan, 1992; Liang et al., 1998). Alternatively, nuclease activity was also being tested by Cu^II^-ATCUN derivatives. The intensive work was carried out on DNA cleavage by Cu/Ni-ATCUN derivatives. Long et al. have investigated extensively the nuclease activity by Ni-ATCUN derivatives. These derivatives preferential cleave the minor groove of DNA (at AT-rich regions) but catalytic efficacy depends on the stereochemistry, charge, and position of amino acids in ATCUN motif (Mack and Dervan, 1992; Liang et al., 1998). DNA cleavage chemistry by Cu/Ni-ATCUN derivatives has been also well documented by Cowan et al. (Jin and Cowan, 2005). For instance, the reactivity rate of Cu-KGHK derivative toward oxidative DNA cleavage is higher than the simple Cu-GGH derivative (Jin and Cowan, 2005). The other metal-derivatives, Ni^II^/Cu^II^-KKH and Ni^II^/Cu^II^-KGHK have been also studied where former M-derivative exhibits slightly higher cleavage rate than other (Jin and Cowan, 2005). This result indicates that the placement of Lys residue at first position in ATCUN has greater impact on cleavage efficiency than second position. It has been also accounted that the first position Lys (Arg analog) in KGH sequence is directed toward minor groove of DNA to generate iso-helical metallopeptide, which facilitates to yield stable DNA-ATCUN adduct, while the second position Lys is directed away from the minor groove of DNA. The slightly greater activity is simply accounted for by the strong electrostatic interaction between DNA (negative charge) and Lys or Arg (positive charge amino acid). In general, both Cu- and Ni-ATCUN derivatives show increase in DNA cleavage activity with increasing number of positive charge amino acid, such as Lys and Arg residues in ATUCN motif, that corroborate with redox potential (Jin and Cowan, 2005, 2007). Therefore, DNA cleavage activity does not depend only on positive charge of side chain amino acid residues but also important for proper orientation between Cu-ATCUN motif and DNA (Jin and Cowan, 2007).

**Figure 9:**
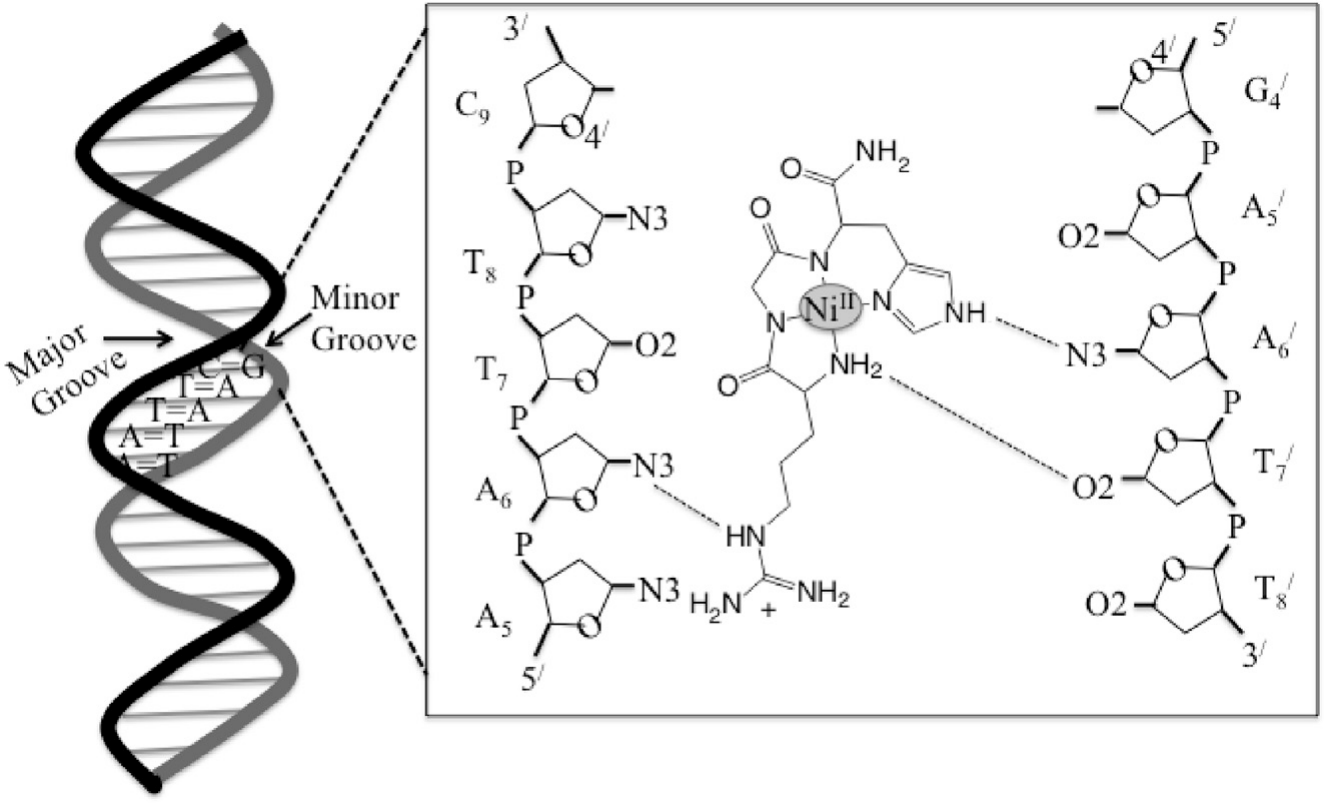
Two-Dimentional Representation of Interaction between Ni^II^-RGH and the Minor Groove of Model DNA, 5^/^-d(CGCG_4_A_5_ATTCGC_9_G)_2_ through H-Bonding (Dotted Lines) (Modified from Fang et al., 2004).

The DNA cleavage chemistry is not a random process where square planar geometry of M-ATCUN interacts at minor groove and abstracts mainly C_4_^/^-H from deoxy-ribose ring (Jin and Cowan, 2007; Pushie et al., 2019). The abstraction of C_4_^/^-H is facilitated by the formation of transient H bonds between square-planar “edges” of M-RGH (or Lys analog) derivative (H-bond donors, N-terminal N-H protons, imidazole pyrrole N-H, and Arg side-chain) and minor groove of AT-rich regions of DNA (H-bond acceptors; O2 of T and N3 of A) (Figure 9). The DNA cleavage is also highly influenced by the stereochemistry of amino acids (Fang et al., 2004, 2006; Nagane et al., 2001). Switching from L- to D-amino acids at positions 1 and 2 in ATCUN motif, the D-ATCUN derivative improves the nuclease activity but shows lesser selectivity. In comparison with L-ATCUN, the D-ATCUN derivative is sterically lesser hinder complex that allows M-ATCUN deeper insertion into minor groove of DNA, resulting increase the DNA cleavage efficiency (Fang et al., 2006).

Interestingly, Ni-ATCUN has ability to produce another reactive species, oxy-sulfur radical through the same redox cycling between Ni^III^ and Ni^II^ in presence of sulfite/oxygen, that also significantly damages the biomolecules including DNA, RNA, and proteins (Neta and Huie, 1985; Brandt and van Eldik, 1995). For instance, Ni-KGH shows the single- and double-stranded DNA damage in presence of Na_2_SO_3_ and O_2_ (Muller et al., 1997).

##### Cleavage of Telomeric DNA

**Figure 10:**
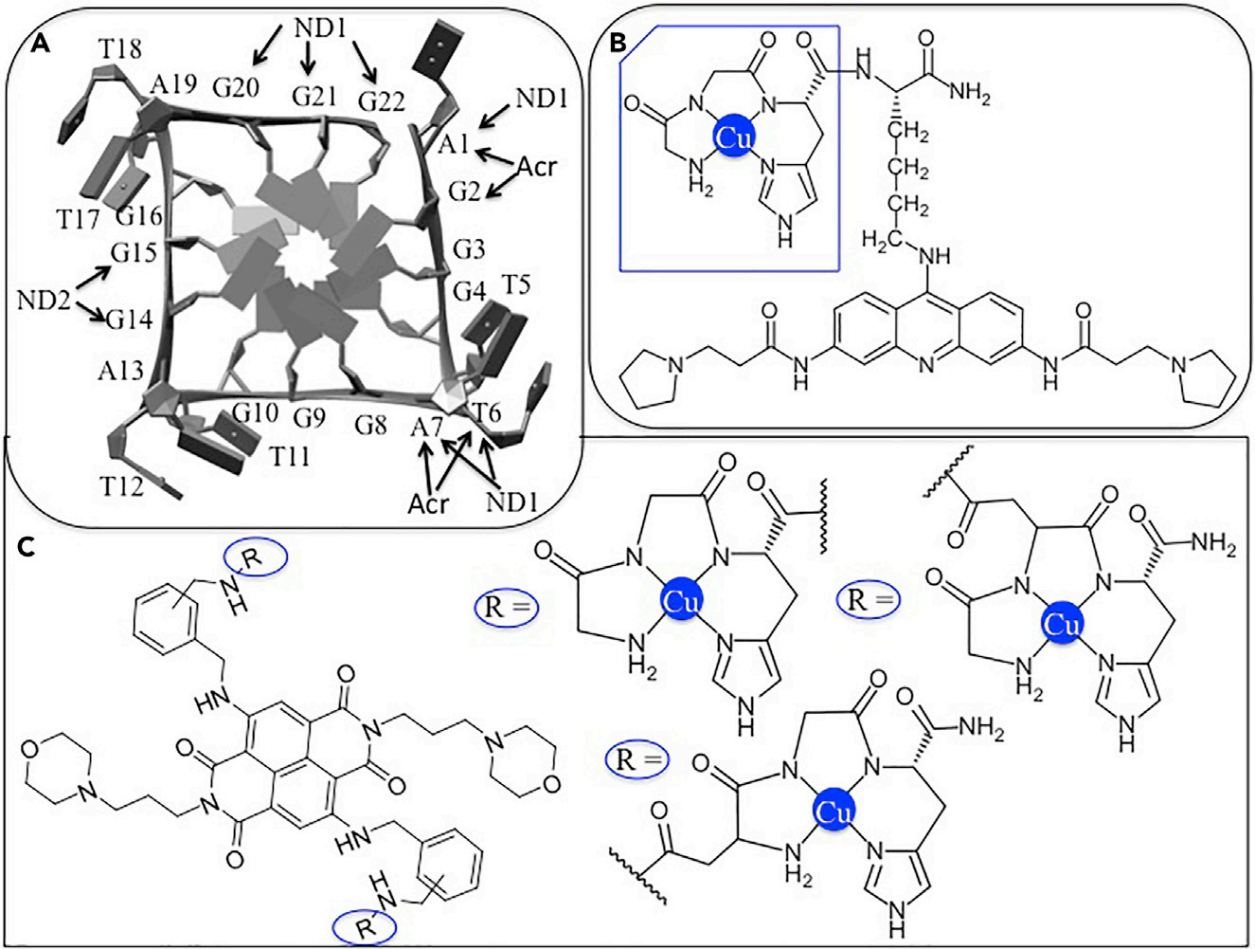
Cleavage of Telomeric DNA by designed Cu-ATCUNs (A) Crystal structure of G4 telomeric DNA (PDB: 1KF1 (Yu et al., 2015)) shows the selective cleavage sites by (Cu-ATCUN)_2_ND (black arrows with ND) and Cu-ATCUN-Acr (black arrows with Acr). ND; naphthalene diimide derivatives, ND1 and ND2 represents the two copper sites in (Cu-ATCUN)_2_ND, Acr; acridine. (B and C) (B) and (C) showing two-dimensional representation of Cu-GGH-Acr, and R_2_-ND derivatives (R = Cu-ATCUN = Cu-GGH, Cu-DGH and Cu-GDH) respectively (Modified from Yu et al., 2019).

DNA has another important therapeutic target site, G-quadruplex (G4) that is secondary structure of guanine (G)-rich sequence (TTAGGG) in telomeric DNA (Zahler et al., 1991). This G4-tetrad sequence is not read by the RNA template of telomerase complex (Xie et al., 2016), resulting downregulation of the telomerase activity in normal cells but up-regulation in tumor cells in human (>85%) (Kim et al., 1994; Sfeir et al., 2009). Therefore, G-quadruplex telomeric DNA plays an important role in cancer biology, suggesting as potential anticancer targets (Neidle 2017). To date, a large number of drugs have been published, but these are generally non-selective G4s cleavage drugs (Nadai et al., 2018). Alternative drugs are required for selective G4s cleavage. To achieve this goal, Cowan et al. (Yu et al., 2015) designed a Cu-GGHK-Acr derivative, where Acr (3,6,9-trisubstituted acridine) was a recognition domain of G-quadruplex (Figure 10B) that facilitated to bring the position of catalytic center, Cu-GGHK in close proximity to G4-tetrad through the π-π interaction between acridine ring and G4s surface (Yu et al., 2015; Campbell et al., 2008). So, this derivative selectivity cleaves the DNA sequence of G-quadruplex model (5^/^-FAM-dATT(TTAGGG)_3_) at A_1_-G_2_ and T_6_-A_7_ nucleotides sites, resulting inhibition of cancer cell division (Figure 10A) (Yu and Cowan, 2017a, 2017b; Yu et al., 2015). Cu-GGHK-Acr derivative has been also tested on cancer cell (MCF7 and HuH7) and shows significant anticancer activity against both cell lines.

It is expanded to design a series of G-quadruplex telomeric DNA cleavage models that show selective and rapid telomere reduction in cancer cell (Yu et al., 2019). In this assay, three different Cu-ATCUN derivatives (Cu-GGH, Cu-GDH, and Cu-DGH) are selected, and these are conjugated with naphthalene diimide (ND) derivative (as a G-quadruplex recognition moiety) through aspartate side chain (D) or C-terminal carboxylate of ATCUN motif (Figure 10C). Like acridine, ND also interacts with G-tetrad through π-π interaction that facilitates to bring the DNA cleavage moiety, Cu-ATCUN in close proximity to this G-tetrad. In addition, binding affinity of ND derivatives toward G-tetrad is higher (at least two-fold) than acridine isomer, resulting significant inhibition of cancer cell growth (Cuenca et al., 2008). Both Cu-GDH-(*p/m*)ND derivatives exhibit the highest level of telomere reduction in cancer cells. These (Cu-ATCUN)_2_-ND derivatives have two Cu-catalytic sites that have different binding patterns on G-tetrad. One Cu-center (ND1) is close to A_1_-A_3_, T_6_-A_7_, and G_20_-G_22_, whereas the second Cu-center (ND2) is close to G_14_, and G_15_ in G4-tetrad, resulting selective cleavage the nucleotide bases (Figure 10A). All (Cu-ATCUN)_2_-DN derivatives are also tested on human cancer cells (MCF7). Among them, (Cu-GDH)_2_-(*p*)DN significantly inhibits human cancer cell proliferation. Thus, the above observations emphasize to design efficient nuclease catalyst for the treatment of G4-related diseases.

#### RNA Cleavage

Like DNA, RNA is another important therapeutic target (Pearson and Prescott, 1997). RNA, a genetic component of retroviruses, is involved in a virus life cycle that causes viral infections, including HIV and HCV. The virus RNA is a complex structure possessing a set of sub-domains that are attractive for potential therapeutic targets. The cleavage of RNA in HIV or HCV by M-ATCUN has been studied through oxidative pathway like DNA (Joyner and Cowan, 2011; Bradford and Cowan, 2012; Jin and Cowan, 2006; Ross et al., 2015). RNA has some degree of resistance to oxidative cleavage with respect to DNA due to the presence of additional 2^/^-hydroxyl (2^/^-OH) in sugar-phosphate backbones (Thorp 2000). This 2^/^-OH attributes variety of secondary and tertiary structures, which originate a complicated reactivity pattern. However, the oxidative reactivity pattern of RNA has a firm relation with DNA, such as hydrogen abstraction. The oxidation of RNA is initiated by H-atom abstraction in presence of ROS, which is produced by catalytic metal center (Zhao et al., 2018; Crich and Mo, 1997). The selective RNA cleavage is also employed by an analogous strategy of DNA, where RNA cleavage domain (M-ATCUN) is coupled with RNA targeting domain.

##### HIV RNA Cleavage

**Figure 11:**
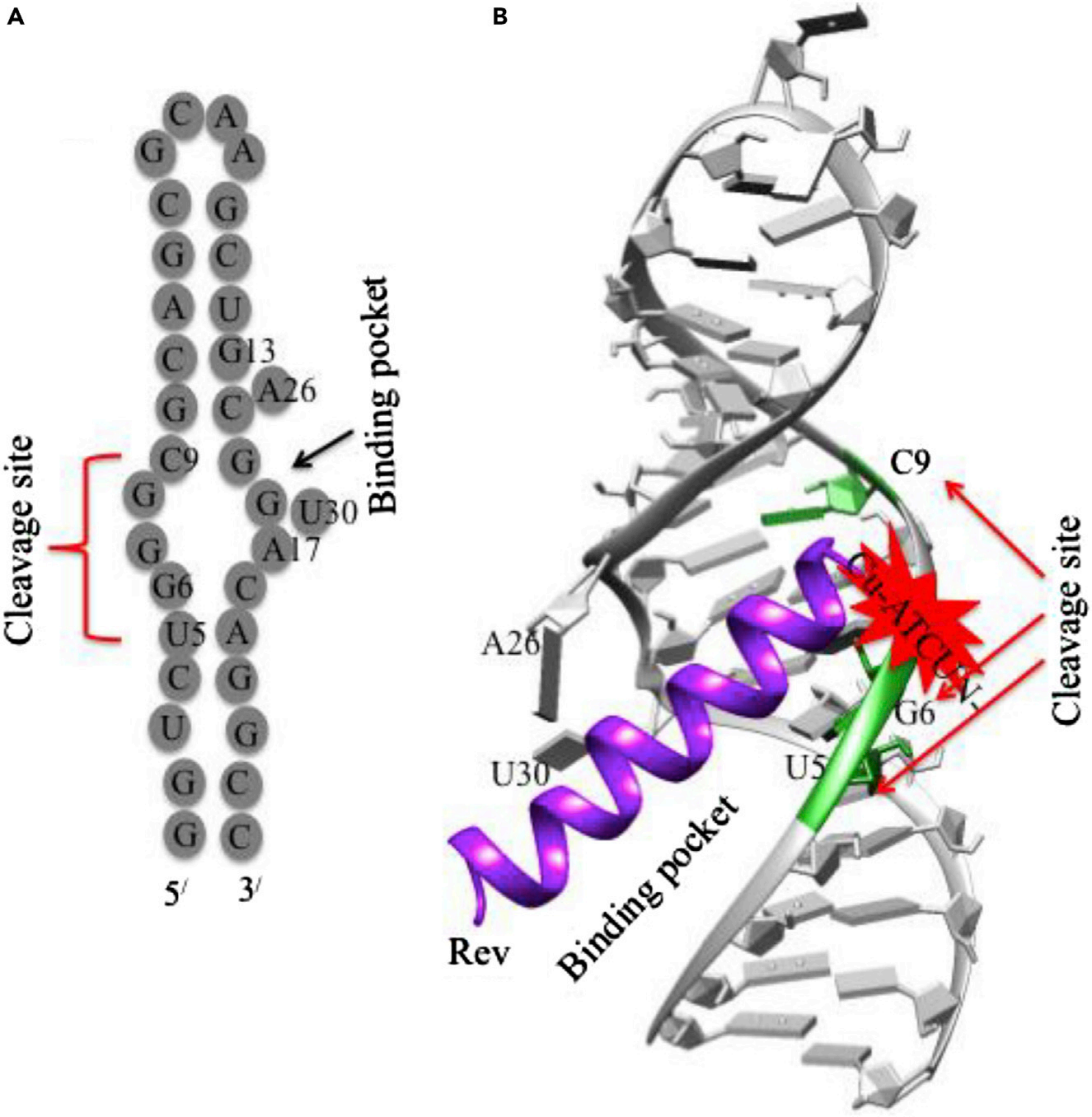
Selective Cleavage of HIV RNA by Cu-ATCUN-Rev (A) Schematic illustration of the stem loop IIb (SL-IIb) of RRE RNA of HIV showing the cleavage site, as well as binding pocket. (B) Crystal structure of RRE RNA SL-IIb complexes with an Rev peptide (purple) (PDB: 1ETF) showing the main cleavage sites which are shown by green highlighted as well as red arrows (U_5_, G_6_, and C_9_). The catalytic center, M-ATCUN (red star) may be conjugated with N-terminus of Rev. The structure is modified from Joyner et al. (2011).

HIV-1 gene expression is regulated by two virally encoded proteins, Rev and Tat. HIV-RNA binding protein (arginine rich peptide) is derived from the HIV-1 regulatory proteins, Rev that interacts with the Rev response element (RRE) of HIV-1 to generate the Rev-RRE complex (Battiste et al., 1996). The formation of this complex is an essential step in the viral replication cycle (Frankel and Young, 1998). To achieve this, Cowan et al. have designed antiviral metallodrugs, M-ATCUN-TRQARRNRRRRWRERQR (M = Cu/Ni/Co and ATCUN = GGH and KGHK) that selectively binds and oxidatively cleaves the RRE stem-loop of HIV RNA model (Joyner and Cowan, 2011; Jin and Cowan, 2006). The specific binding pocket (A_26_ and U_30_ nucleotides) and selective cleavage sites (C_9_, G_6_, and U_5_ nucleotides) in RNA are also shown in Figure 11. The adjacent G-G base pair in backbone of RRE RNA creates a specific binding pocket that allows the incoming Rev peptide for specific recognition (Battiste et al., 1996). The oxidative RRE RNA cleavage by Cu-GGH-Rev or Cu-KGHK-Rev derivative is similar to DNA cleavage where C4^/^-H abstraction is the major product (Joyner et al., 2013a, 2013b). Rationally designed Cu-ATCUN-Rev derivative is a promising candidate for selective cleavage of the RRE site of HIV to inhibit the virus replication cycle. The selective RNA cleavage by designed M–ATCUN derivatives has been well documented but at the same time, the practical requirements of cell delivery and activity *in vivo* of metallopeptide are also needed. A series of metallopeptides have been designed and evaluated the intracellular delivery and cleavage activity toward the target HIV-1 RRE RNA in both vitro and vivo (in *Escherichia coli* and mammalian cell) by cellular fluorescence assay.

**Figure 12:**
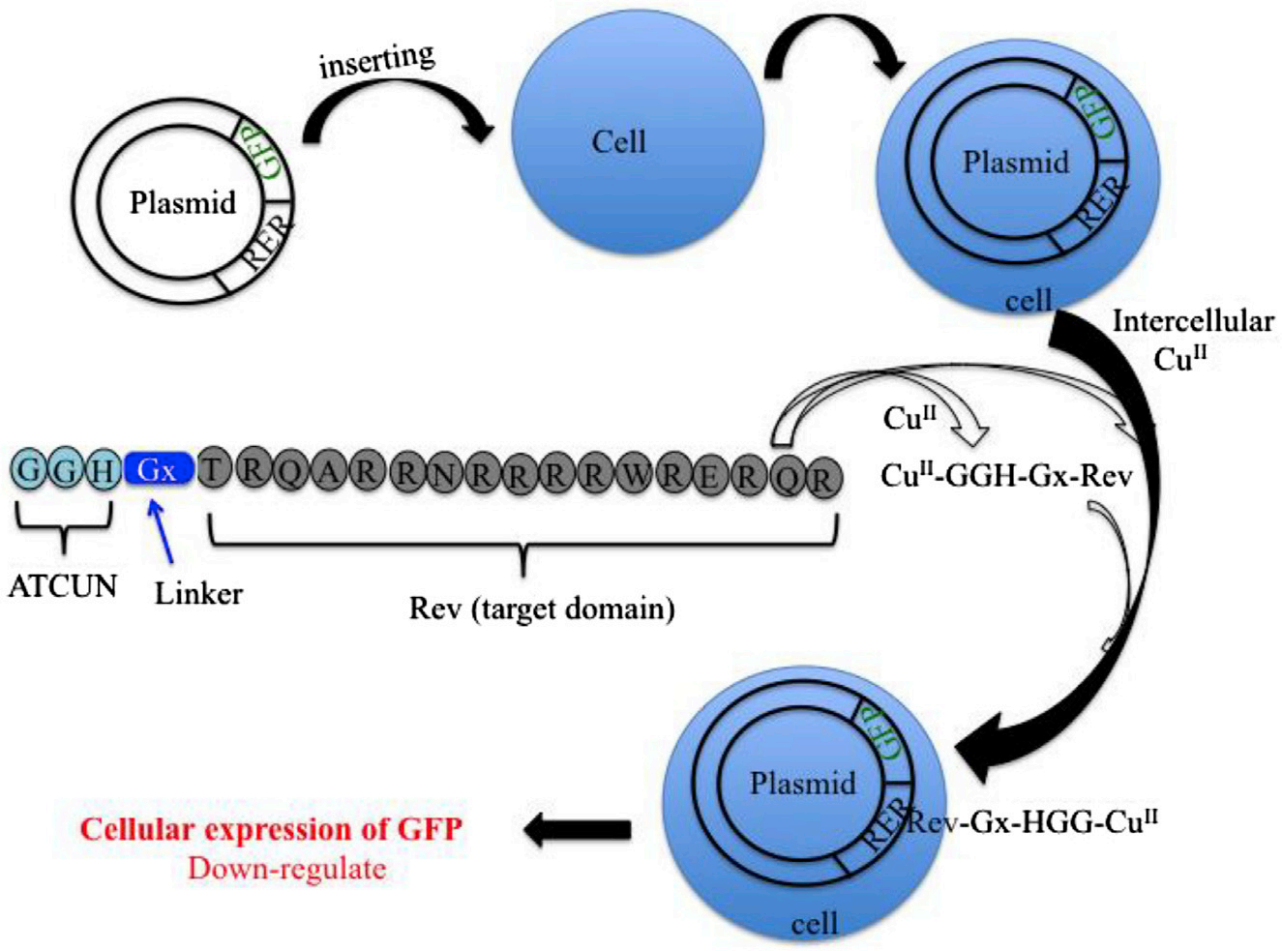
Schematic Representation of the Cellular Expression of GFP (Plasmid Encoded GFP-RRE) Is Induced by Cu^II^-ATCUN-Rev (Modified from Hocharoen and Cowan, 2009).

Figure 12 shows that the plasmid encoded RRE RNA sequence is fused to C-terminal of GFP. N-terminus of Rev peptide (TRQARRNRRRRWRERQR) is coupled with ATCUN (GGH) through a linker, Gx (glycine (G)x, x = 0, 1, 2, 4, 6 corresponding peptides are Rev1, Rev2, Rev3, Rev4, Rev5, and Rev6, respectively) (Jin and Cowan, 2007; Hocharoen and Cowan, 2009). Among them, Rev1, Rev2 peptides with shorter linker, and their copper derivatives significantly reduce the cellular expression of GFP (Plasmid encoded GFP-RRE), suggesting optimal RRE RNA cleavage activity. Interestingly, both metal bound or metal-free state of Rev1 and Rev2 derivatives show similar cellular activity, suggesting both metal free derivatives are able to recruit the metal ions from a cell by ATCUN motif.

Another important site-specific RNA cleavage target is the hairpin-loop (stem loop), trans-activation-responsive region (TAR) RNA from HIV-1 that serves as specific binding site for arginine-rich tat protein (RKKRRQRRRPPQ) or Tat derived peptide (Aboul-ela and Varani, 1998). Long et al. reported that all Ni^II^-Xaa-Gly-His (X_aa_ = Gly, Lys, or Arg) derivatives selectively cleaved the stem loop of TAR RNA of HIV-1 (Brittain et al., 1998).

##### HCV RNA Cleavage

**Figure 13:**
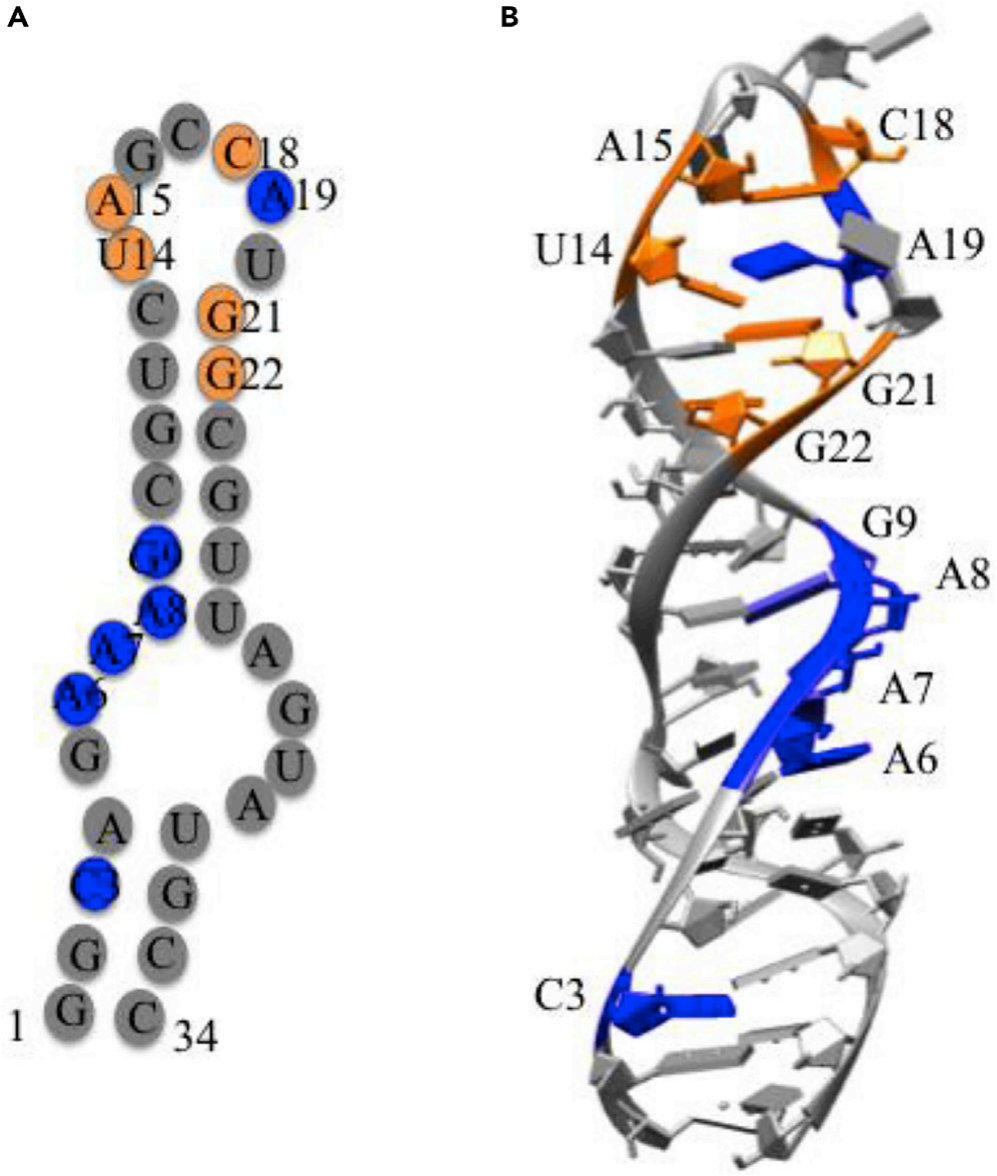
Selective Cleavage of HCV RNA by M-ATCUN Derivatives (A and B) (A) Two-dimensional representation of subdomain SL-IIb RNA of HCV, and (B) crystal structure of HCV SL-IIb RNA (PDB: 1P5N) showing the main cleavage sites (highlighted blue and orange color) by CuGGHYrFK or CuGGhyrfk respectively. Modified from Ross et al. (2017).

The designed catalytic metallodrugs, M-ATCUN derivatives also cleave the RNA of HCV selectively. The internal ribosomal entry site (IRES) of HCV RNA is an important component for the life cycle of the HCV. Therefore, HCV IRES RNA containing stem loop IIb and IV (SLIIb and SLIV) are promising therapeutic targets (Figure 13) (Kieft et al., 2001; Lukavsky et al., 2003). Based on previous approach, M-GGH-YrFK derivative (Lower case ‘r’ represents D-arginine) is designed, which selectively binds and cleaves the stem loop-IIb (SLIIb) of the HCV IRES (Bradford and Cowan, 2012). The tera-peptide, YrFK, a dermorphin analog known as DALDA, highly specific μ-opioid receptor agonist (Samii et al., 1994), binds to targeting domain, SL-IIb of HCV IRES (Bradford and Cowan, 2012). The designed metallodrug, Cu-GGH-YrFK-amide derivative is examined on model oligonucleotide (5′-fluorescein-GGCAGAAAGCGU- CUAGCCAUGGCGUUAGUA UGCC-3′) of SL-IIb *in vitro* and in HCV cell. The cellular replicon assay of Cu-GGH-YrFK-amide derivative shows enzyme-like turnover (K_M_ of 0.85 μM and k_cat_ of 0.53 min^−1^). The incorporation of D-Arg (r) into YrFK sequence enhances the binding affinity and reactivity of Cu-GGHYrFK toward SLIIb of IRES RNA. In addition, a designed Cu-GGh-yrfk derivative (lower case indicates the D-form of amino acid) is also evaluated for binding and reactivity patterns against RNA of HCV. Both compounds show the similar HCV cellular replicon assay and high stability *in vivo* that meet to the US-FDA approved HCV replicon assay (Bradford et al., 2014). Interestingly, Cu-GGh-yrfk, exhibits major cleavage sites, U_14_ and A_15_, whereas Cu-GGH-YrFK exhibits a wider range of cleavage sites A_6_-G_9_ of HCV SLIIb RNA (Figure 13). This concept is expanded to design a series of L-analog of Cu-GGH-YrFK derivative where recognition domain, YrFK is shuffled with existing amino acid or substituted other amino acids like A, D, N, and K (YRFK, ARFK, YAFK, YRAK, YRFA, KFRY, DRFK, NRFK, KRFK, and FRFK) in order to understand the impact of each amino acid residue toward SLIIb RNA binding and cleavage efficiency (Ross et al., 2017). Binding affinity and reactivity patterns of each metallo-peptide are modulated by stereochemistry, charge, size and position of amino acids in the targeting domain. For instance, Cu-GGh-YRFK and Cu-GGH-FRFK show the low catalytic efficiency compared to Cu-GGH-YrFK and Cu-GGh-yrfk (Ross et al., 2017).

**Figure 14:**
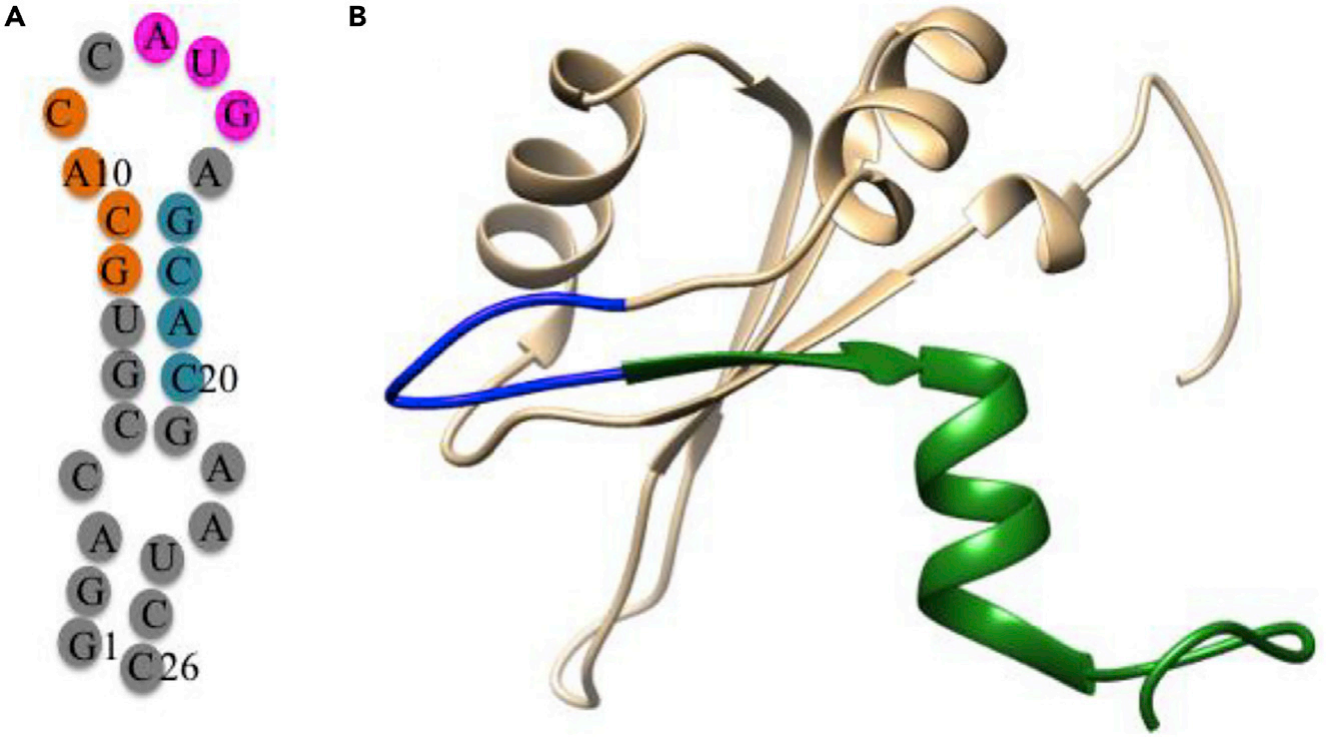
Inhibition of HCV Replication by La Protein (A) Two-dimensional representations of SL-IV RNA showing AUG start codon (pink circle) and 5′-GCAC-3′ sequences as a potential recognition site (orange circle). (B) NMR solution structure of the human La protein is derived from PDB 1S79. LaR2C peptide, KYKETDLLILFKDDYFAKKNEERK is highlighted as green and blue color. The truncated 7-mer, KYKETDL is highlighted as blue color. Modified from Ross et al. (2015).

Another promising therapeutic intervention of metallopeptide is stem loop IV (SL-IV) of HCV IRES RNA. SL-IV possesses ribosome assembly GCAC domain and AUG start codon (Mondal et al., 2008) (Figure 14). The LaR2C peptide, analog of human La protein, binds with SL-IV HCV IRES resulting inhibition of HCV replication (Pudi et al., 2003). The HCV replicon assay of designed metallodrugs, Cu-GGH-KYKETDLLILFKDDYFAKKNEERK and Cu-GGH-KYKETDL containing LaR2C peptide (KYKETDLLILFKDDYFAKKNEERK and truncated sequence, KYKETDL of La protein are shown in Figure 14), are examined on model oligonucleotide (5^/^-fluorescein-GGACCGU**GCAC**C**AUG**AGCACGAAUCC-3^/^) of HCV IRES SLIV RNA *in vitro* (Ross et al., 2015). In this replicon assay, both derivatives show significant efficacy. Cu-GGH-KYKETDLLILFKDDYFAKKNEERK exhibits most significant cleavage sites, G_15_ and A_16_, whereas Cu-GGH-KYKETDL exhibits the main cleavage site, G_8_, representing a part of GCAC domain. Still need to develop these drugs for the treatment of human HCV infection.

#### Inhibition of Protein Activity

Inhibition of enzyme activity by drugs is another novel approach to cure the human diseases. Basically, drug blocks the active site of enzyme to inhibit the enzyme activity, and thereby arrests the enzyme related diseases. The diseases associated proteins such as human angiotensin converting enzyme (ACE) (Gokhale and Cowan, 2005; Joyner et al., 2012; Hocharoen et al., 2013), carbonic anhydrase-I (Gokhale et al., 2008), and sortase-A (Fidai et al., 2014) are potential therapeutic targets. Herein, M-ATCUN is conjugated with substrate inhibitor to generate a cocktail complex that basically plays the dual role: (a) selective binding and (b) oxidative modification of amino acid residues in proteins/enzymes.

##### Human ACE

**Figure 15:**
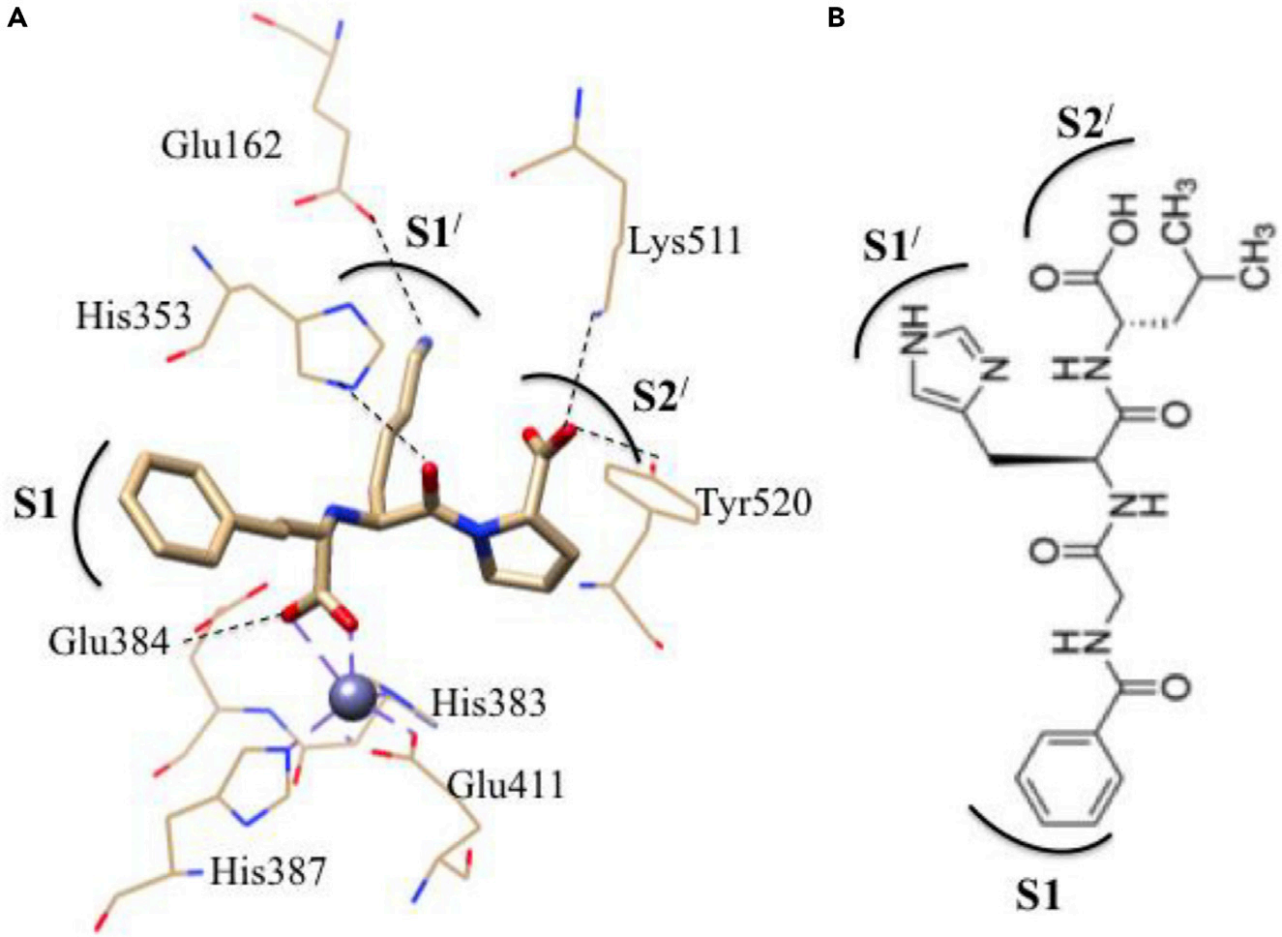
Interaction between Zn-ACE and Inhibitor (A and B) (A) Partial crystal structure of lisinopril (inhibitor) adduct Zn-active site of ACE (PDB code: 1O86 (Natesh et al., 2003)), and (B) two-dimentional representation of natural occurring substrate, Hip-His-Leu are showing the binding sites. S1; benzene group, S1^/^; His group and S2^/^; dimethyl group of natural substrate sites corresponding to lisinopril.

The ACE, a Zn-containing metalloenzyme, converts the natural deca-peptide (DRVYIHPFHL) angiotensin I to angiotensin II as a potent vasoconstricting octapeptide (DRVYIHPF) and degradation of vasodilator nanopeptide bradykinin (RPPGFSPFR), leading to increase in blood pressure (Crackower et al., 2002). ACE inhibitor such as lisinopril is a potential drug candidate for the treatment of hypertension and heart failure. The binding mode of lisinopril (N2-[(S)-1-carboxy-3-phenylpropyl]-L-lysyl-L-proline) with Zn-site in ACE is shown in Figure 15A (Natesh et al., 2003). Interestingly, the backbone of lisinopril is very close to ACE substrate, Hip-His-Leu that contains benzene group at S1 site, His group at S1^/^site, and dimethyl group at S2^/^site of lisinopril (Figure 15B).

**Figure 16:**
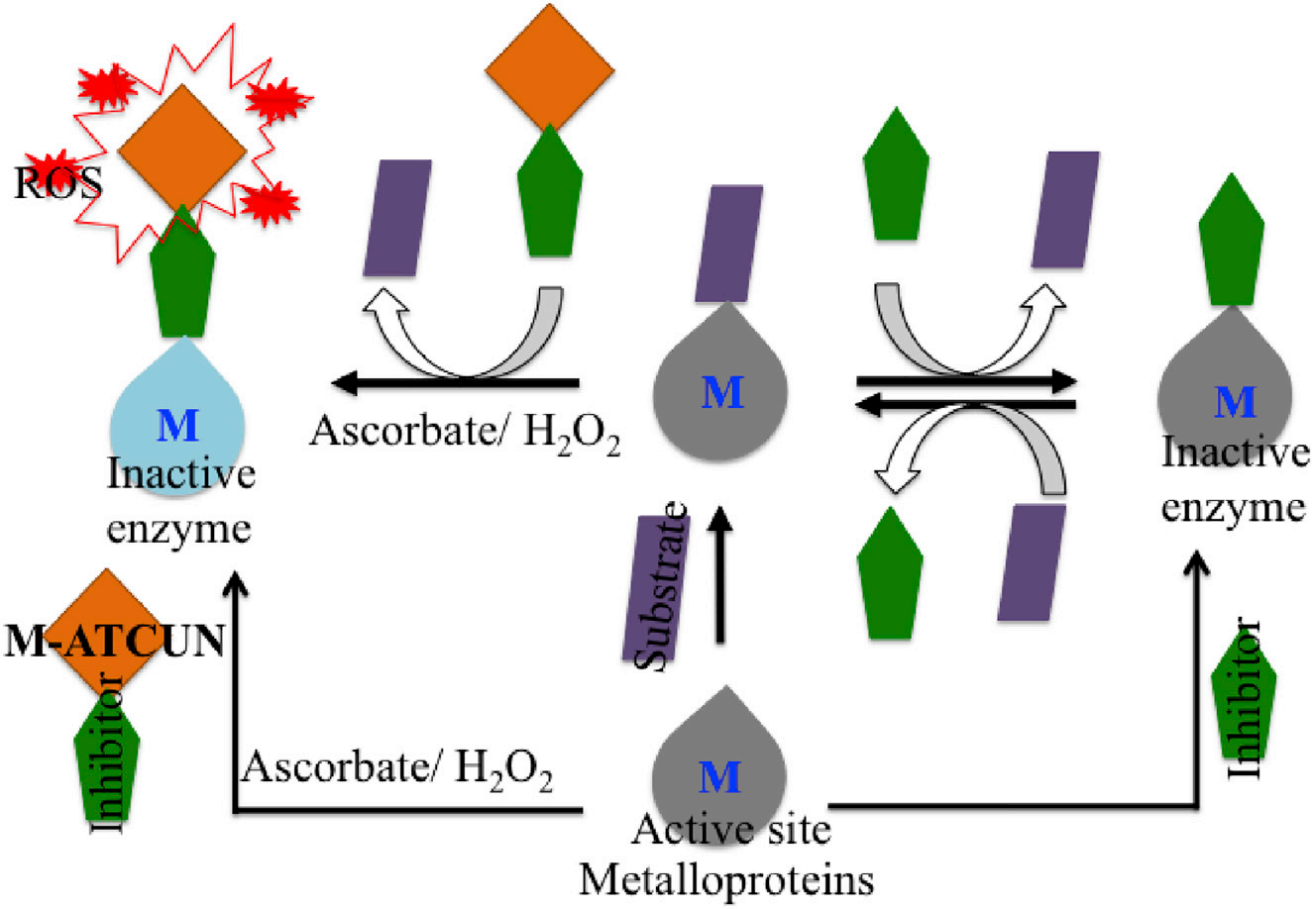
Possible Reversible (Simple Inhibitor) vs. Irreversible (M-ATCUN-Inhibitor) Inactivation Mechanism of Metalloproteins-Substrate with Inhibitor

In general, lisinopril (inhibitor) makes inactive the somatic-ACE-1 (isoform of ACE family) activity by replacing the substrate from metalloenzyme-substrate complex to yield metalloenzyme-inhibitor complex, but it is a reversible process. The alternative of reversible inhibitor (lisinopril) is an M-ATCUN-inhibitor derivative that plays for irreversible inactivation of sACE-1 enzyme by modification of biomolecules through oxidative pathway (Figure 16).

Cowan et al. demonstrated the catalytic inactivation of ACE enzyme by using a designed Cu-KGHK derivative. The attachment of Lys in KGHK is mimicked to the lysine side chain of lisinopril. The inactivation assay of human somatic ACE-1 (Soubrier et al., 1998) is monitored by the cleavage of the fluorogenic peptide, Mca-RPPGFSAFK(Dnp)-OH (Mca; methyl coumarin, Dnp; dinitro-phenyl) (Johnson and Ahn, 2000) as a model of bradykinin. This assay shows the catalytic inactivation (rate constant, k ∼ 2.9 ± 0.5 X 10^−2^ min^−1^) of ACE enzyme with pre-incubation of Cu-KGHK derivative under oxidative conditions (Gokhale and Cowan, 2005). Based on this result, a new metallodrug, Cu-GGH-lisinopril is developed that selectively binds somatic ACE-1 with high affinity (Joyner et al., 2012).

The somatic-ACE harbors two homologous N- and C-domains (Hocharoen et al., 2013). The interest is to develop metallo-peptide that selectively binds the particular domain in somatic-ACE. Molecular modeling studies on Cu-GGH-linsinopril within sACE-1 reveals that the Cu in Cu-GGH-lisinopril coordinates nearby D140 in N-terminal domain, whereas E162 or D377 in the C- terminal domain of sACE-1 (Joyner et al., 2012). The catalytic inactivation of each N/C-domain of somatic ACE-1 is examined by designed M-ATCUN-lisinopril derivative (Hocharoen et al., 2013). The M-ATCUN-lisinopril complex shows significant higher catalytic inactivation rate in N-domain over C-domain of somatic ACE, suggesting optimal orientation of M-chelate-lisinopril complex within N-domain compared to C-domain (Hocharoen et al., 2013). These results provide a valuable drug development for other therapeutic targets.

##### Carbonic Anhydrase

**Figure 17:**
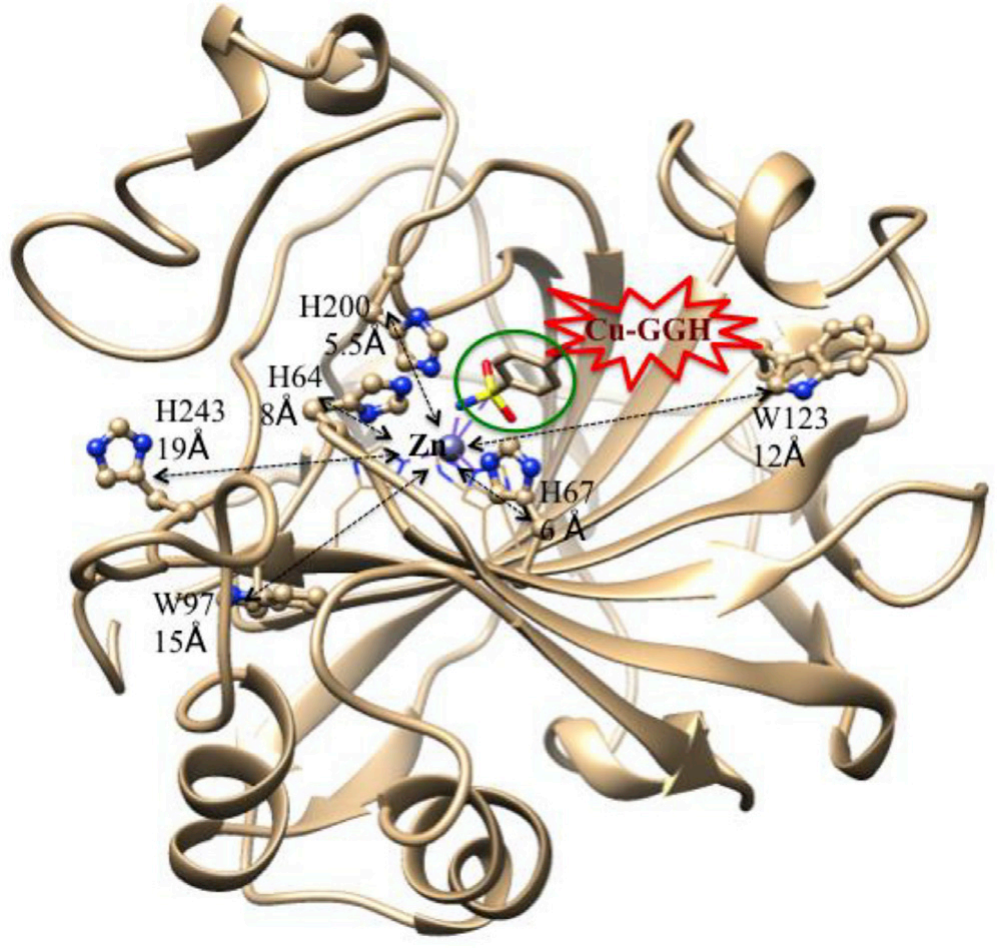
Crystal Structure of Human CA-I (PDB: 1CZM (Chakravarty and Kannan, 1994)) with Bound Sulfonamide Inhibitor (Stick Model and also Highlighted by Green Circle) Highlighted the possible oxidatively modified amino acids, H_64_, H_67_, H_200_, H_243_, W_97_, W_123_ (stick ball model) by Cu-GGH. Distances of these residues from Zn-center are shown by dotted double headed arrows. The Cu-GGH (red star) is assumed to coordinate to the NH_2_-tail of sulfonamide inhibitor (green circle). Modified from Gokhale et al. (2008).

Another Zn-containing metalloenzyme is carbonic anhydrase (CA), which is responsible for the rapid conversion of CO_2_ to HCO_3_^-^ and protons, involving in various physiological and pathological processes (Supuran et al., 2003). Overexpression or elevated CA is related to various diseases, which include human diabetes, heart failure, cancer, and glaucoma (Supuran et al., 2003; Supuran 2008). So, CA is an important therapeutic target. The activity of CA is suppressed by a substrate inhibitor, sulfonamide derivative through CA(Zn)-sulfonamide complex formation (Figure 17) (Khadikar et al., 2005). This advantage of sulfonamide derivative, *p*-aminobenzene-sulfonamide (SLN) is utilized as a recognition domain and it is conjugated with a catalytic domain, Cu-ATCUN (Cu-GGH) to yield a metallo-inhibitor, Cu-ATCUN-SLN. This derivative selectively binds at the active site of human CA-1 and oxidatively modifies the possible close proximity (5–20 Å) amino acid residues, including H_64_, H_67_, H_200_, H_243_, W_97_, W_123_. Interestingly, no modification of Zn coordinated His residues (H_94_, H_96_ and H_119_), as well as no cleavage of protein are observed (Gokhale et al., 2008).

##### Sortase A

**Figure 18:**
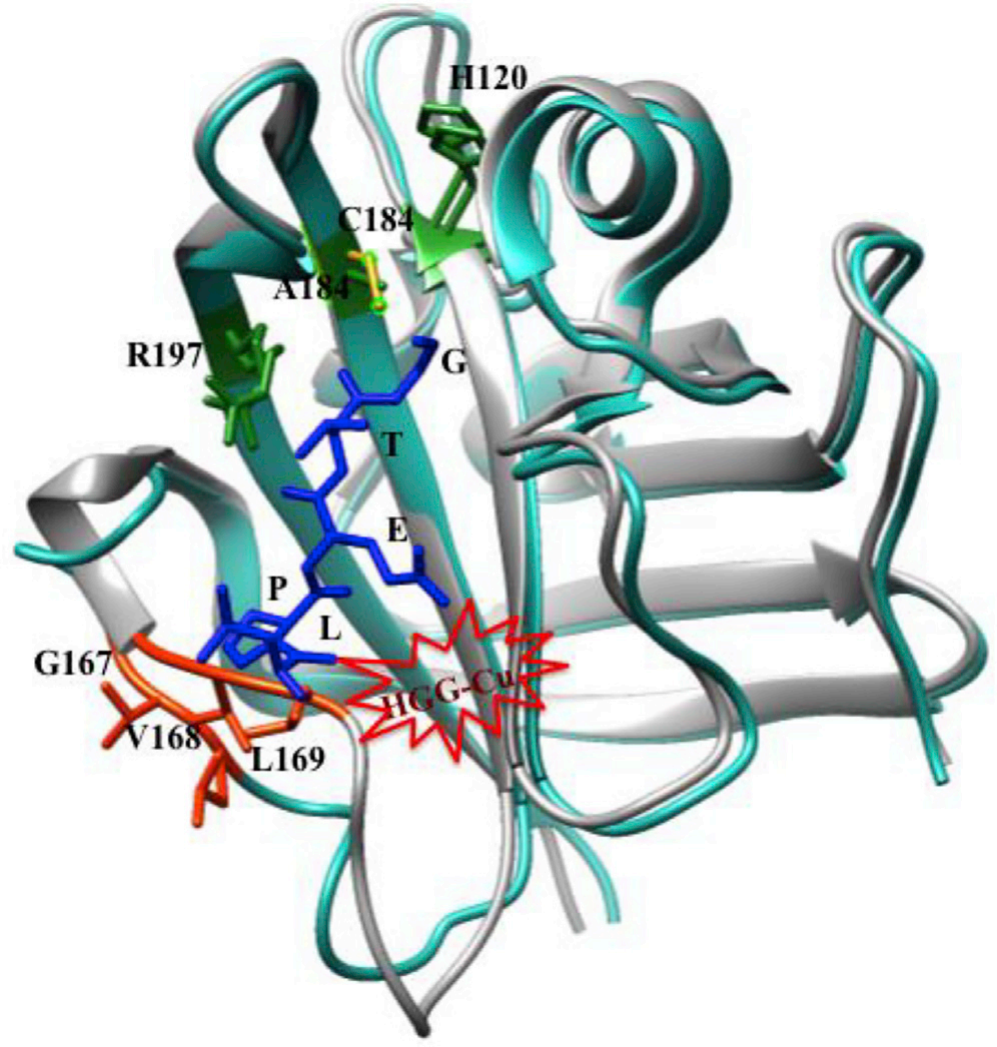
Inactivation of SrtA_ΔN59_ Activity by Cu-ATCUNs Superimpose crystal structures of SrtA_ΔN59_ (Wild (cyano) (PDB ID: 1T2O (Suree et al., 2009)) and mutant Cys184Ala (gray) (PDB ID: 1T2W (Suree et al., 2009)) with substrate, LPETG (blue)). Cu-GGH (red star) is assumed to bind to the L residue of substrate where close proximity amino acid residues G_167_, V_168_, and L_169_, and G residue of substrate points toward the active-site amino acid R_197_-C_184_-H_120_. Modified from Fidai et al. (2014).

Sortase A (SrtA), a membrane-bound bacterial surface adherence protein found in gram positive bacteria (staphylococcus aureus), is a common cause of many serious hospital- and community-acquired infections and cleaves the amide backbone in-between Thr (T) and Gly (G) residues in LPXTG motif of the C-terminal cell-wall sorting signal (Cossart and Jonquires, 2000). The bacterial infection can be halted by inactivation of SrtA protein (Aboul-ela and Varani, 1998). The designed metallopeptides, Cu-GGHLPETG, Cu-GGHLPET, Cu-GGHGLPETG, and Cu-GGHGLPET containing SrtA-targeting motif, LPET or LPETG (Fidai et al., 2014; Zong et al., 2004), selectively bind and suppress the activity markedly, resulting inactivation of SrtA protein. The mechanism of enzyme inactivation describes the oxidative modification of the enzyme active site by Cu-GGH catalytic domain. The head (G) of SrtA substrate, LPETG, is directed toward the active site containing conserved amino acid sequence, R_197_-C_184_-H_120_. The tail (L) of LPETG is coordinated with Cu-ATCUN catalytic domain that can modify the close proximity amino acid residues, R_197_-C_184_-H_120_, but there is no evidence for modification of Gly_167_, Val_168_, and Leu_169_ residues (Figure 18) (Fidai et al., 2014). Interestingly, shortest (GGHLPET) and longest (GGHGLPETG) sequences of metallopeptide show a significant higher rate of enzyme inactivation than the moderate-size (GGHGLPET) of metallopeptide. The shortest peptide provides the better alignment of the catalytic domain, Cu-ATCUN toward the enzyme active site (R_197_-C_184_-H_120_), resulting higher rate of enzyme inactivation, whereas longest peptide provides the catalytic center far away from the enzyme active site, but it gives more solvent exposed catalytic center that produces more ROS. Overall, the shortest metallopeptide shows higher rate of Cys oxidation in active site of enzyme (Bradford et al., 2014; Fidai et al., 2014).

##### Peptide-Based Inhibitor of Viral Protease

**Figure 19:**
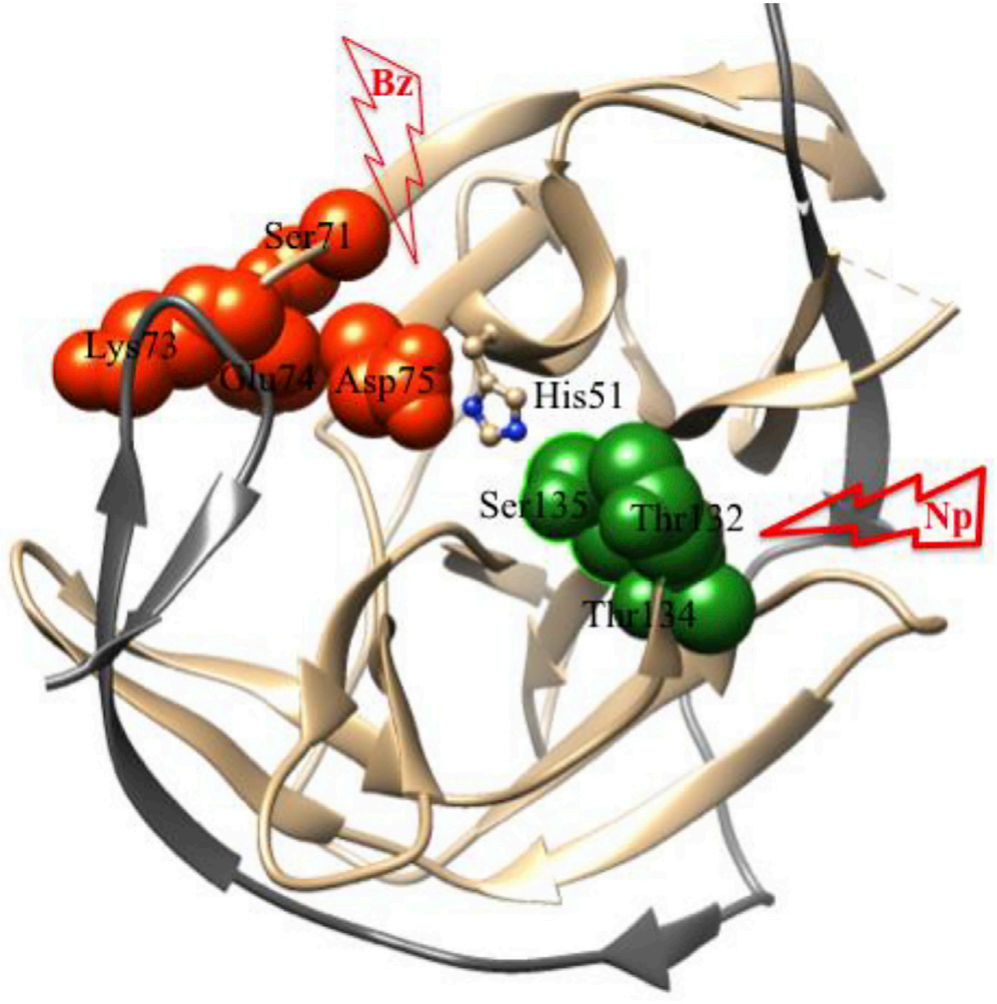
Peptide-Based Inhibitor of Viral Protease Crystal structure showing oxidative damage to West Nile Virus NS2B/NS3 (PDB: 2FP7). Amino acid residues, green set (Ser135, Thr_132_ and Thr_134_) and orange set (Asp_75_, Ser_71_, Lys_73_ and Glu_74_) are modified by naphthoylated (Np) and benzoylated metallo-peptide (Bz) respectively. His_51_-Asp_75_-Ser_135_ triad is active site. Modified from Pinkham et al. (2018a).

The viral protease, NS2B/NS3 (non-structural protein) is an essential for viral replication and maturation, which is a promising pharmaceutical target for flavivirus infections, including west Nile (WNV), japanese encephalitis, yellow fever (YFV), zika (ZKV), and dengue (DENV) viruses (Chappell et al., 2008). NS2B-NS3 protease belongs to serine protease, trypsin-like, with a classic catalytic triad, His-Asp-Ser (Kang et al., 2017; Bazan and Fletterick, 1989). The peptide-based inhibitor therapy is one of the most potential therapeutic strategies for treatment of various viral infections but currently no drugs are available in market. To achieve this, the peptide-based inhibitor is designed by introduction of ATCUN (GGH) motif into the N- or C-terminus of WNVP targeting peptides such as naphthoylated, benzoylated, or acetylated (Pinkham et al., 2018a, 2018b). All metallo-peptide derivatives show noticeable decrease in WNVP activity by oxidative modification of amino acids. Particularly, His_51_-Asp_75_-Ser_135_ triad in WNVP is essential for substrate binding and enzyme activity (Figure 19) (Pinkham et al., 2018a, 2018b). Overall, the naphthoylated metallo-peptide modifies the active site amino acid residue, Ser_135_ and additionally Thr_132_ and Thr_134_ residues whereas benzoylated metallopeptide modifies the active site amino acid residue, Asp_75_ and additionally Ser_71_, Lys_73_ and Glu_74_ residues, which ultimately lead to the enzyme inactivation.

Overall, designed metallo-peptide inhibitors (Cu-ATCUN-substrate) give deeper insights on catalytically inactivation of enzymes activity through protein-substrate interactions, as well as oxidative damage the amino acid residues, but it remains in cellular replicon study yet. These results help to develop the drugs in future for antiviral activity in clinical treatments.

#### Antimicrobial Activity

**Figure 20:**
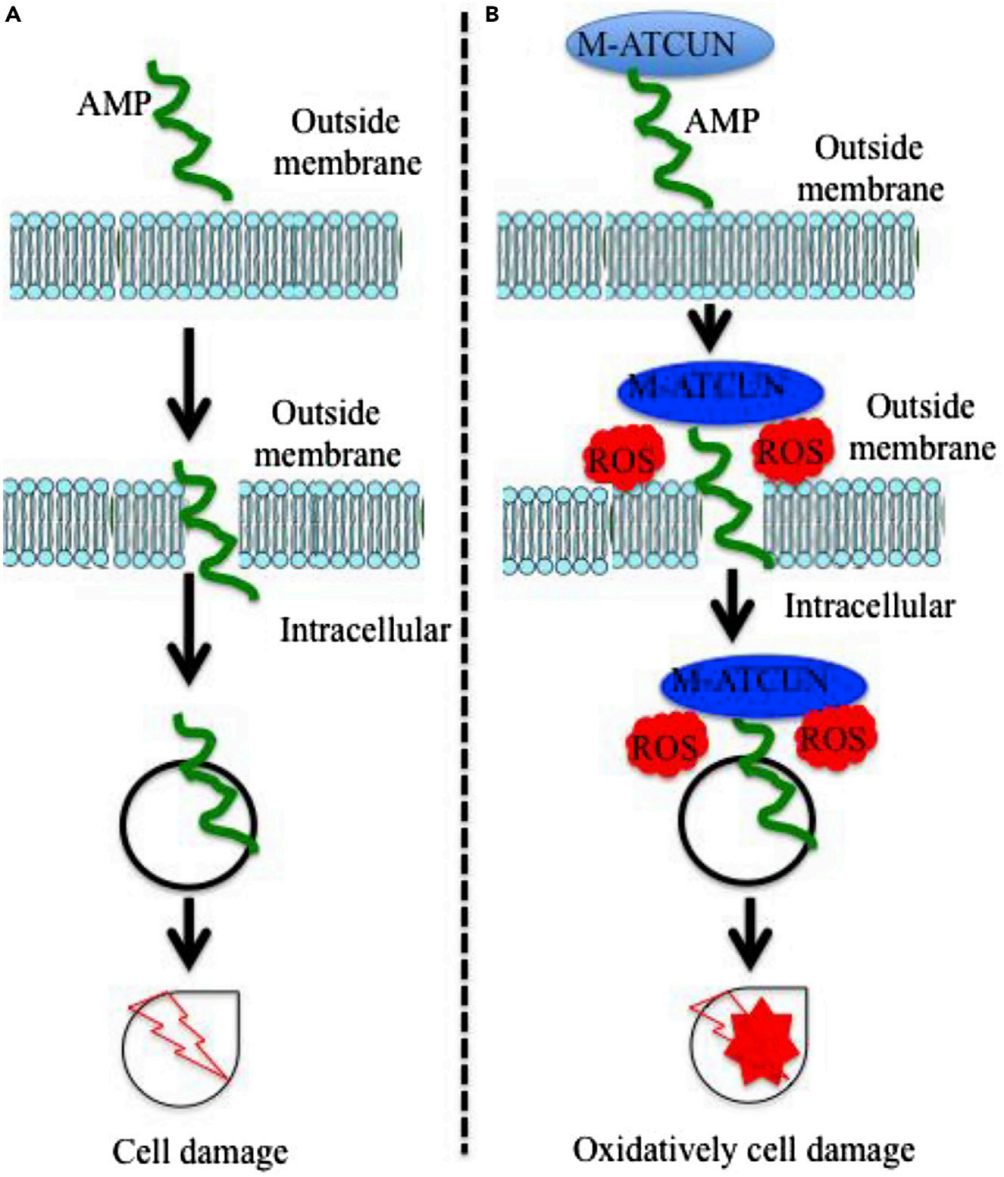
Possible Mechanisms of Bacteria Cell Death by M-ATCUN-AMPs vs. AMPs

Worldwide human health is threatened by multi-drug resistant pathogens. Currently, naturally occurring AMPs have great potential in drug development due to their wide range of activity toward multi-domain pathogens. Interestingly, the ATCUN motif is also found in naturally occurring AMPs, including histatin, hepcidin, and piscidin, which serve as natural antibiotics (Alexander et al., 2018; Hancock and Sahl, 2006; Zasloff 2002). M-ATCUN derivatives are to be a novel therapeutics agent against multi-domain pathogens. These peptides attack the cell membrane of many pathogens (Gram-negative and Gram-positive bacteria and fungi) through a common mode of action, such as cell membrane disruption (Brogden 2005; Dawson and Liu, 2006). Human saliva protein, histatin is one of the best natural examples of AMP that kills many pathogens, suggesting a promising candidate for drug development (McCaslin et al., 2019; Melino et al., 2006). Melino et al. designed a model of histatin-5 (DSHAKRHHGYKRKFHEHHSHRGY), known as ATCUN-C16 peptide (C16 representing 16 amino acid residues of the C-terminal of histatin-5; DSHAGYKRKFHEKHHSHRGY), which shows similar activity of histamine (Melino et al., 2006). Several studies indicate that ATCUN combined AMPs show more active in antimicrobial activity compared to ATCUN-free AMP. For instance, clavanin, a naturally occurring AMP is isolated from the hemocytes of the marine tunicate styela clava and has different forms, including clavanin-A, -B, -C, and -D. Among them, only clavanin-C contains ATCUN site that can speed up five-fold antimicrobial activity over ATCUN-free clavanin (Lee et al., 1997). Obviously, incorporation of ATCUN motif into AMPs gives extra mileage in mode of action of AMPs that facilitates to oxidatively damage the bacteria cell, unlike to simple electrostatic interaction between positive charge of AMPs and anionic bacterial membrane surface. The modes of action of AMP with and without ATCUN motif are different as shown in Figure 20. Thus, it motivates to develop and design ATCUN-coupled AMPs derivatives. For instance, the designed ATCUN-AMP derivatives (Cu-GGHGWRWYCRNH_2_ and Cu-GGHWRWYCRGGK-NH_2_) are more efficient compared to the parent AMPs (Joyner et al., 2013a, 2013b).

The antibacterial activity is also influenced by choice of amino acid in ATCUN motif. Three metal binding ATCUN motifs, GGH, VIH, and DAH are selected for study of antibacterial activity, in which Cu-DAH exhibits poor formation of ROS compared to others. The conjugation of Cu-GGH, Cu-VIH, Cu-DAH with AMP, the Cu-DAH-AMP is likely to be less active in antimicrobial activity than others two (Libardo et al., 2014). This approach is expanded to design various M-ATCUN-AMP derivatives (ATCUN: GGH, VIH, and DAH), where AMPs have different binding modes of action such as membrane-disrupting peptides, (anoplin; GLLKRIKTLL-NH_2_ sequence) (Ifrah et al., 2005; Konno et al., 2001) pro-apoptotic peptide (PAP; KLAKLAKKLAKLAK-NH_2_) (Javadpour et al., 1996; McGrath et al., 2013; Kim et al., 2011), and non-membrane-active peptide (sh-buforin; RAGLQFPVGRVHRLLRK-NH_2_) (Park et al., 1996, 2000). All derivatives are tested on antimicrobial activity against four different bacterial strains. Among them, Cu-VIH-anoplin derivative shows the highest antibacterial activity over others.

This approach is expanded to design an RTH-sh-buforin derivative possessing more positively charged on ATCUN motif that allows high binding affinity toward DNA over neutral ATCUN motif, VIH-sh-buforin. The switching from L- to D-amino acid, the antibacterial activity is enhanced, like nuclease activity (Libardo et al., 2015). To expand the utility of metal binding domain in AMPs, the Cu-ATCUN-OV-3 (OV-3; ovispirin-3 from the cathelicidin family) derivative shows higher rate of antimicrobial activity toward wide range of bacteria relative to OV-3 alone. This derivative also shows the high level of membrane leakage and lipid peroxidation with respect to Cu-GGH or OV-3 alone. These results suggest that the Cu-GGH-OV-3 derivative oxidatively disrupts the bacterial cell membranes, resulting cell death, whereas OV-3 alone is able to permeabilization of cell membrane without lipid oxidation (Alexander et al., 2017). The other AMP, Sub5 is a linear synthetic peptide of bactenecin, which shows a broad range of antimicrobial activity against microbes (Mania et al., 2010). Similarly, Sub5 (RRWKIVVIRWRR-NH_2_) lacks the metal binding domain, ATCUN motif. Therefore, the addition of Cu-ATCUN motif (GGH and GGHG) into Sub5 to yield Cu-GGHRRWKIVVIRWRR-NH_2_ and Cu-GGHGRRWKIVVIRWRR-NH_2_ derivatives that significantly enhance (∼16 fold) the antimicrobial activity relative to parental Sub5 (Alexander et al., 2019).

Overall, the introduction of the M-ATCUN motif into AMP enhances the antimicrobial activity for a wide range of microbes relative to AMP alone. Therefore, M-ATCUN-AMP can be considered as a potential next-generation antibiotic for clinical treatments.

#### Modification of Amino Acid Residues

The redox-active amino acid residues, such as Met, Cys, His, Tyr, and Trp, are involved in different biological redox processes (Liu et al., 2014) that include (i) deleterious phenomena, as the one occurring under oxidative stress conditions, where the amino acids oxidation could modify (damage) the protein biological function (Stadtman 1993), and secondly, useful functions, as exemplified by stable or transient tyrosine radical or modified tyrosine radicals that are important for ribonucleotide reductase (Barlow et al., 1983), photosystem (Haumann et al., 1999), cytochrome *c* oxidase (Yu et al., 2012), and protein-protein cross-linkage (Zhang et al., 2016) and (ii) nitration of tyrosine residues in proteins represents a specific footprint of the formation of reactive nitrogen species (RNS) *in vivo* (Radi 2013). However, above discussion described that Ni-ATCUN derivatives are actively cleavage the DNA/RNA through Ni^II^/Ni^III^ redox couple. This redox chemistry of Ni-ATCUN derivative has been applied to other performances including protein-protein cross-linking (intermolecular tyrosine-tyrosine cross-linking) (Horowitz et al., 2012) and tyrosine nitration (Maiti et al., 2019).

##### Protein-Protein Cross-Linking

**Figure 21:**
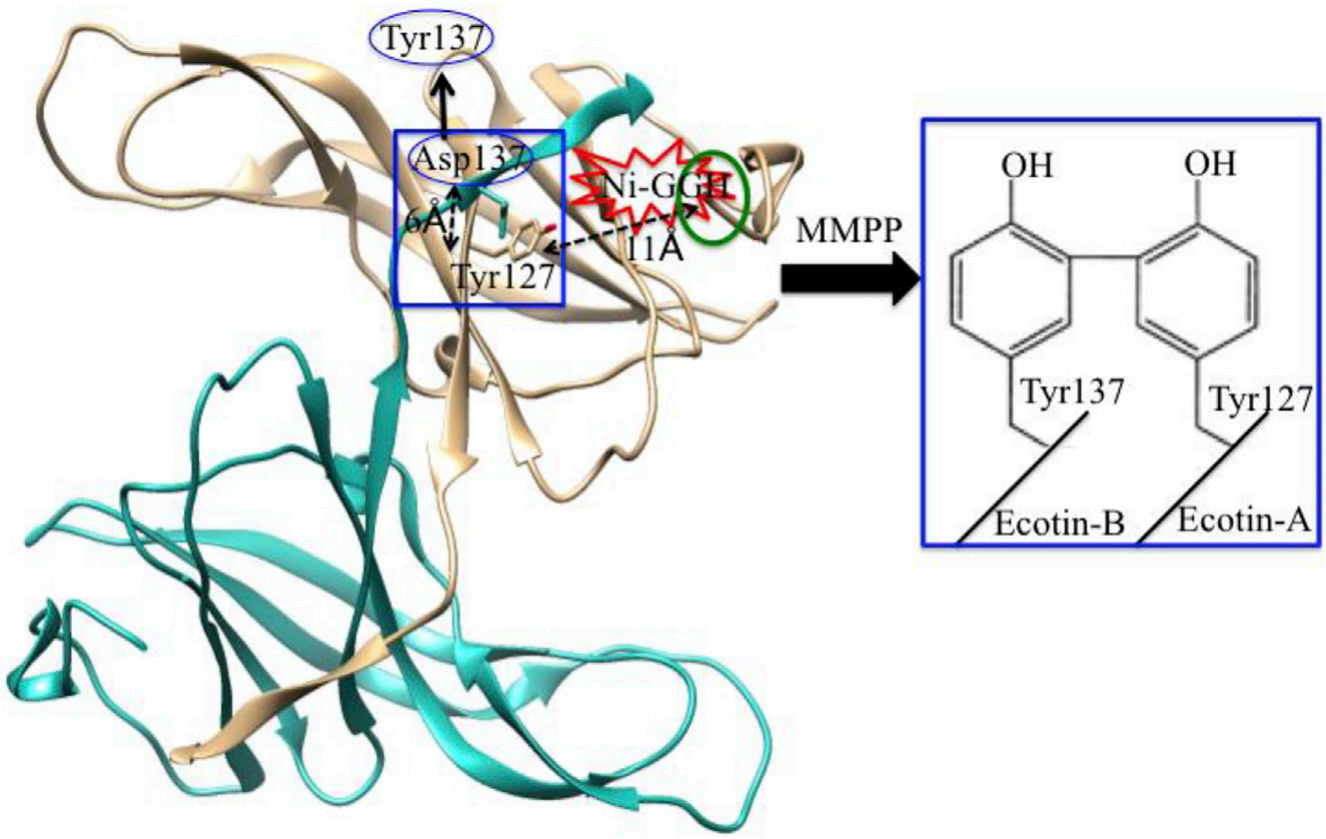
The Formation of Tyrosine Cross-Linking Product by Ni-ATCUN in GGH-Ecotin (D137Y) The ecotin structure is derived from the PBD: 1ECZ (Shin et al., 1996). The Ni-ATCUN (red star) is assumed to bind N-terminus (green circle) of ecotin-A (gray) and distance from Tyr_127_ is ~11 Å. The distance between mutant Asp_137_→Tyr_137_ in ecotin-B (cyano) and wild Tyr_127_ in ecotin-A (gray) is 6 Å (highlighted blue box). MMPP = monoperoxyphthalate.

The protein-protein cross-linking is a useful method to study for probing the multi-protein architectures, including RNase (Gill et al., 1997), bacteriophage Qb (Meunier et al., 2004), adeno-associated viral (AAV) capsids (Horowitz et al., 2012; Campbell et al., 1998; Fancy and Kodadek, 1999). This type of cross-linking process has been also employed in many fields such as biochemical and biomedical research, food processing, tissue engineering (Meunier et al., 2004; Brown et al., 1995). One useful method of cross-linking is oxidation of tyrosine residues to yield di-tyrosine product. Oxidative tyrosine cross-linking is catalyzed by various metalloenzymes (Michon et al., 1997; Sánchez-Ferrer et al., 1995) or metal-binding peptides (Gill et al., 1997; Brown et al., 1995; Fancy and Kodadek, 1998). For example, Ni-ATCUN with oxidant is a useful protein cross-linking reagent for the study of macromolecular protein assemblies. Brown et al. reported a useful cross-linking reagent, Ni-GGH and MMPP (monoperoxyphthalic acid) that efficiently produced protein-protein cross-linking product, Tyr-Tyr unit through tyrosyl radical pathway (Brown et al., 1995). To expand the usage of this reagent, Ni-GGH is fused with ecotin protein, yielding Ni-GGH-ecotin that oxidatively produces cross-linking product, homodimeric-ecotin as serine protease inhibitor, and it is found in the periplasm of *E. coli* (Chung et al., 1983; McGrath et al., 1991). To confirm the formation of bityrosyl cross-linking product, Person et al. used a model protein, mutage GGH-ecotin (D137Y) that enhanced the 4-fold cross-linking product formation in comparison with wild-type (Person et al., 2001). The Ni-GGH catalytic site in ecotin (ecotin-A) oxidizes the close proximity Tyr_127_ to yield tyrosine radical that finally reacts with close proximity engineered Tyr_137_ residue of another ecotin (ecotin-B) molecule to yield cross-linking product, ecotin-A-Tyr_127_-Tyr_137_-ecotin-B (Figure 21).

This tyrosine-tyrosine stitching reagent is also effectively used to investigate the structure of virus system where inter-subunit of virus is covalently stitched by the oxidation of adjacent tyrosine residues exclusively (Meunier et al., 2004). The utility of tyrosine cross-linking is expanded toward viral capsid for structural analysis of AAV during infection. Tyr-cross-linked modified AAV showed lower transduction efficiency compared to unmodified capsids (Horowitz et al., 2012). In addition, tyrosine-cross linking product is also found in insulin (Correia et al., 2012). This versatility of di-tyrosine cross-linking reagent is a useful candidate in the development of new biomaterial production for potential implication in medical research in coming years (Kodadek 2002; Petrik 2001; MacBeath and Schreiber, 2000).

##### Tyrosine Nitration

**Figure 22:**
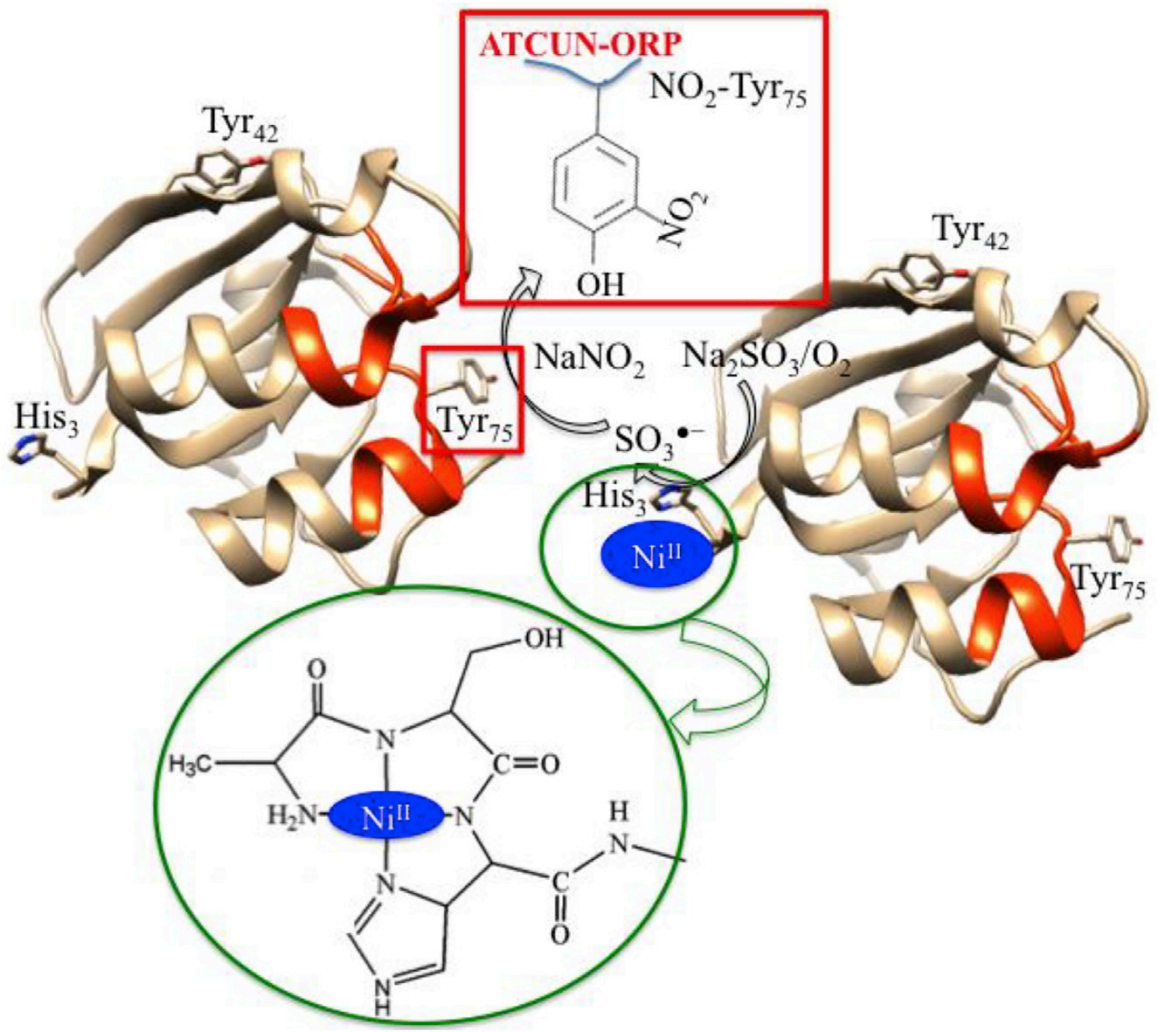
Nitration of Tyrosine in ORP by Ni-ATCUN in Presence of Na_2_SO_3_/NaNO_2_/O_2_ (Modified from Maiti et al., 2019) The drawing was adapted from the crystal structure of apo-ATCUN-ORP [PDB: 2wfb, Najmudin et al., 2009].

The formation of 3-nitrotyrosine *in vivo* represents a specific footprint of RNS that is often related to several pathophysiological disorders, such as Alzheimer's and Parkinson's diseases (Reyes et al., 2011). The 3-nitrotyrosine is formed in multiple pathways involving variable RNS such as peroxynitrite (ONOO^−^) or nitrogen dioxide (⋅NO_2_) and promoted by transition metal ions (Fe, Cu, and Mn) or metalloproteins (Ferrer-Sueta et al., 2018). Very recently, Maiti et al. have presented an alternative sulfur metabolism pathway formation of 3-nitrotyrosine, where Ni^II^-ATCUN motif is conjugated with ORP to yield Ni^II^-ATCUN-ORP that catalyzes the nitration of tyrosine residue in ORP in the presence of nitrite and sulfite *in vitro* under biological conditions (Figure 22) (Maiti et al., 2019). To our knowledge, the 3-nitrotyrosine formation by Ni^II^-ATCUN in the presence of the NO_2_^-^/SO_3_^2−^/O_2_ system over peroxynitrite or H_2_O_2_/NO_2_ system is the first report. This study represents an alternative pathway formation of 3-nitrotyrosine, which may influence the progression of disease states *in vivo* involving sulfur metabolism.

#### Small Molecule Activation

Currently, more attention is given on small molecules activation for current sustainability issues (Meyer and Tolman, 2015; Gutsulyak et al., 2013; Hong et al., 2017). Nature is the master of small molecule activation and designs variety of machines such as nitrogen fixation (Hoffman et al., 2014), and photosynthesis (Yano and Yachandra, 2014), involving one-, two-, or multi-electrons and -protons process. Every transformation has great significance for sustainable energy. Inspired by nature, Chemists have designed peptide-based-metal catalysts that are an emerging catalyst for small molecule activation (Yu et al., 2014; Jones et al., 2007). The redox active M-ATCUN derivatives are primarily studied on biomolecules cleavage/modification. These catalytic systems also serve as small molecule activations, which include water oxidation, hydrogen evolution, and nitrite to ammonium (see below).

##### Water Oxidation

Water oxidation is a thermodynamically unfavorable chemical process, where oxygen evolution from water involves the loss of four protons and four electrons (2H_2_O = O_2_ + 4H^+^ + 4e^−^, E^0^ = 1.23 V vs. NHE). This challenging chemical process is performed by the Nature, known as the terminal step of photosystem II (PSII) (Hurst 2010; Liu and Wang, 2012; McEvoy and Brudvig, 2006). The interest is basically on the formation of protons and electrons (production of H_2_ energy) instead of oxygen evolution, which has been considered as the water splitting process. In this field, the designed various metal-complexes such as peptide based metal complexes have progressed rapidly. For instance, Meyer et al. have reported a Cu-peptide complex (Cu-GGGG; non-ATCUN motif), a square-based with coordinated four N donor ligand like Cu-ATCUN system, which shows good water oxidation by electrochemically with turnover frequency, 33 s^−1^ but operates at high pH ∼ 11 and high oxidation potential at 1.32 V vs. NHE (Zhang et al., 2013).

Switching to Cu-ATCUN, water oxidation is also observed. Malinka et al. reported two mononuclear Cu^II^ complexes with Dap-based peptides, G(Dap)GG-NH_2_ (3G) and G(Dap)GH-NH_2_ (2GH) (Dap = L-2,3-diaminopropionic acid) that catalyzed water oxidation. In these derivatives, the 2GH facilitates more proton-coupled electrons transfer (PCETs) over 3G derivative in the course of water oxidation, and also improves the turn over frequency (TOF), 53 s^−1^ (23 s^−1^ for 3G) at pH 11 with redox potential at 1.32 V vs. NHE. This PCETs reaction is facilitated by the presence of His residue (Pap et al., 2015; Szyrwiel et al., 2017). Deng et al. reported a series of ATCUN motifs, including neutral as well as positively or negatively charged tri-peptides (GGH, KGH, RTH, DAH), tetra-peptides (GGHG, KGHG, RTHD, DAHF), and C-terminally aminated tripeptides (GGH-NH_2_, KGH-NH_2_) for water oxidation. Interestingly, Cu^II^-tripeptide derivatives exhibit higher TOF for water oxidation than Cu-tetrapeptide, as well as aminated tripeptides derivatives. Unlike other Cu^II^-peptide derivatives, the Cu-ATCUN derivatives operate water oxidation at neutral pH and at lower oxidation potential (Szyrwiel et al., 2017).

##### Hydrogen Evolution

Nature utilizes an efficient enzyme, Hydrogenases, that cleave heterolytically the hydrogen molecule in a reversible mode (Maiti et al., 2017a; Lubitz et al., 2014). H_2_ production (an alternative source of fossil fuel) from the reduction of aqueous proton is one of the potential sustainable energy routes. Numerous metal-complexes as well as peptide-based metal complexes are reported for H_2_ production. Among them, the most promising artificial catalysts of hydrogen evolution are mainly cobalt complexes, which exhibit high turnover numbers (TONs), ∼5 × 10^4^ (Sun et al., 2011; Kleingardner et al., 2014; Eckenhoff et al., 2013).

Surprisingly, water-soluble Co-ATCUN represents a new class of hydrogen evolution catalyst and has a similar coordination environment as reported Co-catalysts. Bren et al. reported a small Co-tripeptide model (Co-GGH) complex that produced H_2_ from proton with 275 TON at nearly neutral pH and at ∼600 mV overpotential (Kandemir et al., 2016). This overpotential value is relatively lower than other water-soluble cobalt porphyrin-peptide (∼850 mV) at pH 7 (Kleingardner et al., 2014) but higher than the popular H_2_ evolution Co-catalyst, “cobaloxime”, which has lower over-potential (∼250–300 mV) at nearly neutral pH (Jacques et al., 2009; Bacchi et al., 2014). This interesting feature of Co-GGH derivative is observed due to the presence of adjacent terminal amino group to Co-center, which plays as a proton shuttle during the catalysis process. The deportonation directly connects with pKa value that may influence the overpotential. So, this pKa value can be modulated by modification of ATCUN peptide (Neupane et al., 2013, 2014). At pH 6.5, the hydrogen evolution by Co-GGH derivative is higher, but the stability of the complex is lower, which influences the activity. Recently, Bren et al. have also reported a light-driven reaction, where a CoGGH derivative produces hydrogen from aqueous protons in water at near neutral pH by using a photosensitizer ([Ru(bpy)_3_]^2+^) and an electron donor candidate (ascorbate) (Chakraborty et al., 2019). The turnover number of hydrogen production is up to 2200 than previous system and this catalyst also shows exceptional longevity (more than 48 hr).

In this regard, the modification of GGH peptide in Co-GGH can be an attractive and promising hydrogen evolution candidate that may hold the current energy issues.

##### Reduction of Nitrite to Ammonium

The reduction of nitrite to ammonium is a multi-electron and multi-proton chemical task (Rosca et al., 2009). The six electrons reduction of nitrite to ammonium is one of the key steps in the biological nitrogen cycle that is catalyzed by cytochrome *c* nitrite reductase through dissimilatory pathway (Einsle et al., 1999) or the siroheme-containing nitrite reductase through assimilatory pathway (Crane et al., 1995). Important progress of bio-inspired small synthetic model systems for nitrite reductase has been developed but most of these catalysts perform partial reduction of nitrite to nitric oxide (1 electron game) (Timmons and Symes, 2015; Moore and Szymczak, 2015; Hematian et al., 2012) and/or nitrous oxide (2 electrons game) (Uyeda and Peters, 2013). In 2018, Guo et al. reported a Co-GGH model that performed selective reduction of nitrite to ammonium (six-electron and eight-proton game) by electrochemically in aqueous buffer at pH ∼7 (Guo et al., 2018). This result indicates that a simple Co-GGH model serves as multi-electrons and multi-protons functional activity of cytochrome *c* nitrite reductase and siroheme-containing nitrite reductase (Rosca et al., 2009; Einsle et al., 1999; Crane et al., 1995). NiGGH and CuGGH derivatives are also tested under the same conditions but none of them generates ammonia from nitrite.

The field of small molecule activation by M-ATCUN is still in an early development stage. However, the results obtained, so far, are promising and motivating for the search of new chemical performances.

## Conclusion and Future Challenges

This review aimed to shed light on wide and selective applications of designed ATCUN-derivatives involved in multifunctional aspects such as: spectroscopic probes, sensors, target-based catalytic metallodrugs, biomolecules stitching, inhibition of Aβ-peptide aggregation, and small molecules activation.

The redox chemistry of M-ATCUNs still rises questions but not yet fully clarified, particularly the redox couples, Cu^II^/Cu^I^ and Cu^III^/Cu^II^ involved in Cu-ATCUN derivatives during production of hydroxyl radical under ascorbate/H_2_O_2_ system. The Cu^II^/Cu^I^ and Cu^III^/Cu^II^ redox couples with high redox potential, as well as inherent coordination preferences by Cu^I^ and Cu^II^ make still question on redox mechanism as well as catalytic efficiency. The one step progress could be the incorporation of bis-His motif nearby ATCUN (bind site of Cu^I^), but the problem still remains due to Cu migration from ATCUN to bis-His motif upon ascorbate reduction that may decrease the catalytic efficiency. So, a design strategy needs to be developed, where Cu^II^ and Cu^I^ play redox chemistry in the same framework for enhancing redox activity. The redox chemistry of Cu-ATCUN-substrate adduct (substrate: ascorbate/H_2_O_2_/O_2_) can modulate the redox potential, an aspect to be accounted.

However, the design of hybrid M-ATCUN-recognition derivatives is a useful concept for therapeutic intervention. The interest is to drive the ATCUN derivatives toward cellular targets mainly cytosol and nucleus, and these should be reached to the target position in an active form. In cellular assays, like cis-Pt (Lasorsa et al., 2019) and TTM drug (Maiti and Moura, 2020), ATCUN drugs may also interact with intercellular proteins, mainly, copper trafficking proteins such as metallothioneins (MTs; as a biological strong Cu^I^-chelators) and glutathione (GSH; as a biological copper reducer) (Boal and Rosenzweig, 2009). Recently, Santoro *et al.* have reported that Cu^I^ and Cu^II^ oxidation states of Cu-catalysts during O_2_ activation under ascorbate are unstable against GSH/MT system (Santoro et al., 2020). Therefore, intercellular Cu-trafficking proteins are a challenge for the design of Cu-ATCUN complexes as oxidative cleavage catalysts in cells. The other analog, Ni-ATCUN is also a promising drug but problems arise in cellular compartments due to its thiophilic nature.

Recent studies have shown that the novel severe acute respiratory syndrome coronavirus 2, (SARS-CoV-2) directly binds to human ACE2 prior to entry into host cell, causing viral infection (Wrapp et al., 2020; Yan et al., 2020; Maiti 2020). ATCUN motif can be conjugated with a recognition domain (ACE2) of SARS-CoV-2 to yield ATCUN-recognition-domain, which can selectively bind and oxidatively modify the amino acid residues (such as tyrosine) of spike protein and hence it would be a promising drug/therapy against SARS-CoV-2 in future. However, more screenings are required for the development of more efficient drugs in clinical practice.

The M-ATCUN derivatives are also employed for novel applications, which include water oxidation, hydrogen evolution and ammonium synthesis. In these studies, the small M-ATCUN motifs (GGH) were used wherein the second-sphere of protein scaffold was fully absent, which could enhance the catalytic activity. This ATCUN-motif has the ability for second-sphere interactions with the metal active site via carboxylate moiety or N-terminus. In the past decade, the light-driven catalytic reactions have been significantly developing. The conjugation of photosensitizer into ATCUN motif can produce a bioinspired system that can operate the chemical reaction by light as a renewable energy source. Recently, Bren et al. reported a light-driven hydrogen production by using a photosensitizer ([Ru(bpy)_3_]^2+^) (Chakraborty et al., 2019). In addition, incorporation of redox active metal ions (such as Ru, Au, and Mn) into the designed ATCUN motif can perform a wide range of vital and challenging chemical transformations. Therefore, there are many challenges in small molecule activation that can be overcome by incorporation of photosensitizer and/or redox active metal ions into the designed ATCUN motif.

In biology, HSA possessing ATCUN site plays a key role for copper transport in human blood but the mechanism of copper transport and extracellular copper delivery by ATCUN tag in HSA are still unclear *in vivo*. Recently, Bal *et al.* have demonstrated that a transient two N-coordinated Cu-ATCUN complex is formed within very very short life time (t_1/2_–100 ms) during the initial stage of the interaction between Cu^II^ and ATCUN site, which may be able to maintain the Cu^II^/Cu^I^ redox couple and extracellular copper transport/delivery (Kotuniak et al., 2020). As ATCUN motif has high affinity for Cu^II^, it can recruitment Cu^II^ ions from the labile copper pool, which has clinically significance as potential markers of copper-related pathologies. Therefore, measurement of this labile copper pool in biological samples is a significant interest. Several ATCUN-conjugate fluorescent sensors are reported, which are mainly based on turn-off Cu^II^ sensors but the rational design of turn-on Cu^II^ sensors is still very challenging in biological medium.

Because of small size, the Cu^II^-ATCUN is likely to introduce into protein as paramagnetic NMR tag for understanding the structure/function relationship of many biomolecules. The paramagnetic relaxation enhancement can be improved by incorporation of lanthanoid ions such as Gd^III^ or conjugation of Gd^III^ chelates into ATCUN motif.

The redox silent (or high stable) M-ATCUN derivative is another important issue *in vivo* applications, such as spectroscopic probe, bio-imaging, copper chelation therapy. Thus, high stability and high copper binding affinity of a designed motif can be useful for copper chelation therapy by forming a stable M-ATCUN derivative that significantly prevents the hydroxyl radical formation as well as Aβ-peptide aggregation.

Compared with other metal-motif, the ATCUN motif is a naturally occurring small peptide, less toxic, soluble in biological buffer, and high affinity for Cu^II^. It can be rationally designed by choice of amino acids that can tune-up the catalytic activity and redox inertness. Furthermore, the ATCUN motif can be conjugated with a recognition domain that provides target specificity and drug delivery, with reduced toxicity and minimum side effects. Bio-conjugation is not limited to the ATCUN motif. For instance, peptide is conjugated with organometallic groups such as metallocenes (Chantson et al., 2006), hence representing another popular strategy to modulate activity and cellular uptake. However, the major challenge is intracellular delivery to cellular targets. So, ATCUN conjugation would be a promising strategy in future. The other metal-complexes such as [Fe(EDTA)]^2−^ (EDTA = ethylenediaminetetraacetic acid) (Pogozelski et al., 1995), [Cu(phen)_2_]^+^ (phen = 1,10-phen-anthroline) (Sigman 1986), metalloporphyrins (Mestre et al., 1996), and metal-salen (Czlapinski and Sheppard, 2004) are well-known metallonucleases, but in practical application, these are less selective. Therefore, ATCUN motifs can be introduced over other metal complexes. However, rational design is an ultimate step to overcome the critical challenges encountered in various biological and chemical issues.

## References

[bib1] Aboul-ela F., Varani G. (1998). Recognition of HIV-1 TAR RNA by tat protein and tat-derived peptides. J. Mol. Struct..

[bib2] Agbale C.M., Cardoso M.H., Galyuon I.K., Franco O.L. (2016). Designing metallodrugs with nuclease and protease activity. Metallomics.

[bib3] Alexander J.L., Yu Z., Cowan J.A. (2017). Amino terminal copper and nickel binding motif derivatives of ovispirin-3 display increased antimicrobial activity via lipid oxidation. J. Med. Chem..

[bib4] Alexander J.L., Thompson Z., Yu Z., Cowan J.A. (2018). Antimicrobial metallopeptides. ACS Chem. Biol..

[bib5] Alexander J.L., Thompson Z., Yu Z., Cowan J.A. (2019). Cu-ATCUN derivatives of Sub5 exhibit enhanced antimicrobial activity via multiple modes of action. ACS. Chem. Biol..

[bib6] Al-Harthi S., Lachowicz J.I., Nowakowski M.E., Jaremko M., Jaremko Ł. (2019). Towards the functional high-resolution coordination chemistry of blood plasma human serum albumin. J. Inorg. Biochem..

[bib7] Bacchi M., Berggren G., Niklas J., Veinberg E., Mara M.W., Shelby M.L., Poluektov O.G., Chen L.X., Tiede D.M., Cavazza C. (2014). Cobaloxime-based artificial hydrogenases. Inorg. Chem..

[bib8] Bal W., Chmurny G.N., Hilton B.D., Sadler P.J., Tucker A. (1996). Axial hydrophobic fence in highly-stable Ni(II) complex of des-angiotensinogen N-terminal peptide. J. Am. Chem. Soc..

[bib9] Bal W., Sokołowska M., Kurowska E., Faller P. (2013). Binding of transition metal ions to albumin: sites, affinities and rates. Biochim. Biophys. Acta.

[bib10] Bal W., Jezowska-Bojczuk M., Kasprzak K.S. (1997). Binding of nickel(II) and copper(II) to the N-terminal sequence of human protamine HP2. Chem. Res. Toxicol..

[bib11] Balayssac S., Bertini I., Bhaumik A., Lelli M., Luchinat C. (2008). Paramagnetic shifts in solid state NMR of proteins to elicit structural information. Proc. Natl. Acad. Sci. USA.

[bib12] Banci L., Bertini I., Eltis L.D., Felli I.C., Kastrau D.H., Luchinat C., Piccioli M., Pierattelli R., Smith M. (1994). The three-dimensional structure in solution of the paramagnetic high-potential iron-sulfur protein I from Ectothiorhodospira halophila through nuclear magnetic resonance. Eur. J. Biochem..

[bib13] Barlow T., Eliasson R., Platz A., Reichard P., Sjöberg B.M. (1983). Enzymic modification of a tyrosine residue to a stable free radical in ribonucleotide reductase. Proc. Natl. Acad. Sci. USA.

[bib14] Barthelmes K., Reynolds A.M., Peisach E., Jonker H.R.A., DeNunzio N.J., Allen K.N., Imperiali B., Schwalbe H. (2011). Engineering encodable lanthanide-binding tags into loop regions of proteins. J. Am. Chem. Soc..

[bib15] Battiste J.L., Mao H., Rao N.S., Tan R., Muhandiram D.R., Kay L.E., Frankel A.D., Williamson J.R. (1996). α helix-RNA major groove recognition in an HIV-1 Rev peptide-RRE RNA complex. Science.

[bib16] Bazan J.F., Fletterick R.J. (1989). Detection of a trypsin-like serine protease domain in flaviviruses and pestiviruses. Virology.

[bib17] Beechem J.M., Brand L. (1985). Time-resolved fluorescence of proteins. Annu. Rev. Biochem..

[bib18] Best S.L., Chattopadhyay T.K., Djuran M.I., Palmer R.A., Sadler P.J., Sóvágó I., Varnagy K. (1997). Gold(III) and palladium(II) complexes of glycylglycyl-L-histidine: crystal structures of [Au^III^(Gly-Gly-L-His-H_-2_)]Cl·H_2_O and [Pd^II^(Gly-Gly-L-His-H_-2_)]·1.5H_2_O and HisεNH deprotonation. Dalton Trans..

[bib19] Boal A.K., Rosenzweig A.C. (2009). Structural biology of copper trafficking. Chem. Rev..

[bib20] Boeneman K., Mei B.C., Dennis A.M., Bao G., Deschamps J.R., Mattoussi H., Medintz I.L. (2009). Sensing caspase 3 activity with quantum Dot−Fluorescent protein assemblies. J. Am. Chem. Soc..

[bib21] Borsook H., Keighley G. (1933). Oxidation-reduction potential of ascorbic acid (vitamin C). Proc. Natl. Acad. Sci. USA.

[bib22] Bortolus M., Bisaglia M., Zoleo A., Fittipaldi M., Benfatto M., Bubacco L., Maniero A.L. (2010). One-pot synthesis of stable NIR tetracene diimides via double cross-coupling. J. Am. Chem. Soc..

[bib23] Bossak-Ahmad K., Fraczyk T., Bal W., Drew S. (2020). The sub-picomolar Cu^2+^ dissociation constant of human serum albumin. ChemBioChem.

[bib24] Bossak K., Drew S.C., Stefaniak E., Płonka D., Bonnac A., Bal W. (2018). The Cu(II) affinity of the N-terminus of human copper transporter CTR1: comparison of human and mouse sequences. J. Inorg. Biochem..

[bib25] Bossu F.P., Chellappa K.L., Margerum D.W. (1977). Ligand effects on the thermodynamic stabilization of copper(III)-peptide complexes. J. Am. Chem. Soc..

[bib26] Bradford S., Cowan J.A. (2012). Catalytic metallodrugs targeting HCVIRESRNA. Chem. Commun..

[bib27] Bradford S.S., Ross M.J., Fidai I., Cowan J.A. (2014). Insight into the recognition, binding, and reactivity of catalytic metallodrugs targeting stem loop IIb of hepatitis C IRES RNA. ChemMedChem.

[bib28] Brath U., Swamy S.I., Veiga A.X., Tung C.C., Petegem F.V., Erdélyi M. (2015). Paramagnetic ligand tagging to identify protein binding sites. J. Am. Chem. Soc..

[bib29] Brandt C., van Eldik R. (1995). Transition metal-catalyzed oxidation of sulfur(IV) oxides. Atmospheric-relevant Process. Mech. Chem. Rev..

[bib30] Brittain I.J., Huang X., Long E.C. (1998). Selective recognition and cleavage of RNA loop structures by Ni(II)·Xaa-Gly-His metallopeptides. Biochemistry.

[bib31] Brogden K.A. (2005). Antimicrobial peptides: pore formers or metabolic inhibitors in bacteria?. Nat. Rev. Microbiol..

[bib32] Brown J.M., Wilson W.R. (2004). Exploiting tumour hypoxia in cancer treatment. Nat. Rev. Cancer.

[bib33] Brown K.C., Yang S.H., Kodadek T. (1995). Highly specific oxidative crosslinking of proteins mediated by a nickel-peptide complex. Biochemistry.

[bib34] Brown K.C., Yu Z., Burlingame A.L., Craik C.S. (1998). Determining Protein−Protein interactions by oxidative cross-linking of a glycine-glycine-histidine fusion protein. Biochemistry.

[bib35] Burger K.. (1990). Biocoordination Chemistry. Coordination Chemistry in Biologically Active Systems. Inorganic Chemistry.

[bib36] Burrows C.J., Muller J.G. (1998). Oxidative Nucleobase modifications leading to strand scission. Chem. Rev..

[bib37] Camerman N., Camerman A., Sarkar B. (1976). Molecular design to mimic the copper(II) transport site of human albumin. The crystal and molecular structure of copper(II)-glycylglycyl-L-histidine-N-methyl amide monoaquo complex. Can. J. Chem..

[bib38] Campbell L.A., Kodadek T., Brown K.C. (1998). Protein cross-linking mediated by metalloporphyrins. Bioorg. Med. Chem..

[bib39] Campbell N.H., Parkinson G.N., Reszka A.P., Neidle S. (2008). Structural basis of DNA quadruplex recognition by an acridine drug. J. Am. Chem. Soc..

[bib40] Carter D.C., Ho J.X. (1994). Structure of serum albumin. Adv. Protein Chem..

[bib41] Cerofolini L., Staderini T., Giuntini S., Ravera E., Fragai M., Parigi G., Pierattelli R., Luchinat C. (2018). Long-range paramagnetic NMR data can provide a closer look on metal coordination in metalloproteins. J. Biol. Inorg. Chem..

[bib42] Chakravarty S., Kannan K.K. (1994). Drug-protein interactions. Refined structures of three sulfonamide drug complexes of human carbonic anhydrase I enzyme. J. Mol. Biol..

[bib43] Chakraborty S., Edwards E.H., Kandemir B., Bren K.L. (2019). Photochemical hydrogen evolution from neutral water with a cobalt metallopeptide catalyst. Inorg. Chem..

[bib44] Chappell K.J., Stoermer M.J., Fairlie D.P., Young P.R. (2008). west Nile virus NS2B/NS3 protease as an antiviral target. Curr. Med. Chem..

[bib45] Chantson J.T., Falzacappa M.V.V., Crovella S., Metzler-Nolte N. (2006). Solid-phase synthesis, characterization, and antibacterial activities of metallocene–peptide bioconjugates. ChemMedChem.

[bib46] Cheignon C., Jones M., Atrián-Blasco E., Kieffer I., Faller P., Collin F., Hureau C. (2017). Identification of key structural features of the elusive Cu–Aβ complex that generates ROS in Alzheimer’s disease. Chem. Sci..

[bib47] Choi Y.A., Keem J.O., Kim C.Y., Yoon H.R., Heo W.D., Chung B.H., Jung Y. (2015). A novel copper-chelating strategy for fluorescent proteins to image dynamic copper fluctuations on live cell surfaces. Chem. Sci..

[bib48] Chung C.H., Ives H.E., Almeda S., Goldberg A.L. (1983). Purification from Escherichia coli of a periplasmic protein that is a potent inhibitor of pancreatic proteases. J. Biol. Chem..

[bib49] Clore G.M., Iwahara J. (2009). Theory, practice, and applications of paramagnetic relaxation enhancement for the characterization of transient low-population states of biological macromolecules and their complexes. Chem. Rev..

[bib50] Conklin S.E., Bridgman E.C., Su Q., Riggs-Gelasco P., Haas K.L., Franz K.J. (2017). Specific histidine residues confer histatin peptides with copper-dependent activity against Candida albicans. Biochemistry.

[bib51] Correia M., Neves-Petersen M.T., Jeppesen P.B., Gregersen S., Petersen S.B. (2012). UV-light exposure of insulin: pharmaceutical implications upon covalent insulin dityrosine dimerization and disulphide bond photolysis. PLoS One.

[bib52] Cossart P., Jonquières R. (2000). Sortase, a universal target for therapeutic agents against Gram-positive bacteria?. Proc. Natl. Acad. Sci. USA.

[bib53] Cowan J.A. (2001). Chemical nucleases. Curr. Opin. Chem. Biol..

[bib54] Crackower M.A., Sarao R., Oudit G.Y., Yagil C., Kozieradzki I., Scanga S.E., Oliveira-dos-Santos A.J., da Costa J., Zhang L., Pei Y. (2002). Angiotensin-converting enzyme 2 is an essential regulator of heart function. Nature.

[bib55] Crane B.R., Siegel L.M., Getzoff E.D. (1995). Sulfite reductase structure at 1.6 Å: evolution and catalysis for reduction of inorganic anions. Science.

[bib56] Crich D., Mo X.-S. (1997). Nucleotide C3‘,4‘-radical cations and the effect of a 2‘-oxygen substituent. The DNA/RNA paradox. J. Am. Chem. Soc..

[bib57] Cuenca F., Greciano O., Gunaratnam M., Haider S., Munnur D., Nanjunda R., Wilson W.D., Neidle S. (2008). Tri- and tetra-substituted naphthalene diimides as potent G-quadruplex ligands. Biorg. Med. Chem. Lett..

[bib58] Czlapinski J.L., Sheppard T.L. (2004). Site-specific oxidative cleavage of DNA by metallosalen–DNA conjugates. Chem. Commun..

[bib59] Dawson R.M., Liu C.Q. (2009). Cathelicidin peptide SMAP-29: comprehensive review of its properties and potential as a novel class of antibiotics. Drug Dev. Res..

[bib60] Deng D., Liu L., Bu Y., Liu X., Wang X., Zhang B. (2018). Electrochemical sensing devices using ATCUN-Cu(II) complexes as electrocatalysts for water oxidation. Sensor. Actuat. B Chem..

[bib61] Deng D., Hao Y., Xue J., Liu X., Xu X., Liu L. (2019). A colorimetric enzyme-linked immunosorbent assay with CuO nanoparticles as signal labels based on the growth of gold nanoparticles in situ. Nanomaterials.

[bib62] Deng D., Hao Y., Yang P., Xia N., Yu W., Liu X., Liu L. (2019). Single-labeled peptide substrates for detection of protease activity based on the inherent fluorescence quenching ability of Cu^2+^. Anal. Methods.

[bib63] Deshpande A., Mina E., Glabe C., Busciglio J. (2006). Different conformations of amyloid beta induce neurotoxicity by distinct mechanisms in human cortical neurons. J. Neurosci..

[bib64] Detmer C.A., Pamatong F.V., Bocarsly J.R. (1996). Nonrandom double strand cleavage of DNA by a monofunctional metal Complex:  mechanistic studies. Inorg. Chem..

[bib65] Donaldson L.W., Skrynnikov N.R., Choy W.-Y., Muhandiram D.R., Sarkar B., Forman-Kay J.D., Kay L.E. (2001). Structural characterization of proteins with an attached ATCUN motif by paramagnetic relaxation enhancement NMR spectroscopy. J. Am. Chem. Soc..

[bib66] Eckenhoff W.T., McNamara W.R., Du P., Eisenberg R. (2013). Cobalt complexes as artificial hydrogenases for the reductive side of water splitting. Biochim. Biophys. Acta Bioenerg..

[bib67] Einsle O., Messerschmidt A., Stach P., Bourenkov G.P., Bartunik H.D., Huber R., Kroneck P.M. (1999). Structure of cytochrome c nitrite reductase. Nature.

[bib68] Fabbrizzi L., Licchelli M., Pallavicini P., Perotti A., Taglietti A., Sacchi D. (1996). Fluorescent sensors for transition metals based on electron-transfer and energy-transfer mechanisms. Chem. Eur. J..

[bib69] Falcone E., Gonzalez P., Lorusso L., Sénèque O., Faller P., Raibaut L. (2020). A terbium(iii) luminescent ATCUN-based peptide sensor for selective and reversible detection of copper(ii) in biological media. Chem. Commun..

[bib70] Faller P. (2009). Copper and zinc binding to amyloid-beta: coordination, dynamics, aggregation, reactivity and metal-ion transfer. ChemBioChem.

[bib71] Fancy D.A., Kodadek T. (1998). A critical role for tyrosine residues in His6Ni-mediated protein cross-linking. Biochem. Biophys. Res. Commun..

[bib72] Fancy D.A., Kodadek T. (1999). Chemistry for the analysis of protein–protein interactions: rapid and efficient cross-linking triggered by long wavelength light. Proc. Natl. Acad. Sci. USA.

[bib73] Fang Y.Y., Ray B.D., Claussen C.A., Lipkowitz K.B., Long E.C. (2004). Ni(II)·Arg-Gly-His−DNA Interactions:  investigation into the basis for minor-groove binding and recognition. J. Am. Chem. Soc..

[bib74] Fang Y.Y., Claussen C.A., Lipkowitz K.B., Long E.C. (2006). Diastereoselective DNA cleavage recognition by Ni(II)⋅Gly-Gly-His-Derived metallopeptides. J. Am. Chem. Soc..

[bib75] Ferrer-Sueta G., Campolo N., Trujillo M., Bartesaghi S., Carballal S., Romero N., Alvarez B., Radi R. (2018). Biochemistry of peroxynitrite and protein tyrosine nitration. Chem. Rev..

[bib76] Fidai I., Hocharoen L., Bradford S., Wachnowsky C., Cowan J.A. (2014). Inactivation of sortase A mediated by metal ATCUN complexes. J. Biol. Inorg. Chem..

[bib77] Fleming R.E., Sly W.S. (2001). Hepcidin: a putative iron-regulatory hormone relevant to hereditary hemochromatosis and the anemia of chronic disease. Proc. Natl. Acad. Sci. USA.

[bib78] Folk D.S., Franz K.J. (2010). A prochelator activated by β-secretase inhibits Aβ aggregation and suppresses copper-induced reactive oxygen species formation. J. Am. Chem. Soc..

[bib79] Frankel A.D., Young J.A. (1998). HIV-1: fifteen proteins and an RNA. Annu. Rev. Biochem..

[bib80] Gaponenko V., Dvoretsky A., Walsby C., Hoffman B.M., Rosevear P.R. (2000). Calculation of z-coordinates and orientational restraints using a metal binding tag. Biochemistry.

[bib81] Gill G., Richter-Rusli A.A., Ghosh M., Burrows C.J., Rokita S.E. (1997). Nickel-dependent oxidative cross-linking of a protein. Chem. Res. Toxicol..

[bib82] Gokhale N.H., Bradford S., Cowan J.A. (2008). Catalytic inactivation of human carbonic anhydrase I by a Metallopeptide−Sulfonamide conjugate is mediated by oxidation of active site residues. J. Am. Chem. Soc..

[bib83] Gokhale N.H., Cowan J.A. (2005). Inactivation of human angiotensin converting enzyme by copper peptide complexes containing ATCUN motifs. Chem. Commun.

[bib84] Gonzalez P., Bossak K., Stefaniak E., Hureau C., Raibaut L., Bal W., Faller P. (2018). N-terminal Cu-binding motifs (Xxx-Zzz-His, xxx-his) and their derivatives: chemistry. Biol. Med. Appl. Chem. Eur. J..

[bib85] Goyal D., Shuaib S., Mann S., Goyal B. (2017). Rationally designed peptides and peptidomimetics as inhibitors of amyloid-β (Aβ) aggregation: potential therapeutics of Alzheimer’s disease. ACS Comb. Sci..

[bib86] Green B.J., Tesfai T.M., Xie Y., Margerum D.W. (2004). Oxidative self-decomposition of the nickel(III) complex of glycylglycyl-l-histidylglycine. Inorg. Chem..

[bib87] Guilloreau L., Combalbert S., Sournia-Saquet A., Mazarguil H., Faller P. (2007). Redox chemistry of copper-amyloid-beta: the generation of hydroxyl radical in the presence of ascorbate is linked to redox-potentials and aggregation state. ChemBioChem.

[bib88] Guo Y., Stroka J.R., Kandemir B., Dickerson C.E., Bren K.L. (2018). Cobalt metallopeptide electrocatalyst for the selective reduction of nitrite to ammonium. J. Am. Chem. Soc..

[bib89] Gutsulyak D.V., Piers W.E., Borau-Garcia J., Parvez M. (2013). Activation of water, ammonia, and other small molecules by PC_carbene_P nickel pincer complexes. J. Am. Chem. Soc..

[bib90] Haas K.L., Putterman A.B., White D.R., Thiele D.J., Franz K.J. (2011). Model peptides provide new insights into the role of histidine residues as potential ligands in human cellular copper acquisition via Ctr1. J. Am. Chem. Soc..

[bib91] Haass C., Selkoe D.J. (2007). Soluble protein oligomers in neurodegeneration: lessons from the Alzheimer's amyloid beta-peptide. Nat. Rev. Mol. Cell. Biol..

[bib92] Hagen W.R. (2006). EPR spectroscopy as a probe of metal centres in biological systems. Dalton Trans..

[bib93] Hancock R.E., Sahl H.G. (2006). Antimicrobial and host-defense peptides as new anti-infective therapeutic strategies. Nat. Biotechnol..

[bib94] Harford C., Sarkar B. (1995). Neuromedin C binds Cu(II) and Ni(II) via the ATCUN motif: implications for the CNS and cancer growth. Biochem. Biophys. Res. Commun..

[bib95] Harford C., Narindrasorasak S., Sarkar B. (1996). The designed protein M(II)-Gly-Lys-His-Fos(138−211) specifically cleaves the AP-1 binding site containing DNA. Biochemistry.

[bib96] Harford C., Sarkar B. (1997). Amino terminal Cu(II)- and Ni(II)-Binding (ATCUN) motif of proteins and Peptides:  metal binding, DNA cleavage, and other properties. Acc. Chem. Res..

[bib97] Haumann M., Mulkidjanian A., Junge W. (1999). Tyrosine-Z in oxygen-evolving photosystem II: a hydrogen-bonded tyrosinate. Biochemistry.

[bib98] Hawkins C.J., Martin J. (1983). Cobalt(III) complex of glycylglycyl-L-histidine: preparation, characterization, and conformation. Inorg. Chem..

[bib99] Hay R.W., Hassan M.M., You-Quan C. (1993). Kinetic and thermodynamic studies of the copper (II) and nickel(II) complexes of glycylglycyl-L-histidine. J. Inorg. Biochem..

[bib100] Hematian S., Siegler M.A., Karlin K.D. (2012). Heme/copper assembly mediated nitrite and nitric oxide interconversion. J. Am. Chem. Soc..

[bib101] Hocharoen L., Cowan J.A. (2009). Metallotherapeutics: Novel Strategies in Drug Design. Chem. Eur. J.

[bib102] Hocharoen L., Joyner J.C., Cowan J.A. (2013). N- versus C-domain selectivity of catalytic inactivation of human angiotensin converting enzyme by lisinopril-coupled transition metal chelates. J. Med. Chem..

[bib103] Hoffman B.M., Lukoyanov D., Yang Z.-Y., Dean D.R., Seefeldt L.C. (2014). Mechanism of nitrogen fixation by nitrogenase: the next stage. Chem. Rev..

[bib104] Holm R.H., Kennepohl P., Solomon E.I. (1996). Structural and functional aspects of metal sites in biology. Chem. Rev..

[bib105] Hong S., Lee Y.-M., Ray K., Nam W. (2017). Dioxygen activation chemistry by synthetic mononuclear nonheme iron, copper and chromium complexes. Coord. Chem. Rev..

[bib106] Horowitz E.D., Finn M.G., Asokan A. (2012). Tyrosine cross-linking reveals interfacial dynamics in adeno-associated viral capsids during infection. ACS Chem. Biol..

[bib107] Huang L., Kinnucan E., Wang G., Beaudenon S., Howley P.M., Huibregtse J.M., Pavletich N.P. (1999). Structure of an E6AP-UbcH7 complex: insights into ubiquitination by the E2-E3 enzyme cascade. Science.

[bib108] Hureau C., Eury H., Guillot R., Bijani C., Sayen S., Solari P.L., Guillon E., Faller P., Dorlet P. (2011). X-ray and solution structures of CuIIGHK and CuIIDAHK complexes: influence on their redox properties. Chem. Eur. J..

[bib109] Hureau C. (2012). Coordination of redox active metal ions to the amyloid precursor protein and to amyloid-β peptides involved in Alzheimer disease. Part 1: an overview. Coord. Chem. Rev..

[bib110] Hurst J.K. (2010). Pursuit of water oxidation catalysts for solar fuel production. Science.

[bib111] Ifrah D., Doisy X., Ryge T.S., Hansen P.R. (2005). Structure-activity relationship study of anoplin. J. Pept. Sci..

[bib112] Jacques P.A., Artero V., Pécaut J., Fontecave M. (2009). Cobalt and nickel diimine-dioxime complexes as molecular electrocatalysts for hydrogen evolution with low overvoltages. Proc. Natl. Acad. Sci. U S A.

[bib113] Javadpour M.M., Juban M.M., Lo W.-C.J., Bishop S.M., Alberty J.B., Cowell S.M., Becker C.L., McLaughlin M.L. (1996). De novo antimicrobial peptides with low mammalian cell toxicity. J. Med. Chem..

[bib114] Jensen M., Canning A., Chiha S., Bouquerel P., Pedersen J.T., Ostergaard J., Cuvillier O., Sasak I., Hureau C., Faller P. (2012). Inhibition of Cu-Amyloid-β by using bifunctional peptides with β-sheet breaker and chelator moieties. Chem. Eur. J..

[bib115] Jha N.N., Kumar R., Panigrahi R., Navalkar A., Ghosh D., Sahay S., Mondal M., Kumar A., Maji S.K. (2017). Comparison of α-synuclein fibril inhibition by four different amyloid inhibitors. ACS Chem. Neurosci..

[bib116] Jiang Q., Xiao N., Shi P., Zhu Y., Guo Z. (2007). Design of artificial metallonucleases with oxidative mechanism. Coord. Chem. Rev..

[bib117] Jin Y., Cowan J.A. (2005). DNA cleavage by Copper−ATCUN complexes. Factors influencing cleavage mechanism and linearization of dsDNA. J. Am. Chem. Soc..

[bib118] Jin Y., Cowan J.A. (2006). Targeted cleavage of HIV Rev response element RNA by metallopeptide complexes. J. Am. Chem. Soc..

[bib119] Jin Y., Lewis M.A., Gokhale N.H., Long E.C., Cowan J.A. (2007). Influence of stereochemistry and redox potentials on the single- and double-strand DNA cleavage efficiency of Cu(II)· and Ni(II)·Lys-Gly-His-Derived ATCUN metallopeptides. J. Am. Chem. Soc..

[bib120] Jin Y., Cowan J.A. (2007). Cellular activity of Rev response element RNA targeting metallopeptides. J. Biol. Inorg. Chem..

[bib121] Johnson G.D., Ahn K. (2000). Development of an internally quenched fluorescent substrate selective for endothelin-converting enzyme-1. Anal. Biochem..

[bib122] Jones A.K., Lichtenstein B.R., Dutta A., Gordon G., Dutton P.L. (2007). Synthetic Hydrogenases:  incorporation of an iron carbonyl thiolate into a designed peptide. J. Am. Chem. Soc..

[bib123] Joyner J.C., Reichfield J., Cowan J.A. (2011). Factors influencing the DNA nuclease activity of iron, cobalt, nickel, and copper chelates. J. Am. Chem. Soc..

[bib124] Joyner J.C., Cowan J.A. (2011). Targeted cleavage of HIV RRE RNA by rev-coupled transition metal chelates. J. Am. Chem. Soc..

[bib125] Joyner J.C., Hocharoen L., Cowan J.A. (2012). Targeted catalytic inactivation of angiotensin converting enzyme by lisinopril-coupled transition-metal chelates. J. Am. Chem. Soc..

[bib126] Joyner J.C., Keuper K.D., Cowan J.A. (2013). Kinetics and mechanisms of oxidative cleavage of HIV RRE RNA by Rev-coupled transition metal–chelates. Chem. Sci..

[bib127] Joyner J.C., Hodnick W.F., Cowan A.S., Tamuly D., Boyd R., Cowan J.A. (2013). Antimicrobial metallopeptides with broad nuclease and ribonuclease activity. Chem. Commun..

[bib128] Kalverda A.P., Salgado J., Dennison C., Canters G.W. (1996). Analysis of the paramagnetic copper(II) site of amicyanin by ^1^H NMR spectroscopy. Biochemistry.

[bib129] Kandemir B., Kubie L., Guo Y., Sheldon B., Bren K.L. (2016). Hydrogen evolution from water under aerobic conditions catalyzed by a cobalt ATCUN metallopeptide. Inorg. Chem..

[bib130] Kang C., Keller T.H., Luo D. (2017). Zika virus protease: an antiviral drug target. Trends Microbiol..

[bib131] Khadikar P.V., Sharma V., Karmarkar S., Supuran C.T. (2005). Bioorg. Med. Chem. Lett..

[bib132] Kieft J.S., Zhou K., Jubin R., Doudna J.A. (2001). Mechanism of ribosome recruitment by hepatitis C IRES RNA. RNA.

[bib133] Kim N.W., Piatyszek M.A., Prowse K.R., Harley C.B., West M.D., Ho P.L., Coviello G.M., Wright W.E., Weinrich S.L., Shay J.W. (1994). Specific association of human telomerase activity with immortal cells and cancer. Science.

[bib134] Kim H.Y., Kim S., Youn H., Chung J.K., Shin D.H., Lee K. (2011). The cell penetrating ability of the proapoptotic peptide, KLAKLAKKLAKLAK fused to the N-terminal protein transduction domain of translationally controlled tumor protein, MIIYRDLISH. Biomaterials.

[bib135] Kimoto E., Tanaka H., Gyotoku J., Morishige F., Pauling L. (1983). Enhancement of antitumor activity of ascorbate against Ehrlich ascites tumor cells by the copper:glycylglycylhistidine complex. Cancer Res..

[bib136] Kirvan G.E., Margerum D.W. (1985). Formation and NMR spectra of platinum(II)-tripeptide complexes. Inorg. Chem..

[bib137] Kleingardner J.G., Kandemir B., Bren K.L. (2014). Hydrogen evolution from neutral water under aerobic conditions catalyzed by cobalt microperoxidase-11. J. Am. Chem. Soc..

[bib138] Kodadek T. (2002). Development of protein-detecting microarrays and related devices. Trends Biochem. Sci..

[bib139] Kolozsi A., Jancsó A., Nagy N.V., Gajda T. (2009). N-terminal fragment of the anti-angiogenic human endostatin binds copper(II) with very high affinity. J. Inorg. Biochem..

[bib140] Konno K., Hisada M., Fontana R., Lorenzi C.C.B., Naoki H., Itagaki Y., Miwa A., Kawai N., Nakata Y., Yasuhara T. (2001). Anoplin, a novel antimicrobial peptide from the venom of the solitary wasp Anoplius samariensis. Biochim. Biophys. Acta Protein Struct. Mol. Enzymologia.

[bib141] Kotuniak R., Strampraad M.J.F., Bossak-Ahmad K., Wawrzyniak U.E., Ufnalska I., Hagedoorn P.L., Bal W. (2020). Key intermediate species reveal the copper(II)-Exchange pathway in biorelevant ATCUN/NTS complexes. Angew. Chem. Int..

[bib142] Kozłowski H., Bal W., Dyba M., Kowalik-Jankowska T. (1999). Specific structure–stability relations in metallopeptides. Coord. Chem. Rev..

[bib143] Krämer R. (1998). Fluorescent chemosensors for Cu^2+^ ions: fast, selective, and highly sensitive. Angew. Chem. Int..

[bib144] Kruck T.P.A., Lau S.J., Sarkar B. (1976). Molecular design to mimic the copper(II) transport site of human albumin: studies of equilibria between copper(II) and glycylglycyl-L-histidine-N-methyl amide and comparison with human albumin. Can. J. Chem..

[bib145] Lasorsa A., Nardella M.I., Rosato A., Mirabelli V., Caliandro R., Natile G., Arnesano F. (2019). Mechanistic and structural basis for inhibition of copper trafficking by platinum anticancer drugs. J. Am. Chem. Soc..

[bib146] Lau S.J., Kruck T.P.A., Sarkar B. (1974). A peptide molecule mimicking the copper(II) transport site of human serum albumin. A comparative study between the synthetic site and albumin. J. Biol. Chem..

[bib147] Laussac J.P., Sarker B. (1984). Characterization of the copper(II) and nickel(II) transport site of human serum albumin. Studies of copper(II) and nickel(II) binding to peptide 1-24 of human serum albumin by carbon-13 and proton NMR spectroscopy. Biochemistry.

[bib148] Lee I.H., Zhao C., Cho Y., Harwig S.S.L., Cooper E.L., Lehrer R.I. (1997). Clavanins, alpha-helical antimicrobial peptides from tunicate hemocytes. FEBS Lett..

[bib149] Liang Q., Ananias D.C., Long E.C. (1998). Ni(II)·Xaa-Xaa-His induced DNA Cleavage:  deoxyribose modification by a common “activated” intermediate derived from KHSO5, MMPP, or H2O2. J. Am. Chem. Soc..

[bib150] Libardo M.D., Cervantes J.L., Salazar J.C., AngelesBoza A.M. (2014). Improved bioactivity of antimicrobial peptides by addition of amino-terminal copper and nickel (ATCUN) binding motifs. ChemMedChem.

[bib151] Libardo M.D., Paul T.J., Prabhakar R., Angeles-Boza A.M. (2015). Hybrid peptide ATCUN-sh-Buforin: influence of the ATCUN charge and stereochemistry on antimicrobial activity. Biochimie.

[bib152] Lihi N., Sanna D., Bányai I., Várnagy K., Sóvágó I. (2017). Unusual binding modes in the copper(ii) and palladium(ii) complexes of peptides containing both histidyl and cysteinyl residues. New J. Chem..

[bib153] Liu X., Wang F. (2012). Transition metal complexes that catalyze oxygen formation from water: 1979–2010. Coord. Chem. Rev..

[bib154] Liu J., Chakraborty S., Hosseinzadeh P., Yu Y., Tian S., Petrik I., Bhagi A., Lu Y. (2014). Metalloproteins containing cytochrome, iron–sulfur, Or Copper Redox Centers. Chem. Rev..

[bib155] Lowe J., Taveira-da-Silva R., Hilário-Souza E. (2017). Dissecting copper homeostasis in diabetes mellitus. IUBMB Life.

[bib156] Lubitz W., Ogata H., Rudiger O., Reijerse E. (2014). Hydrogenases. Chem. Rev..

[bib157] Lukavsky P.J., Kim I., Otto G.A., Puglisi J.D. (2003). Structure of HCV IRES domain II determined by NMR. Nat. Struct. Biol..

[bib158] MacBeath G., Schreiber S.L. (2000). Printing proteins as microarrays for high-throughput function determination. Science.

[bib159] Mack D.P., Iverson B.L., Dervan P.B. (1988). Design and chemical synthesis of a sequence-specific DNA-cleaving protein. J. Am. Chem. Soc..

[bib160] Mack D.P., Dervan P.B. (1990). Nickel-mediated sequence-specific oxidative cleavage of DNA by a designed metalloprotein. J. Am. Chem. Soc..

[bib161] Mack D.P., Dervan P.B. (1992). Sequence-specific oxidative cleavage of DNA by a designed metalloprotein, nickel(II).cntdot.GGH(Hin139-190). Biochemistry.

[bib162] Mahmoudi T., Sarkar B. (1999). Addition of positively charged tripeptide to N-terminus of the Fos basic region leucine zipper domain: implications on DNA bending, affinity, and specificity. Biopolymers.

[bib163] Maiti B.K., Almeida R.M., Moura I., Moura J.J.G. (2017). Rubredoxins derivatives: simple sulphur-rich coordination metal sites and its relevance for Biology and Chemistry. Coord. Chem. Rev..

[bib164] Maiti B.K., Almeida R.M., Maia L.B., Moura I., Moura J.J.G. (2017). Insights into the molybdenum/copper heterometallic cluster Assembly in the orange protein: probing intermolecular interactions with an artificial metal-binding ATCUN tag. Inorg. Chem..

[bib165] Maiti B.K., Maia L.B., Moro A.J., Lima J.C., Cordas C.M., Moura I., Moura J.J.G. (2018). Unusual reduction mechanism of copper in cysteine-rich environment. Inorg. Chem..

[bib166] Maiti B.K., Maia L.B., Moura I., Moura J.J.G. (2019). Ni^II^-ATCUN-Catalyzed tyrosine nitration in the presence of nitrite and sulfite. Chem. Eur. J..

[bib167] Maiti B.K. (2020). Potential role of peptide-based antiviral therapy against SARSCoV-2 infection. ACS Pharmacol. Transl. Sci..

[bib168] Maiti B.K., Moura J.J.G. (2020). Diverse biological roles of the tetrathiomolybdate anion. Crood. Chem. Rev..

[bib169] Mal T.K., Ikura M., Kay L.E. (2002). The ATCUN domain as a probe of intermolecular Interactions:  application to Calmodulin−Peptide complexes. J. Am. Chem. Soc..

[bib170] Mania D., Hilpert K., Ruden S., Fischer R., Takeshita N. (2010). Screening for antifungal peptides and their modes of action in Aspergillus nidulans. Appl. Environ. Microbiol..

[bib171] Martino M.C.D., Hofland L.J., Lamberts S.W.J. (2010). Chapter 11-somatostatin and somatostatin receptors: from basic concepts to clinical applications. Prog. Brain Res..

[bib172] McCaslin T.G., Pagba C.V., Yohannan J., Barry B.A. (2019). Specific metallo-protein interactions and antimicrobial activity in Histatin-5, an intrinsically disordered salivary peptide. Sci. Rep..

[bib173] McDonald M.R., Fredericks F.C., Margerum D.W. (1997). Characterization of copper(III)−Tetrapeptide complexes with histidine as the third residue. Inorg. Chem..

[bib174] McEvoy J.P., Brudvig G.W. (2006). Water-splitting Chemistry Photosystem II. Chem. Rev..

[bib175] McGrath D.M., Barbu E.M., Driessen W.H.P., Lasco T.M., Tarrand J.J., Okhuysen P.C., Kontoyiannis D.P., Sidman R.L., Pasqualini R., Arap W. (2013). Mechanism of action and initial evaluation of a membrane active all-D-enantiomer antimicrobial peptidomimetic. Proc. Natl. Acad. Sci. U S A.

[bib176] McGrath M.E., Hines W.M., Sakanari J.A., Fletterick R.J., Craik C.S. (1991). The sequence and reactive site of ecotin. A general inhibitor of pancreatic serine proteases from Escherichia coli. J. Biol. Chem..

[bib177] Mckay D.J., Renaux B.S., Dixon G.H. (1986). Human sperm protamines Amino-acid sequences of two forms of protamine P2. Eur. J. Biochem..

[bib178] Melino S., Gallo M., Trotta E., Mondello F., Paci M., Petruzzelli R. (2006). Metal-binding and nuclease activity of an antimicrobial peptide analogue of the salivary histatin 5. Biochemistry.

[bib179] Meng J., Zhang H., Dong X., Liu F., Sun Y. (2018). RTHLVFFARK-NH2: a potent and selective modulator on Cu2+-mediated amyloid-β protein aggregation and cytotoxicity. J. Inorg. Biochem..

[bib180] Mestre B., Jakobs A., Pratviel G., Meunier B. (1996). Structure/nuclease activity relationships of DNA cleavers based on cationic metalloporphyrin-oligonucleotide conjugates. Biochemistry.

[bib181] Meunier S., Strable E., Finn M.G. (2004). Crosslinking of and coupling to viral capsid proteins by tyrosine oxidation. Chem. Biol..

[bib182] Meyer F., Tolman W.B. (2015). Forums on small-molecule activation: from biological principles to energy applications. Inorg. Chem..

[bib183] Michon T., Chenu M., Kellershon N., Desmadril M., Guéguen J. (1997). Horseradish peroxidase oxidation of tyrosine-containing peptides and their subsequent Polymerization:  A kinetic study. Biochemistry.

[bib184] Mital M., Wezynfeld N.E., Frączyk T., Wiloch M.Z., Wawrzyniak U.E., Bonna A., Tumpach C., Barnham K.J., Haigh C.L., Bal W., Drew S.C. (2015). A functional role for Aβ in metal homeostasis? N-truncation and high-affinity copper binding. Angew. Chem. Int..

[bib185] Miyamoto T., Fukino Y., Kamino S., Ueda M., Enomoto S. (2016). Enhanced stability of Cu2+–ATCUN complexes under physiologically relevant conditions by insertion of structurally bulky and hydrophobic amino acid residues into the ATCUN motif. Dalton Trans..

[bib186] Mjos K.D., Orvig C. (2014). Metallodrugs in medicinal inorganic chemistry. Chem. Rev..

[bib187] Mondal T., Ray U., Manna A.K., Gupta R., Roy S., Das S. (2008). Structural determinant of human La protein critical for internal initiation of translation of hepatitis C virus RNA. J. Virol..

[bib188] Moore C.M., Szymczak N.K. (2015). Nitrite reduction by copper through ligand-mediated proton and electron transfer. Chem. Sci..

[bib189] Muller J.G., Hickerson R.P., Perez R.J., Burrows C.J. (1997). DNA damage from sulfite autoxidation catalyzed by a nickel(II) peptide. J. Am. Chem. Soc..

[bib190] Murray C.K., Margerum D.W. (1982). Axial coordination of monodentate ligands with nickel(III) peptide complexes. Inorg. Chem..

[bib191] Nadai M., Doria F., Scalabrin M., Pirota V., Grande V., Bergamaschi G., Amendola V., Winnerdy F.R., Phan A.T., Richter S.N., Freccero M. (2018). A catalytic and selective scissoring molecular tool for quadruplex nucleic acids. J. Am. Chem. Soc..

[bib192] Nagane R., Koshigoe T., Chikira M., Long E.C. (2001). The DNA-bound orientation of Cu(II)·Xaa-Gly-His metallopeptides. J. Inorg. Biochem..

[bib193] Najmudin S., Bonifácio C., Duarte A.G., Pualeta S.R., Moura I., Moura J.J.G., Romão M.J. (2009). Crystallization and crystallographic analysis of the apo form of the orange protein (ORP) from Desulfovibrio gigas. Acta Cryst..

[bib194] Natesh R., Schwager S.L., Sturrock E.D., Acharya K.R. (2003). Crystal structure of the human angiotensin-converting enzyme-lisinopril complex. Nature.

[bib195] Neidle S. (2017). Quadruplex nucleic acids as targets for anticancer therapeutics. Nat. Rev. Chem..

[bib196] Neupane K.P., Aldous A.R., Kritzer J.A. (2013). Macrocyclization of the ATCUN motif controls metal binding and catalysis. Inorg. Chem..

[bib197] Neupane K.P., Aldous A.R., Kritzer J.A. (2014). Metal-binding and redox properties of substituted linear and cyclic ATCUN motifs. J. Inorg. Biochem..

[bib198] Neta P., Huie R.E. (1985). Free-radical chemistry of sulfite. Environ. Health Perspect..

[bib199] Otting G. (2010). Protein NMR using paramagnetic ions. Annu. Rev. Biophys..

[bib200] Pamatong F.V., Detmer C.A., Bocarsly J.R. (1996). Double-strand cleavage of DNA by a monofunctional transition metal cleavage agent. J. Am. Chem. Soc..

[bib201] Pap J.S., Szyrwiel Ł., Srankó D., Kerner Z., Setner B., Szewczuk Z., Malinka W. (2015). Electrocatalytic water oxidation by CuII complexes with branched peptides. Chem. Commun..

[bib202] Patwardhana A., Cowan J.A. (2001). Highly specific oxidative damage of double-strand DNA by copper aminoglycosidesElectronic supplementary information (ESI) available: experimental section and complete HPLC trace for the truncated version shown in Fig. 2B. Chem. Commun..

[bib203] Park C.B., Kim M.S., Kim S.C. (1996). A novel antimicrobial peptide from Bufo bufo gargarizans. Biochem. Biophys. Res. Commun..

[bib204] Park C.B., Yi K.S., Matsuzaki K., Kim M.S., Kim S.C. (2000). Structure–activity analysis of buforin II, a histone H2A-derived antimicrobial peptide: the proline hinge is responsible for the cell-penetrating ability of buforin II. Proc. Natl. Acad. Sci. U S A.

[bib205] Pauleta S.R., Duarte A.G., Carepo M.S., Pereira A.S., Tavares P., Moura I., Moura J.J.G. (2007). NMR assignment of the apo-form of a Desulfovibrio gigas protein containing a novel Mo–Cu cluster. Biomol. NMR Assign..

[bib206] Pearson N.D., Prescott C.D. (1997). RNA as a drug target. Chem. Biol..

[bib207] Person M.D., Brown K.C., Mahrus S., Craik C.S., Burlingame A.L. (2001). Novel inter-protein cross-link identified in the GGH-ecotin D137Y dimer. Protein Sci..

[bib208] Petrik J. (2001). Microarray technology: the future of blood testing?. Vox Sang.

[bib209] Pettit L.D., Pyburn S., Bal W., Kozlowski H., Bataille M. (1990). A study of the comparative donor properties to Cu^II^ of the terminal amino and imidazole nitrogens in peptides. J. Chem. Soc. Dalton Trans..

[bib210] Phillips C.L., Thrower J., Pickart C.M., Hill C.P. (2001). Structure of a new crystal form of tetraubiquitin. Acta Cryst..

[bib211] Piccioli M., Turano P. (2015). Transient iron coordination sites in proteins: exploiting the dual nature of paramagnetic NMR. Coord. Chem. Rev..

[bib212] Pinkham A.M., Yu Z., Cowan J.A. (2018). Attenuation of West nile virus NS2B/NS3 protease by amino terminal copper and nickel binding (ATCUN) peptides. J. Med. Chem..

[bib213] Pinkham A.M., Yu Z., Cowan J.A. (2018). Broad-spectrum catalytic metallopeptide inactivators of Zika and West Nile virus NS2B/NS3 proteases. Chem. Commun..

[bib214] Płonka D., Bal W. (2017). The N-terminus of hepcidin is a strong and potentially biologically relevant Cu(II) chelator. Inorg. Chim. Acta.

[bib215] Pogozelski W.J., McNeese T.J., Tullius T.D. (1995). What species is responsible for strand scission in the reaction of [Fe^II^EDTA]^2-^ and H_2_O_2_ with DNA?. J. Am. Chem. Soc..

[bib216] Pogozelski W.K., Tullius T.D. (1998). Oxidative strand scission of nucleic Acids:  routes initiated by hydrogen abstraction from the sugar moiety. Chem. Rev..

[bib217] Portelius E., Bogdanovic N., Gustavsson M.K., Volkmann I., Brinkmalm G., Zetterberg H., Winblad B., Blennow K. (2010). Mass spectrometric characterization of brain amyloid beta isoform signatures in familial and sporadic Alzheimer's disease. Acta Neuropathol..

[bib218] Pudi R., Abhiman S., Srinivasan N., Das S. (2003). Hepatitis C virus internal ribosome entry site-mediated translation is stimulated by specific interaction of independent regions of human La autoantigen. J. Biol. Chem..

[bib219] Pushie M.J., Stefaniak E., Sendzik M.R., Sokaras D., Kroll T., Haas K.L. (2019). Using N-terminal coordination of Cu(II) and Ni(II) to isolate the coordination environment of Cu(I) and Cu(II) bound to His13 and His14 in amyloid-β(4-16). Inorg. Chem..

[bib220] Radi R. (2013). Protein tyrosine nitration: biochemical mechanisms and structural basis of functional effects. Acc. Chem. Res..

[bib221] Reyes J.F., Fu Y., Vana L., Kanaan N.M., Binder L.I. (2011). Tyrosine nitration within the proline-rich region of Tau in Alzheimer's disease. Am. J. Pathol..

[bib222] Robert A., Liu Y., Nguyen M., Meunier B. (2015). Regulation of copper and iron homeostasis by metal chelators: a possible chemotherapy for Alzheimer’s disease. Acc. Chem. Res..

[bib223] Rosca V., Duca M., de Groot M.T., Koper M.T. (2009). Nitrogen cycle electrocatalysis. Chem. Rev..

[bib224] Ross M.J., Bradford S.S., Cowan J.A. (2015). Catalytic metallodrugs based on the LaR2C peptide target HCV SLIV IRES RNA. Dalton Trans..

[bib225] Ross M.J., Fidai I., Cowan J.A. (2017). Analysis of structure-activity relationships based on the hepatitis C virus SLIIb internal ribosomal entry sequence RNA-targeting GGHYRFK⋅Cu complex. ChemBioChem.

[bib226] Rozga M., Sokołowska M., Protas A.M., Bal W. (2007). Human serum albumin coordinates Cu(II) at its N-terminal binding site with 1 pM affinity. J. Biol. Inorg. Chem..

[bib227] Samii A., Bickel U., Stroth U., Pardridge W.M. (1994). Blood-brain barrier transport of neuropeptides: analysis with a metabolically stable dermorphin analogue. Am. J. Physiol..

[bib228] Sánchez-Ferrer A., Rodríguez-López J.N., García-Cánovas F., García-Carmona F. (1995). Tyrosinase: a comprehensive review of its mechanism. Biochim. Biophys. Acta.

[bib229] Sankararamakrishnan R., Verma S., Kumar S. (2005). ATCUN-like metal-binding motifs in proteins: identification and characterization by crystal structure and sequence analysis. Proteins.

[bib230] Santoro A., Walke G., Vileno B., Kulkarni P.P., Raibauta L., Faller P. (2018). Low catalytic activity of the Cu(ii)-binding motif (Xxx-Zzz-His; ATCUN) in reactive oxygen species production and inhibition by the Cu(i)-chelator BCS. Chem. Commun..

[bib231] Santoro A., Calvo J.S., Peris-Díaz M.D., Krężel A., Meloni G., Faller P. (2020). The glutathione/metallothionein system challenges the design of efficient O_2_-activating copper complexes. Angew. Chem. Int..

[bib232] Sendzik M., Pushie M.J., Stefaniak E., Haas K.L. (2017). Structure and affinity of Cu(I) bound to human serum albumin. Inorg. Chem..

[bib233] Sfeir A., Kosiyatrakul S.T., Hockemeyer D., MacRae S.L., Karlseder J., Schildkraut C.L., de Lange T. (2009). Mammalian telomeres resemble fragile sites and require TRF1 for efficient replication. Cell.

[bib234] Shin D.H., Song H.K., Seong I.S., Lee C.S., Chung C.H., Suh S.W. (1996). Crystal structure analyses of uncomplexed ecotin in two crystal forms: implications for its function and stability. Protein Sci..

[bib235] Sigman D.S. (1986). Nuclease activity of 1,10-phenanthroline-copper ion. Acc. Chem. Res..

[bib236] Sokolowska M., Krezel A., Dyba M., Szewczuk Z., Bal W. (2002). Short peptides are not reliable models of thermodynamic and kinetic properties of the N-terminal metal binding site in serum albumin. Eur. J. Biochem..

[bib237] Solomon E.I., Heppne D.E., Johnston E.M., Ginsbach J.W., Cirera J., Qayyum M., Kieber-Emmons M.T., Kjaergaard C.H., Hadt R.G., Tian L. (2014). Copper active sites in biology. Chem. Rev..

[bib238] Soubrier F., Alhenc-Gelas F., Hubert C., Allegrini J., John M., Tregear G., Corvol P. (1998). Two putative active centers in human angiotensin I-converting enzyme revealed by molecular cloning. Proc. Natl. Acad. Sci. U S A.

[bib239] Sóvágó I., Ősza K. (2006). Metal ion selectivity of oligopeptides. Dalton Trans..

[bib240] Sóvágó I., Várnagy K., Lihi N., Grenács Á. (2016). Coordinating properties of peptides containing histidyl residues. Coord. Chem. Rev..

[bib241] Sundberg R.J., Martin R.B. (1974). Interactions of histidine and other imidazole derivatives with transition metal ions in chemical and biological systems. Chem. Rev..

[bib242] Sun L.C., Coy D.H. (2011). Somatostatin receptor-targeted anti-cancer therapy. Curr. Drug Deliv..

[bib243] Sun Y., Bigi J.P., Piro N.A., Tang M.L., Long J.R., Chang C.J. (2011). Molecular cobalt pentapyridine catalysts for generating hydrogen from water. J. Am. Chem. Soc..

[bib244] Supuran C.T., Scozzafava A., Casini A. (2003). Carbonic anhydrase inhibitors. Med. Res. Rev..

[bib245] Supuran C.T. (2008). Carbonic anhydrases: novel therapeutic applications for inhibitors and activators. Nat. Rev. Drug Discov..

[bib246] Suree N., Liew C.K., Villareal V.A., Thieu W., Fadeev E.A., Clemens J.J., Jung M.E., Clubb R.T. (2009). The structure of the Staphylococcus aureus sortase-substrate complex reveals how the universally conserved LPXTG sorting signal is recognized. J. Biol. Chem..

[bib247] Stadtman E.R. (1993). Oxidation of free amino acids and amino acid residues in proteins by radiolysis and by metal-catalyzed reactions. Annu. Rev. Biochem..

[bib248] Szyrwiel Ł., Lukács D., Srankó D.F., Kerner Z., Kotynia A., Brasuń J., Setner B., Szewczuk Z., Malec K., Pap J.S. (2017). Armed by Asp? C-terminal carboxylate in a Dap-branched peptide and consequences in the binding of CuII and electrocatalytic water oxidation. RSC Adv..

[bib249] Thornberry N.A., Rano T.A., Peterson E.P., Rasper D.M., Timkey T., Garcia-Calvo M., Houtzager V.M., Nordstrom P.A., Roy S., Vaillancourt J.P. (1997). A combinatorial approach defines specificities of members of the caspase family and granzyme B. Functional relationships established for key mediators of apoptosis. J. Biol. Chem..

[bib250] Thorp H.H. (2000). The importance of being r: greater oxidative stability of RNA compared with DNA. Chem. Biol..

[bib251] Tian J., Liu J., Tian X., Hu Z., Chen X. (2004). Study of the interaction of kaempferol with bovine serum albumin. J. Mol. Struct..

[bib252] Timmons A.J., Symes M.D. (2015). Converting between the oxides of nitrogen using metal–ligand coordination complexes. Chem. Soc. Rev..

[bib253] Torrado A., Walkup G.K., Imperiali B. (1998). Exploiting polypeptide motifs for the design of selective Cu(II) ion chemosensors. J. Am. Chem. Soc..

[bib254] Uyeda C., Peters J.C. (2013). Selective nitrite reduction at heterobimetallic CoMg complexes. J. Am. Chem. Soc..

[bib255] Viles J.H., Cohen F.E., Prusiner S.B., Goodin D.B., Wright P.E., Dyson H.J. (1999). Copper binding to the prion protein: structural implications of four identical cooperative binding sites. Proc. Natl. Acad. Sci. U S A.

[bib256] Wadas T.J., Wong E.H., Weisman G.R., Anderson C.J. (2010). Coordinating radiometals of copper, gallium, indium, Yttrium, and zirconium for PET and SPECT imaging of disease. Chem. Rev.

[bib257] Walke G.R., Ruthstein S. (2019). Does the ATSM-Cu(II) biomarker integrate into the human cellular copper cycle?. ACS Omega.

[bib258] Wendea C., Kulak N. (2015). Fluorophore ATCUN complexes: combining agent and probe for oxidative DNA cleavage. Chem. Commun..

[bib259] Wezynfeld N.E., Stefaniak E., Stachucy K., Drozd A., Płonka D., Drew S.C., Krężel A., Bal W. (2016). Resistance of Cu(Aβ4–16) to copper capture by metallothionein-3 supports a function for the aβ4–42 peptide as a synaptic CuII scavenger. Angew. Chem. Int.

[bib260] Wiloch M.Z., Ufnalska I., Bonna A., Bal W., Wróblewski W., Wawrzyniak U.E. (2017). Copper(II) complexes with ATCUN peptide analogues: studies on redox activity in different solutions. J. Electrochem. Soc..

[bib261] Wood P.M. (1988). The potential diagram for oxygen at pH 7. Biochem. J..

[bib262] Wrapp D., Wang N., Corbett K.S., Goldsmith J.A., Hsieh C.L., Abiona O., Graham B.S., McLellan J.S. (2020). Cryo-EM structure of the 2019-nCoV spike in the prefusion conformation. Science.

[bib263] Xie D., King T.L., Banerjee A., Kohli V., Que E.L. (2016). Exploiting copper redox for ^19^F magnetic resonance-based detection of cellular hypoxia. J. Am. Chem. Soc..

[bib264] Yan R., Zhang Y., Li Y., Xia L., Guo Y., Zhou Q. (2020). Structural basis for the recognition of SARS-CoV-2 by full-length human ACE2. Science.

[bib265] Yano J., Yachandra V. (2014). Mn4Ca cluster in photosynthesis: where and how water is oxidized to dioxygen. Chem. Rev..

[bib266] Young T.R., Kirchner A., Wedd A.G., Xiao Z. (2014). An integrated study of the affinities of the Aβ16 peptide for Cu(i) and Cu(ii): implications for the catalytic production of reactive oxygen species. Metallomics.

[bib267] Young T.R., Wijekoon C.J.K., Spyrou B., Donnelly P.S., Wedd A.G., Xiao Z. (2015). A set of robust fluorescent peptide probes for quantification of Cu(II) binding affinities in the micromolar to femtomolar range. Metallomics.

[bib268] Yu D., Volkov A.N., Tang C. (2009). Characterizing dynamic Protein−Protein interactions using differentially scaled paramagnetic relaxation enhancement. J. Am. Chem. Soc..

[bib269] Yu F., Cangelosi V.M., Zastrow M.L., Tegoni M., Plegaria J.S., Tebo A.G., Mocny C.S., Ruckthong L., Qayyum H., Pecoraro V.L. (2014). Protein design: toward functional metalloenzymes. Chem. Rev..

[bib270] Yu M.A., Egawa T., Shinzawa-Itoh K., Yoshikawa S., Guallar V., Yeh S.R., Rousseau D.L., Gerfen G.J. (2012). Two tyrosyl radicals stabilize high oxidation states in cytochrome c oxidase for efficient energy conservation and proton translocation. J. Am. Chem. Soc..

[bib271] Yu Z., Han M., Cowan J.A. (2015). Toward the design of a catalytic metallodrug: selective cleavage of G-quadruplex telomeric DNA by an anticancer copper-acridine-ATCUN complex. Angew. Chem. Int..

[bib272] Yu Z., Cowan J.A. (2017). Catalytic metallodrugs: substrate-selective metal catalysts as therapeutics. Chem. Eur. J..

[bib273] Yu Z., Cowan J.A. (2017). Design of artificial glycosidases: metallopeptides that remove H antigen from human erythrocytes. Angew. Chem. Int..

[bib274] Yu Z., Fenk K.D., Huang D., Sen S., Cowan J.A. (2019). Rapid telomere reduction in cancer cells induced by G-quadruplex-targeting copper complexes. J. Med. Chem..

[bib275] Zahler A.M., Williamson J.R., Cech T.R., Prescott D.M. (1991). Inhibition of telomerase by G-quartet DNA structures. Nature.

[bib276] Zasloff M. (2002). Antimicrobial peptides of multicellular organisms. Nature.

[bib277] Zhang M.-T., Chen Z., Kang P., Meyer T.J. (2013). Electrocatalytic water oxidation with a copper(II) polypeptide complex. J. Am. Chem. Soc..

[bib278] Zhang Q., Hu X., Wang W., Yuan Z. (2016). Study of a bifunctional Aβ aggregation inhibitor with the abilities of antiamyloid-β and copper chelation. Biomacromolecules.

[bib279] Zhao S., Eriksson L.A., Zhang R.-B. (2018). Theoretical insights on the inefficiency of RNA oxidative damage under aerobic conditions. J. Phys. Chem. A..

[bib280] Zheng Y., Gattás-Asfura K.M., Konka V., Leblanc R.M. (2002). A dansylated peptide for the selective detection of copper ions. Chem. Commun.

[bib281] Zong Y., Bice T.W., Ton-That H., Schneewind O., Narayana S.V. (2004). Crystal structures of Staphylococcus aureus sortase A and its substrate complex. J. Biol. Chem..

